# Evolution and targeting of Omp85 homologs in the chloroplast outer envelope membrane

**DOI:** 10.3389/fpls.2014.00535

**Published:** 2014-10-13

**Authors:** Philip M. Day, Daniel Potter, Kentaro Inoue

**Affiliations:** Department of Plant Sciences, University of California at DavisDavis, CA, USA

**Keywords:** β-barrel, chloroplast evolution, OEP80, Omp85, outer membrane, protein import, POTRA, Toc75

## Abstract

Translocon at the outer-envelope-membrane of chloroplasts 75 (Toc75) is the core component of the chloroplast protein import machinery. It belongs to the Omp85 family whose members exist in various Gram-negative bacteria, mitochondria, and chloroplasts of eukaryotes. Chloroplasts of Viridiplantae contain another Omp85 homolog called outer envelope protein 80 (OEP80), whose exact function is unknown. In addition, the *Arabidopsis thaliana* genome encodes truncated forms of Toc75 and OEP80. Multiple studies have shown a common origin of the Omp85 homologs of cyanobacteria and chloroplasts but their results about evolutionary relationships among cyanobacterial Omp85 (cyanoOmp85), Toc75, and OEP80 are inconsistent. The bipartite targeting sequence-dependent sorting of Toc75 has been demonstrated but the targeting mechanisms of other chloroplast Omp85 homologs remain largely unexplored. This study was aimed to address these unresolved issues in order to further our understanding of chloroplast evolution. Sequence alignments and recently determined structures of bacterial Omp85 homologs were used to predict structures of chloroplast Omp85 homologs. The results enabled us to identify amino acid residues that may indicate functional divergence of Toc75 from cyanoOmp85 and OEP80. Phylogenetic analyses using Omp85 homologs from various cyanobacteria and chloroplasts provided strong support for the grouping of Toc75 and OEP80 sister to cyanoOmp85. However, this support was diminished when the analysis included Omp85 homologs from other bacteria and mitochondria. Finally, results of import assays using isolated chloroplasts support outer membrane localization of OEP80tr and indicate that OEP80 may carry a cleavable targeting sequence.

## Introduction

**Figure 1:**
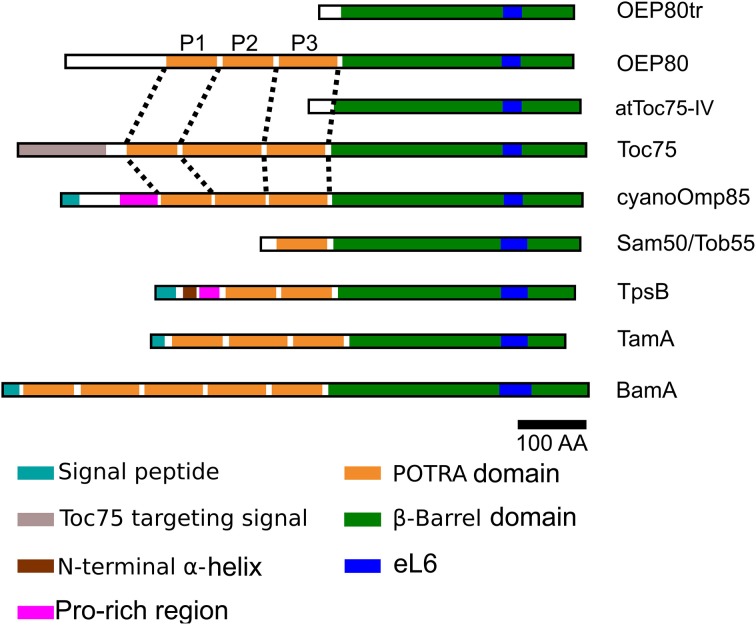
**Molecular architecture of the Omp85 family proteins**. Omp85 homologs are comprised of an N-terminal soluble portion containing a variable number of POTRA domains (orange) followed by a C-terminal transmembrane β-barrel (green). The POTRA domains 1, 2, and 3 of the chloroplast and cyanobacteria homologs are indicated as P1, P2, and P3, respectively. Unlike the others, OEP80tr and Toc75-IV do not contain any full POTRA domains although they both contain sequences that align well with the last β-strand of P3 in their relatives. Within the β-barrel that is made of 16 transmembrane β-strands, strands 11 and 12 are separated by the 6th extracellular loop (blue) which contains a sequence highly conserved between all Omp85 homologs. The homologs from bacteria contain a signal peptide (turquoise) that is required for protein export from the cytoplasm to the periplasm. Toc75 contains a unique bipartite targeting signal (gray) at its N terminus and an apparent unique insertion at the beginning of its second POTRA domain. Cyanobacterial Omp85 homologs and TpsB contain a Pro-rich region (pink) N terminus to the first POTRA domain. In addition, TpsB contains an α-helix (brown) N terminus to the Pro-rich region. The domain lengths are approximately to scale.

Chloroplasts are derived from an endosymbiotic relationship between an ancestral cyanobacterium and a mitochondriate eukaryote which occurred around 1 billion years ago (Shih and Matzke, 2013). One piece of evidence to support a common ancestry of Gram-negative bacteria, mitochondria and chloroplasts is the presence of β-barrel proteins in their outer membranes (Inoue, 2007). Among β-barrel membrane proteins are the homologs of outer membrane protein 85 (Omp85) which appear to be present in all Gram-negative bacteria, mitochondria and chloroplasts (Voulhoux et al., 2003; Gentle et al., 2004, 2005; Voulhoux and Tommassen, 2004). A canonical member of the Omp85 family is comprised of a soluble N terminus which contains a variable number of polypeptide translocation associated (POTRA) domains, each of which usually consists of 70–90 residues, followed by a C-terminal transmembrane β-barrel (Sanchez-Pulido et al., 2003) (Figure 1). Results of various studies indicate that the POTRA domains are involved in association with other proteins while the β-barrel acts as an integral membrane anchor and may provide a hydrophilic pore to accommodate substrate proteins (Knowles et al., 2009; Kim et al., 2012; Simmerman et al., 2014).

Among bacterial Omp85 homologs are β-barrel assembly machinery A (BamA), two-partner secretion B (TpsB), and translocation and assembly module A (TamA). BamA is the central and essential component of the BAM complex, which catalyzes assembly of β-barrel proteins in the outer membranes (Knowles et al., 2009). BamA carries five POTRAs and has been shown to be indispensable for cell viability in *Neisseria meningitidis* and *Escherichia coli* (Voulhoux et al., 2003; Doerrler and Raetz, 2005) (Figure 1). TpsB and TamA are non-essential proteins found in a limited number of bacteria where they catalyze secretion of a subset of β-helical adhesion proteins known as TpsA and autotransporters, respectively (Jacob-Dubuisson et al., 2013). Canonical TpsB orthologs contain two POTRAs (Willems et al., 1994), while *E. coli* TamA contains three POTRAs (Selkrig et al., 2012) (Figure 1). Structural studies have revealed that each POTRA in various Omp85 homologs contains a conserved βααββ fold that forms a three-stranded β-sheet with two α-helices next to each other at one side of the sheet (Clantin et al., 2007; Kim et al., 2007; Gatzeva-Topalova et al., 2008, 2010; Knowles et al., 2008; Zhang et al., 2011). The C-terminal transmembrane β-barrels of FhaC, TamA, and BamA have been shown to consist of 16 antiparallel β-strands connected by eight extracellular loops (eLs) and seven short turns (Ts). A large extracellular loop between transmembrane β-strands (TMβ) 11 and 12 called eL6 could insert into the β-barrel pore (Clantin et al., 2007; Gruss et al., 2013; Noinaj et al., 2013; Ni et al., 2014). In FhaC, eL6 was shown to extend through the barrel to the periplasmic side of the membrane (Clantin et al., 2007), while eL6 in BamA and TamA remain in the pore via electrostatic interactions with the inner barrel wall (Gruss et al., 2013; Noinaj et al., 2013; Ni et al., 2014). Furthermore, eLs in BamA were shown to form a dome to prevent free entry or leak of molecules into or from the barrel interior (Noinaj et al., 2013; Ni et al., 2014). Structural studies have also revealed a loose association and possible opening between TMβ1 and 16 in BamA and TamA (Gruss et al., 2013; Noinaj et al., 2013) and gating of the β-barrel by the most C-terminal POTRA (POTRA5, P5) in BamA (Noinaj et al., 2013). An Omp85 homolog from a cyanobacterium *Synechocystis* sp. PCC 6803 contains three POTRAs and was shown to be essential for cell viability (Bolter et al., 1998; Reumann et al., 1999) (Figure 1). Crystal structural analyses confirmed the conserved folding of the POTRA domains in the Omp85 homologs from two cyanobacterial species, *Nostoc* sp. PCC7120 (Koenig et al., 2010) and *Thermosynechococcus elongatus* (Arnold et al., 2010). It was noted that most cyanobacterial Omp85 (cyanoOmp85) homologs contain a Pro-rich region preceding P1 (Arnold et al., 2010), similar to the case in TpsB (Jacob-Dubuisson et al., 2013) (Figure 1), although the relevance of this feature is unknown. Finally, no study has tested whether cyanoOmp85 is functionally homologous to BamA, TamA, or TpsB.

In mitochondria, only one type of Omp85 homolog has been identified. It contains one POTRA and is called sorting and assembly machinery 50 (Sam50) or topogenesis of mitochondrial outer membrane β-barrel proteins 55 (Tob55). Similar to BamA, Sam50/Tob55 is essential for outer membrane biogenesis and cell viability (Kozjak et al., 2003; Paschen et al., 2003; Gentle et al., 2004) (Figure 1). In contrast to the case in mitochondria, multiple Omp85 homologs are found in chloroplasts. Among them are translocon at the outer-envelope-membrane of chloroplasts 75 (Toc75) and outer envelope protein 80 (OEP80) (Hsu and Inoue, 2009). Both proteins contain three POTRAs (Figure 1) and are essential for viability from the embryonic stage of the model plant *Arabidopsis thaliana* (Baldwin et al., 2005; Hust and Gutensohn, 2006; Patel et al., 2008). Toc75 acts as the core component of the protein import channel (Perry and Keegstra, 1994; Schnell et al., 1994; Hinnah et al., 1997). OEP80 was given its name because its *A. thaliana* ortholog (At5g19620) was originally predicted to be a 732-amino-acid protein of 80 kD (Inoue and Potter, 2004). OEP80 is also known as Toc75-V as it is encoded in the *A. thaliana* chromosome V (Eckart et al., 2002). The exact function of OEP80 is unknown (Inoue, 2011). Interestingly, an *A. thaliana* mutant that contained a T-DNA insertion between the codons for the first and second Met of the *OEP80* gene was viable (Patel et al., 2008). A recent study using an antibody against a recombinant 80-kD protein encoded by the OEP80 cDNA and a genetic complementation assay with the *oep80*-null mutant showed that OEP80 is actually a protein of *ca.* 70 kD in *A. thaliana* chloroplasts (Hsu et al., 2012). As discussed below, both Toc75 and OEP80 appear to have evolved from ancestral cyanoOmp85, which was most likely not involved in protein import. Establishment of a protein import system must have been essential for the transition of the endosymbiont to the organelle. This is because it allowed replacement of original gene copies on the prokaryotic chromosome by duplicated copies in the eukaryotic host nucleus. Thus, the gene duplication that gave rise to Toc75 and OEP80 must have been an important event for chloroplast evolution. However, the molecular basis of the neofunctionalization of Toc75 is largely unexplored.

In addition to Toc75 and OEP80, the *A. thaliana* genome encodes several proteins that appear to be truncated Omp85 homologs (Figure 1). Among them, Toc75-IV (At4g09080) is encoded on chromosome IV (Jackson-Constan and Keegstra, 2001). This protein lacks the POTRA domains of Toc75 and its gene knockout mutant was viable but showed abnormal etioplast structure in seedlings and delayed de-etiolation (Baldwin et al., 2005). The *A. thaliana* genome also encodes two truncated forms of OEP80 that lack POTRA, called Ath-P1 (At3g44160; named OEP80tr in this study) and Ath-P2 (At3g48620), respectively (Moslavac et al., 2005; Topel et al., 2012). Outer membrane localization of Toc75-IV was demonstrated (Baldwin et al., 2005) while that of OEP80tr homologs remains unknown.

Previous phylogenetic analyses have established that Toc75, OEP80, and cyanoOmp85 share a common ancestor (Gentle et al., 2004; Inoue and Potter, 2004; Moslavac et al., 2005). Interestingly, however, conclusions about the evolutionary relationship between the three groups were inconsistent among studies. Some of the early studies reported a closer relationship of OEP80 with cyanoOmp85 than with Toc75 (Eckart et al., 2002; Gentle et al., 2004; Baldwin et al., 2005), while others left the relationships within the three groups unresolved (Inoue and Potter, 2004; Moslavac et al., 2005). A more recent work showed a well-supported grouping of Toc75 and OEP80 within the cyanoOmp85 clade (Topel et al., 2012). Although these studies have used different sets of sequences, the exact reason for the inconsistent conclusions remains unknown.

All known proteins in the outer membranes of chloroplasts and mitochondria are encoded in the nucleus (Hofmann and Theg, 2005; Lee et al., 2014). Among them, Toc75 is unique in that it carries a cleavable N-terminal targeting sequence known as tp75 (Tranel et al., 1995) (Figure 1). tp75 is comprised of two parts. The first part acts as a stroma-targeting sequence and is cleaved by a stromal processing peptidase (Tranel and Keegstra, 1996; Inoue et al., 2001). The second part of tp75 is needed for envelope targeting (Tranel and Keegstra, 1996) and is removed by plastidic type I signal peptidase 1 (Plsp1) (Inoue et al., 2005; Shipman-Roston et al., 2010; Midorikawa et al., 2014). Within the second part is a polyglycine stretch, which was shown to be necessary for preventing Toc75 from entering the stroma (Inoue and Keegstra, 2003; Baldwin and Inoue, 2006). Toc75-IV does not seem to carry a cleavable targeting sequence (Baldwin et al., 2005). It remains unknown if and how OEP80 and its truncated forms are processed and targeted to the chloroplast outer membrane.

In this study, we attempted to address unanswered questions about chloroplast Omp85 homologs; namely the molecular bases for their diversification, their phylogenetic relationships with cyanoOmp85, and their chloroplast targeting. Based on the obtained results, we have attempted to generate novel hypotheses regarding molecular mechanisms of membrane protein evolution.

## Materials and methods

### Sequence collection, structural prediction, and phylogenetic analysis

Identification numbers and sources of all the sequences used for the analyses are listed in Table S1. CyanoOmp85 homologs were identified by BLASTP searches against the GenBank database (www.ncbi.nlm.nih.gov/genbank) (Wheeler et al., 2003; Benson et al., 2013) using the amino acid sequence of the Omp85 homolog from *Synechocystis* sp. PCC 6803 (slr1227) (Bolter et al., 1998; Reumann et al., 1999) as a query. Among homologs identified, we selected 26 sequences from 20 species that represent diverse clades within cyanobacteria according to previous works (Tomitani et al., 2006; Criscuolo and Gribaldo, 2011). All the sequences showed E values not larger than 5E-151. The chloroplast Omp85 homologs except those from the liverwort (*Marchantia polymorpha*) and loblolly pine (*Pinus taeda*) were identified in the GenBank and Phytozome (www.phytozome.net) (Goodstein et al., 2012) databases by either TBLASTN [for pea (*Pisum sativum*), white spruce (*Picea glauca*), Douglas fir (*Pseudotsuga menziesii*), and *Nitella mirabilis*] or BLASTP (for all others) searches using amino acid sequences of *A. thaliana* Toc75 (At3g46740), OEP80 (At5g19620), and OEP80tr (Ath-P1 = At3g44160) as queries. Among the identified sequences, the pea OEP80 sequence was obtained by assembly and manual adjustments of three transcriptome shotgun assembly (TSA) sequences (Franssen et al., 2011) and the white spruce Toc75 sequence was obtained by assembly of four expressed-sequence-tag and cDNA clones (Ralph et al., 2008; Rigault et al., 2011) using the Cap3 program (http://doua.prabi.fr/software/cap3) (Huang and Madan, 1999). cDNAs encoding the liverwort chloroplast Omp85 homologs were identified by TBLASTN searches against the genome and transcriptome databases generated by the *M. polymorpha* genome-sequencing project at the Joint Genome Institute (http://www.jgi.doe.gov/) using the amino acid sequences of OEP80 and Toc75 from *A. thaliana* and *P. patens* as queries (Drs. R. Nishihama and T. Kohchi, Kyoto University). The loblolly pine Omp85 sequences were identified by TBLASTN searches using the Norway spruce genome assembly and gene expression data resource (http://congenie.org/) (Nystedt et al., 2013). For further analyses, we selected 78 sequences from 28 archaeplastida species, which showed E values of less than or equal to 1E-14 (for the sequences from green lineages) or 0.055 (for red algal sequences). We also collected sequences of the following six well-studied Omp85 homologs from the GenBank database: BamA orthologs from *N. meningitidis* and *E. coli*, *E. coli* TamA, *B. pertussis* FhaC, yeast Sam50, and *N. crassa* Tob55.

Structural predictions of *A. thaliana* Toc75 and OEP80 were done using Phyre2 (http://www.sbg.bio.ic.ac.uk/phyre2) (Kelley and Sternberg, 2009). The two dimensional diagrams were made using a program available at the website (http://blog.pansapiens.com/2008/06/26/software-review-producing-two-dimensional-diagrams-of-membrane-proteins/) and manual editing.

For phylogenetic analysis, the sequences were aligned using the MAFFT program (http://mafft.cbrc.jp/alignment/software/) (Katoh and Standley, 2013). The full alignment can be found in Supplementary Material (Figure S1). For analyses shown in **Figures 3A,C**, poorly aligned sections that may be non-homologous or saturated with mutations were removed using the Gblocks server with setting options for a less stringent selection that allows smaller final blocks, gap positions within the final blocks, and less strict flanking positions (http://molevol.cmima.csic.es/castresana/Gblocks_server.html) (Castresana, 2000). Depending on the analysis, various sections of the alignment were subject to Bayesian inference in the program MRBAYES (http://mrbayes.sourceforge.net/) (Huelsenbeck and Ronquist, 2001; Ronquist et al., 2012) using the mixed amino acid model option as specified by the command prset aamodelpr=mixed. For each analysis, a 50% majority-rule consensus tree was generated for the 3002 trees produced by two runs of two million generations each, sampled every 1000 generations with a 25% burn in. The percentage of trees in which a clade appeared was interpreted as the posterior probability (*PP*) of that clade.

### cDNA clones

Plasmids encoding Toc75-IV and OEP80_Δ1−52_ from *A. thaliana* were in the pGEMTEasy vector (Promega, Madison, WI) (Baldwin et al., 2005; Patel et al., 2008), and the one for OEP80tr was in pUNI51 from Arabidopsis Biological Resource Center (Columbus, OH), respectively. The plasmid encoding *A. thaliana* Toc75 was generated by amplifying the coding sequences by PCR using the previously-reported plasmid (Inoue and Keegstra, 2003) as a template and transferred to an SP6 transcription vector compatible with the Gateway^®^ cloning system (Life Technologies, Carlsbad, CA) (Joshua Endow and Kentaro Inoue, unpublished). The plasmid encoding Tic22 (pET21d-pTic22) (Kouranov et al., 1999) was a kind gift of Dr. D. Schnell (University of Massachusetts, Amherst, MA).

### *In vitro* chloroplast import assay

Protein import assay was done using intact chloroplasts isolated from 10- to 13-day-old pea seedlings and [^35^S]-labeled proteins as previously described (Inoue and Potter, 2004; Inoue et al., 2006). The radiolabeled proteins were synthesized using T_N_T^®^ coupled reticulocyte lysate system (Promega) and T7 (for Toc75-IV and Tic22), T3 (for OEP80tr), SP6 (for Toc75, and OEP80) RNA polymerases with [^35^S]Met (Perkin Elmer, Waltham, MA). Post-import fractionation of chloroplasts was done as described (Inoue et al., 2006). For post-import protease treatment, the chloroplasts containing the imported proteins were treated with thermolysin or trypsin (both are from Sigma-Aldrich Corp, St. Louis, MO) at a 1:1 mass ratio with the amount of chlorophylls incubated in the import reaction in import buffer with (for thermolysin) or without (for trypsin) 1 mM CaCl_2_, respectively, for 30 min in the dark on ice (for thermolysin) or at room temperature (for trypsin). The protease reactions were quenched by adding EDTA to the final concentration of 5 mM (for thermolysin) or trypsin inhibitor at a 10:1 mass ratio of the inhibitor to the protease (for trypsin) in import buffer. For the energy-dependency assay, the reaction was done using translation products pre-treated with 50 U/mL apyrase (Sigma-Aldrich) at room temperature for 15 min.

## Results

### Structural prediction of the chloroplast Omp85 homologs

Recent structural studies of bacterial Omp85 homologs have revealed a conserved folding pattern of POTRA domains (Simmerman et al., 2014) and the unique organization of the transmembrane β-barrels (Clantin et al., 2007; Gruss et al., 2013; Noinaj et al., 2013; Ni et al., 2014). As a first step to gain molecular insights into the evolution of chloroplast Omp85 homologs, we made use of available data as well as alignment and prediction programs to identify residues that are conserved among them or unique to each of the subgroups.

**Figure 2:**
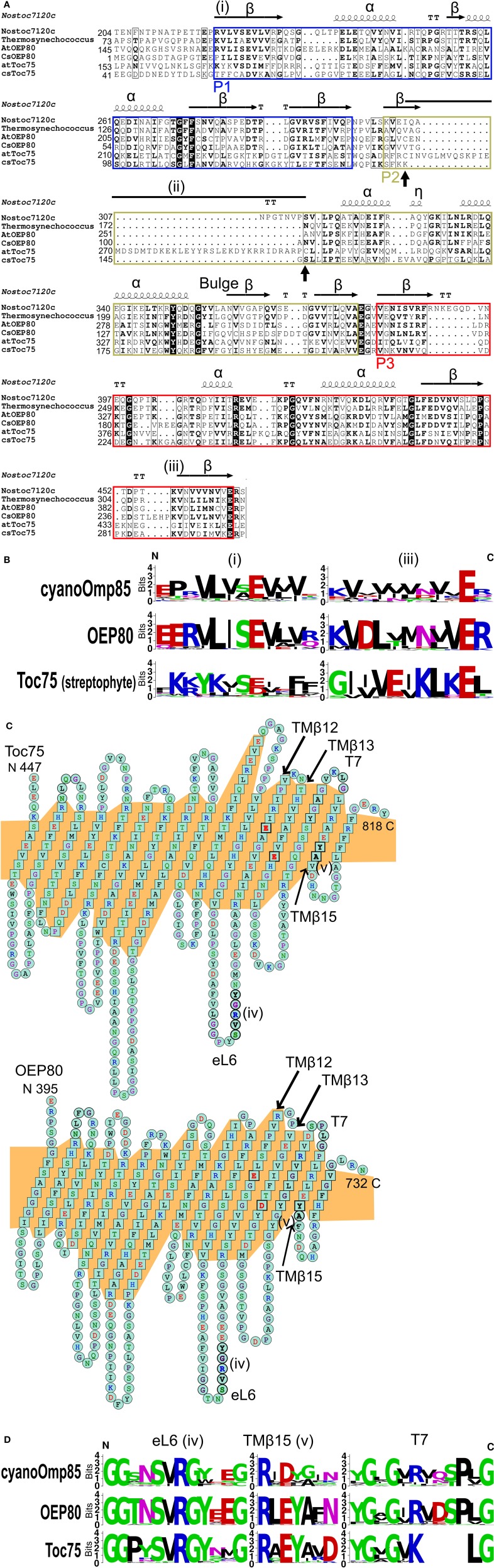
**Predicted structures of chloroplast Omp85 homologs. (A)** An alignment of the POTRA domains of two cyanoOmp85 orthologs with known structure and four chloroplast Omp85 homologs from the flowering plant *A. thaliana* (AtOEP80 and atToc75) and the green alga *Coccomyxa subellipsoidea* (CsOEP80 and csToc75). Shown above the alignment is the resolved secondary structure of Omp85 from *Nostoc* sp. PCC7120 (Koenig et al., 2010). POTRA domains 1–3 are indicated as P1-3, respectively. The horizontal arrows with β indicate β-strands. The coils show α-helices (indicated with α) or a 3–10 helix (indicated with η) found in the C terminus of the 1st helix of P2. The residues located at turns are indicated with T. Sequences conserved in OEP80 and cyanoOmp85 but diverged in Toc75 (i,iii) as well as an apparent insert at the N terminus of P2 of land plant Toc75 (ii) are indicated. The two vertical arrows below the alignment indicate the positions of Cys residues flanking the insert in Toc75. **(B)** Weblogos of regions indicated as (i,iii) in panel **(A)**. **(C)** Predicted transmembrane β-strands of *A. thaliana* Toc75 and OEP80 generated using the Phyre2 server (Kelley and Sternberg, 2009). The program threaded both proteins onto the known structure of *Neisseria gonorrhoeae* BamA. The N and C termini of the proteins are indicated at left and right, respectively. Residues in the transmembrane β-strands are indicated in squares and those in turns and loops are in circles. The highly conserved SVRGY motif in eL6 and Y/FA motif in TMβ15 are indicated boldtyped and as (iv,v), respectively. Glu on TMβ12 and Asp on TMβ13 are indicated as boldtyped. The orange background shows the predicted transmembrane region. **(D)** Weblogos of the conserved sequences shown as (iv,v) in panel **(C)** and the area surrounding turn 7 (T7) of cyanoOmp85, OEP80, and Toc75 examined in this study.

The N-terminal soluble portion of cyanoOmp85, Toc75 and OEP80 is comprised of three POTRAs called P1, P2, and P3 (Figure 1). Each POTRA shows the canonical secondary structure pattern known as β1-α1-α2-β2-β3, and P3 is the most conserved POTRA domain among the three (Arnold et al., 2010). The crystal structures and sequence alignments have also revealed features of POTRAs uniquely conserved between homologs from cyanobacteria and chloroplasts (Arnold et al., 2010; Koenig et al., 2010; Simmerman et al., 2014). Our analysis identified two features that could separate Toc75 from OEP80 and cyanoOmp85. The first is found at the N terminus of P1: Val^21^-Leu^22^ in *Nostoc* Omp85 is conserved as Val^62^-Leu^63^ in OEP80 but is diverged to Tyr^70^-Lys^71^ in Toc75 [Figures 2A,B, indicated by (i)]. These residues contribute to the β-cap in cyanoOmp85 P1 (Arnold et al., 2010; Koenig et al., 2010). Thus, a β-cap may also be present in P1 of OEP80 but not that in Toc75. The second feature unique to Toc75 is an extraordinary long P2 which was noted previously (Simmerman et al., 2014) [Figure 2A, indicated by (ii)]. Our analysis revealed that this is due to the insertion of 43 to 44 residues flanked by two Cys residues (Cys^256^ and Cys^300^ in *A. thaliana* Toc75; Figure 2A, indicated with vertical arrows). An alignment with known structures suggests that this insertion may disrupt the canonical β1 in P2 (Figure 2A). Generation or reduction of a disulfide bridge between the flanking Cys residues might affect stability of the three-stranded β-sheet in P2. This may lead to redox regulation of chloroplast protein import as predicted previously (Balsera et al., 2010). Interestingly, this insertion was not found in Toc75 from green and red algae (Figure 2A, csToc75; Figure S1). Another notable feature that could distinguish streptophyte Toc75 from the other Omp85 homologs can be found at β3 of P3 [Figures 2A,B, indicated by (iii); Figure S1]. Within this region is Lys^386^ in *A. thaliana* OEP80, which is highly conserved in OEP80 (22 out of 27 sequences examined in this study), cyanoOmp85 (15 out of 26 sequences) and the chlorophyte green algal Toc75 (5 out of 5) as well as in NmOmp85 and EcTamA. However, this Lys residue is not found in any of the Toc75 sequences from land plants examined in this study or the streptophyte green alga (*Nitella mirabilis*), where it is replaced by Gly (for 26 sequences) or Ser (for two sequences). The presence of features unique to Toc75 shows that Toc75 has continued to change throughout the evolution of plants. The overall identities of the sequences containing all the three POTRAs between *A. thaliana* OEP80 and *Nostoc/Thermosynechococcus* Omp85s are 34.3/33.3%, which are higher than that between *A. thaliana* Toc75 and cyanoOmp85 (28.5/27.6%) or *A. thaliana* Toc75 and *A. thaliana* OEP80 (22.4%). The POTRA domains have been implicated for binding with oligomeric complex partners in Gram-negative bacteria (Knowles et al., 2009). Thus, the high similarity between OEP80 and cyanoOmp85 in these domains suggests that these two proteins may have similar binding partners. The presence of conserved residues and the high overall sequence identity suggests that OEP80 may have retained the function of the protein in the ancestral cyanobacterial endosymbiont while Toc75 has taken on a new function in protein import.

For analysis of the C-terminal portion, in addition to the alignment of the primary sequences, we used the Phyre2 web server (Kelley and Sternberg, 2009) to thread sequences from *A. thaliana* Toc75 and OEP80 with that of *N. gonorrhoeae* BamA (NgBamA) whose structure has been determined by X-ray crystallography (Noinaj et al., 2013). The result allowed annotation of 16 transmembrane β-strands (TMβ1-16) connected by eight loops (eL1-8) and seven turns (T1-7) (Figure 2C). Among residues conserved in all three proteins are those in the motif Ser-Val-Arg-Gly-Tyr (SVRGY) in eL6, acidic residues in TMβ12 (Glu), and TMβ13 (Glu or Asp), and Tyr-Ala (YA) in TMβ15 [Figure 2C, shown in bold; the two motifs (SVRGY and YA) are also indicated by (iv,v), respectively]. The SVRGY motif and acidic residues in TMβ12 and 13 are also conserved in cyanoOmp85 but the YA motif is not (Figure 2D). Of the 26 cyanoOmp85 sequences examined in this study, only three have this motif and residues found in others are not as conserved in the case of the chloroplast homologs (Figure 2D, panel TMβ15). eL6 has been shown to interact with P5 and have dynamic positioning within the barrel of *E. coli* BamA (Rigel et al., 2013). It was also shown in *E. coli* BamA and TamA that, when in the barrel, eL6 was in a close proximity to TMβ15 and Arg in the SVRGY motif could form a salt bridge with acidic residues in TMβ12 and 13 (Gruss et al., 2013; Noinaj et al., 2013; Ni et al., 2014). Thus, the interaction of eL6 with the β-barrel interior may be conserved in cyanoOmp85, OEP80, and Toc75. Our analysis also revealed features specific to Toc75 that may reflect its unique function. In particular, T7 between TMβ14 and 15 appears to be four residues shorter in Toc75 than that in OEP80 and cyanoOmp85 (Figures 2C,D). The region apparently missing in Toc75 is flanked by highly conserved sequences in both sides (Figure 2D, panel T7). The N-terminal flanking region is highly conserved between cyanoOmp85, Toc75 and OEP80, while the C terminus flanking region contains an LG motif, which is conserved not only in the three groups, but also in BamA (Figure S1).

### Phylogenetic analyses of chloroplast Omp85 homologs

Because extant cyanobacteria form a monophyletic lineage, we expected that Toc75 and OEP80 evolved after the duplication of ancestral cyanoOmp85. Grouping of OEP80 and Toc75 in a clade nested within cyanoOmp85 sequences was previously reported but it had low support (66% neighbor-joining bootstrapping) (Gentle et al., 2004). However, this idea was not necessarily supported by other studies. For example, a closer relationship of OEP80 to cyanoOmp85 than to Toc75 was supported by the degree of sequence identity (Eckart et al., 2002) and a phylogenetic analysis (Baldwin et al., 2005). Inoue and Potter used amino acid sequences for a portion of the β-barrel domains, corresponding to residues 711–818 of *A. thaliana* Toc75, as well as encoding nucleotide sequences for phylogenetic analyses (Inoue and Potter, 2004). They included Omp85 homologs from chloroplasts, mitochondria, cyanobacteria, and other Gram-negative bacteria (Table S1). Using maximum parsimony and Bayesian inference, they found that OEP80, Toc75, and cyanoOmp85 sequences grouped together although the interior relationships of the three to one another were not resolved. Moslavac et al. also analyzed Omp85 sequences from a variety of taxa although the number of sequences was small (Moslavac et al., 2005) (Table S1). They used maximum likelihood to infer a phylogeny and found that Toc75, OEP80 and cyanoOmp85 form a clade but with weak support. Once again, the relationship between the three groups of proteins within this clade was unresolved. More recently, an analysis using sequences from wider taxa within cyanobacteria and archaeplastida provided strong support for the grouping of Toc75 and OEP80 nested within the cyanobacterial Omp85 clade (Topel et al., 2012). The authors performed a Bayesian analysis using an alignment of the portion corresponding to residues 651–818 of *A. thaliana* Toc75. Their ability to find support for an alternative topology was limited because they rooted the tree to the sequence from the cyanobacterium *Gloeobacter violaceus*, and no outgroup from non-photosynthetic organisms was included. Nonetheless, the obtained results were consistent with the hypothesis that Toc75 and OEP80 emerged as the result of a duplication of the cyanoOmp85 homolog that was present in the cyanobacterial endosymbiont.

In order to address the inconsistency among available results, we re-examined the phylogenetic relationships of Toc75, OEP80, and cyanoOmp85 using wider taxon selection than previous studies. We initially included well-studied Omp85 homologs from other taxa as outgroups, namely BamA, TamA, and TpsB from Gram-negative bacteria and Sam50/Tob55 from mitochondria.

**Figure 3:**
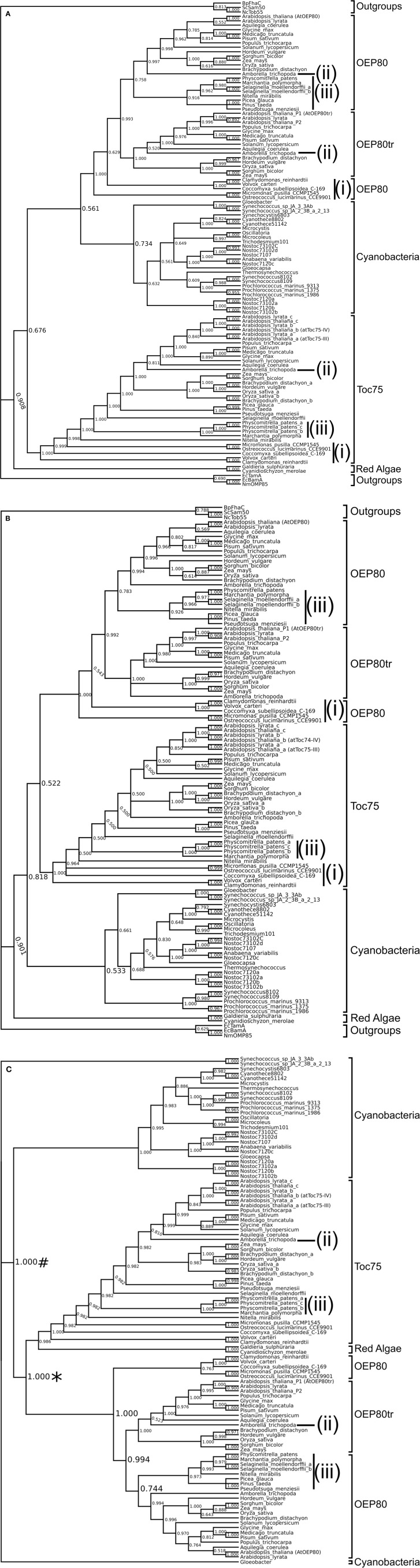
**Phylogenetic trees of Omp85 homologs**. Sequences of chloroplast Omp85 homologs (Toc75, OEP80, and OEP80tr), cyanoOmp85, and outgroups from other bacteria and mitochondria were aligned with MAFFT. Phylogenetic inference was done using Bayesian inference. Each tree shown is the 50% majority-rule consensus tree generated for the 3002 trees produced by two runs of two million generations each, sampled every 1000 generations with a 25% burn in. **(A)** Consensus tree using an alignment of amino acid sequences corresponding to residues 438 to 818 of *A. thaliana* Toc75 with poorly aligned regions removed by Gblocks. Numbers at nodes represent the proportion of trees in which a clade appeared, interpreted as the posterior probability (*PP*) of that clade. Discrepancies between our trees and the generally excepted plant species relationships are indicated as lower-case roman numerals (i–iii). **(B)** Consensus tree using an alignment of amino acid sequences corresponding to residues 648–818 of *A. thaliana* Toc75 with no interior section removed. **(C)** Consensus tree using an alignment of amino acid sequences excluding outgroup sequences corresponding to residues 438 to 818 of *A. thaliana* Toc75. The tree showed the chloroplast Omp85 homologs (^*^) nesting within cyanobacteria (#).

We first used the alignment of sequences corresponding to residues 438–818 of *A. thaliana* Toc75. This region includes P3-β3 and the entire transmembrane β-barrel, which was shown to be conserved well among various homologs (Reumann et al., 1999). To increase the reliability of the analysis, we removed ambiguously aligned regions using the Gblocks server (Castresana, 2000). Bayesian inference using this alignment provided strong support for a monophyletic relationship of the chloroplast Omp85 homologs and cyanoOmp85 (*PP* = 0.908). The analysis grouped OEP80 with cyanoOmp85 although the support for this grouping was low (*PP* = 0.561) (Figure 3A). The Toc75 orthologs from the green lineages, but not those from red algae, were placed sister to this clade although support for this relationship was also low (*PP* = 0.676). Similar to the previous report, we were able to identify only one chloroplast Omp85 homolog, which belongs to the Toc75 group, from each of the two Rhodophyta species (Topel et al., 2012) (Figure 3A). The tree topology was not entirely consistent with the established organismal classification. Within the Toc75 group, five sequences from the chlorophyte lineage were separated into three distinct groups [Figure 3A, indicated by (i)], and the sequence from the basal angiosperm *Amborella trichopoda* (Amborella Genome, 2013) was grouped with those from eudicots rather than being sister to the rest of the angiosperms [Figure 3A, indicated by (ii)]. In addition, sequences from the moss (*Physicomitrella patens*) and liverwort (*Marchantia polymorpha*) were grouped together as a clade sister to all vascular plant sequences [Figure 3A, indicated by (iii)]. In the case of the OEP80 clade, the location of the *A. trichopoda* sequence was consistent with the organismal classification. However, those of the algal sequences were not; instead they formed two groups. Interestingly, sequences from the moss, liverwort, a lycophyte (*Selaginella moellendorffii*), a streptophyte green alga (*Nitella mirabilis*) and three gymnosperms [white spruce (*Picea glauca*), loblolly pine (*Pinus taeda*), and Douglas fir (*Pseudotsuga menziesii*)] formed a clade sister to angiosperm non-truncated OEP80 sequences [Figure 3A, indicated by (iii)]. This result does not appear to follow the generally accepted relationships where (a) gymnosperms are grouped with angiosperms forming the seed plants, (b) lycophytes are grouped within vascular plants, (c) mosses are more closely related to vascular plants than to liverworts, and (d) land plants form a monophyletic group (Bowman, 2013). This may indicate large changes within the angiosperms and the absence of the species representing the intermediate state.

We wondered if the low support for the monophyletic relationship of OEP80 and Toc75 and inconsistency of the relationships between chloroplast Omp85 homologs with organismal classifications may be due to the choice of region for the sequence alignment. Thus, we used the portion of the sequence alignment corresponding to residues 648–818 of *A. thaliana* Toc75, which includes a C-terminal β-barrel covering TMβ9 and the C terminus and is almost identical to the region used in the previous study (651–818) (Topel et al., 2012). In order to be consistent with the previous study, we used the whole alignment without removing ambiguously-aligned regions. Similar to the first tree, the obtained tree supports a clear monophyletic grouping of Toc75, OEP80 and cyanoOmp85 (Figure 3B). In this analysis, Toc75 and OEP80 were placed in a clade sister to cyanobacterial homologs although the support was very low (*PP* = 0.522). Once again, cyanobacteria and the green lineage formed a clade excluding red algae, this time with higher support (0.818). The erroneous topology for OEP80 from green algae and non-flowering plants as well as Toc75 from green algae, *A. trichopoda*, moss and liverwort was consistent with the previous tree in Figure 3A [Figure 3B, indicated by (i–iii)].

Despite being based on an almost identical region as the previous study (Topel et al., 2012), our tree did not provide strong support for the grouping of Toc75 and OEP80 (Figure 3B). We wondered if this was due to the use of a different set of sequences: the previous work used sequences only from chloroplasts and cyanobacteria (Topel et al., 2012), while we included Omp85 homologs from mitochondria and bacteria other than cyanobacteria. In order to test this idea, we used sequences only from chloroplasts and cyanobacteria at the same region used in Figure 3A, removed ambiguously aligned segments, and generated a new tree (Figure 3C). The result showed strong support for a clade containing Toc75 and OEP80 [*PP* = 1.000, indicated with an asterisk (^*^)] nested within the cyanobacterial groups, similar to the previous result (Topel et al., 2012). The result suggests that the Omp85 homologs from mitochondria and bacteria other than cyanobacteria interfere with the internal relationships of homologs from cyanobacteria and chloroplasts. Interestingly, the placement of the chloroplast group in the current result was inconsistent with that in the previous work (Topel et al., 2012). The previous tree placed chloroplast homologs sister to those from *Oscillatoria sp.* PCC 6506 and *Microcoleus vaginatus*, while our analysis placed the chloroplast group sister to proteins from all cyanobacteria except for those from *Gloeobacter violaceus* and two *Synechococcus* species [Figure 3C, indicated with a number sign (#)]. Our result is consistent with the predicted origin of chloroplasts deep within the cyanobacteria phylogeny (Criscuolo and Gribaldo, 2011). The difference between the previous and current results may be due to the number of cyanobacterial species used for the analyses—the previous study used sequences from five species, while our study used sequences from 26 species (Table S1). Topologies within OEP80 and Toc75 except for those of chlorophyta OEP80 orthologs in Figure 3C were largely identical to those in Figure 3A, indicating that the presence of the Omp85 homologs from various bacteria and mitochondria does not affect the relationships within chloroplast orthologs. Unlike in the case of Figure 3A, the chlorophyte OEP80 orthologs are grouped together in Figure 3C with moderate support (*PP* = 0.763), which is consistent with organismal relationships. The red algal sequences are now grouped with Toc75 (Figure 3C).

In addition to OEP80 itself, the genomes of *A. thaliana* and several other angiosperms include its truncated forms such as Ath-P1 (named OEP80tr) and Ath-P2. Because we could find OEP80tr orthologs only in angiosperms (Figure 3 and Table S1), we hypothesized that the duplication leading to OEP80tr occurred after the divergence of angiosperms from extant gymnosperms. However, this hypothesis is not supported by any of the trees reported in this or the previous work (Topel et al., 2012). All of the available trees placed the OEP80tr clade sister to all the “full-length” OEP80 orthologs from streptophytes (Figure 3). The support for this topology was modest if not very low (*PP* = between 0.74 and 0.78) (Figure 3). This result may suggest several independent losses of OEP80tr during evolution of streptophytes. Alternatively, this may be due to relaxed selection or extensive subfunctionalization of OEP80tr in angiosperms which caused a change in sequence large enough to confound our analysis.

### Chloroplast targeting of Omp85 homologs

All known chloroplast Omp85 homologs are encoded in the nuclear genome. Toc75 is produced as a larger precursor with a cleavable bipartite targeting sequence in its N terminus. The first part is required for ATP-dependent import and removed by a stromal processing peptidase (Tranel and Keegstra, 1996). The second part contains a polyglycine stretch necessary for envelope sorting (Inoue and Keegstra, 2003) and is removed by envelope-located Plsp1 (Inoue et al., 2005; Shipman and Inoue, 2009; Midorikawa et al., 2014). Toc75-IV was shown to be targeted to chloroplasts *in vitro* without any change in mobility on SDS-PAGE and its membrane integration was independent of ATP (Baldwin et al., 2005). Similarly, an 80-kD protein consisting of residues 1–732 of OEP80 was targeted to the chloroplast membrane *in vitro* without changing the mobility on SDS-PAGE, and its targeting did not require ATP (Inoue and Potter, 2004). However, in the case of OEP80, a recent study demonstrated that the N-terminal portion corresponding to residues 1–52 does not exist in endogenous OEP80, which actually migrates around 70 kD on SDS-PAGE (Hsu et al., 2012). Currently the N-terminal sequence of OEP80 is unknown. Removal of the first 52 residues would yield a protein of 74 kD, leaving a possibility open that OEP80 contains a cleavable targeting sequence. In this scenario, the mechanism of envelope targeting of OEP80 should be distinct from that of Toc75 because OEP80 does not contain a polyglycine stretch. Finally, there has been no report of chloroplast targeting of OEP80tr orthologs.

**Figure 4:**
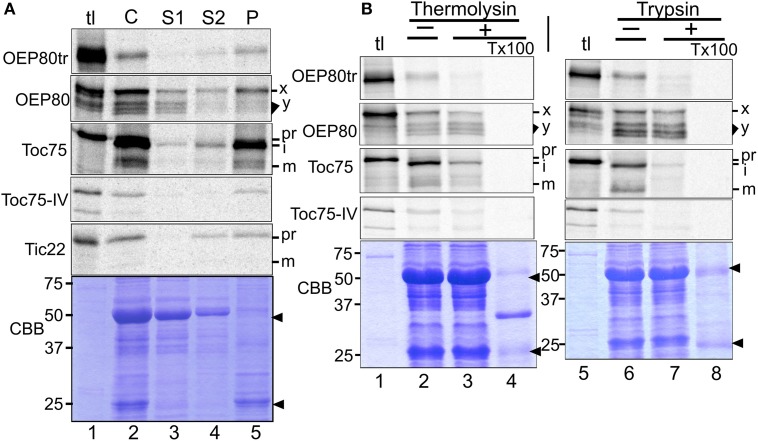
**Import of chloroplast Omp85 homologs *in vitro*. (A)** Chloroplasts isolated from pea seedlings were incubated with radiolabeled proteins indicated at left in the import condition for 10 min in the light. After the reaction, chloroplasts were re-isolated and divided into two samples. The first sample was loaded directly on SDS-PAGE (C). The second sample was lysed hypotonically and separated into supernatant (S1) and pellet fractions by centrifugation. The pellet fraction was further resuspended into 0.1M Na_2_CO_3_ and separated into the supernatant (S2) and pellet (P) fractions by another centrifugation. The obtained S1, S2, and P fractions contained soluble, peripheral membrane, and integral membrane proteins, respectively. Radiolabeled proteins recovered in each fraction were separated by SDS-PAGE and visualized by phosphorimager analysis. Each lane was loaded with the sample equal to chloroplasts containing 3 μg chlorophylls used for the import assay, and tl was loaded with 10% of the translation products equivalent to those used for the import assay containing 3 μg chlorophyll-equivalent chloroplasts. For OEP80, the 74-kD OEP80 precursor, and the 66–71 kD import products are indicated with x and y, respectively. For others, pr, i, and m indicate precursor, import intermediate, and mature forms, respectively. The Coomassie Brilliant Blue (CBB)-stained gel for OEP80tr is shown in the bottom. The major soluble protein of 50 kD (LSU = ribulose-1,5-bisphosphate carboxylase/oxygenase large subunit) and the major membrane protein of 25 kD (LHCP = light-harvesting chlorophyll a/b binding protein) are indicated with arrowheads. **(B)** Chloroplast isolated from pea seedlings were incubated with radiolabeled proteins indicated at left in the import condition for 30 min in the light. After the reaction, chloroplasts were re-isolated and treated for 30 min without (–) or with (+) thermolysin on ice or trypsin at room temperature. Digestion control was done by including detergent (Triton X-100 = TX100) in the reaction. After the reactions were quenched with EDTA (for thermolysin) or trypsin inhibitor (for trypsin), chloroplasts were re-isolated by a 40% Percoll cushion and the radiolabeled proteins in the resultant samples were examined as described in the legend to panel **(A)**. CBB-stained gels for OEP80tr are shown. See legend to panel **(A)** for descriptions of the labels.

We hypothesized that comparing targeting mechanisms of chloroplast Omp85 homologs may provide us with hints about their evolution. The immediate questions we had were as follows: Is OEP80tr targeted to the chloroplast outer membrane? Does the targeting of OEP80 and OEP80tr include processing and require ATP as in the case of Toc75? To test these, we conducted import assays using radiolabeled proteins synthesized by *in vitro* transcription and translation and chloroplasts isolated from pea seedlings. For OEP80, we used the DNA construct lacking the sequence encoding the first 52 residues for *in vitro* transcription because this portion is dispensable for proper expression and functioning of endogenous *OEP80* (Patel et al., 2008; Hsu et al., 2012). After the import reaction, intact chloroplasts were re-isolated and analyzed either directly (C) or after separation by lysis and centrifugation into soluble (S1), peripheral-membrane (S2), and integral-membrane (P) fractions. The fractionation was monitored by distribution of the abundant endogenous proteins located in the stroma [the large subunit of ribulose-1,5-bisphosphate carboxylase/oxygenase (LSU)] and thylakoids [light harvesting chlorophyll a/b binding protein (LHCP)] by Coomassie Brilliant Blue staining (Figure 4A, panel CBB), as well as the distribution of the newly-imported peripheral membrane protein Tic22 (Kouranov et al., 1998) (Figure 4A, panel Tic22). In order to test the location of the imported proteins, re-isolated chloroplasts were also treated with thermolysin, which has access only to the surface of the outer membrane (Cline et al., 1981), or trypsin, which can penetrate the outer but not the inner membrane of the chloroplast envelope (Jackson et al., 1998).

As shown in Figure 4A, all the Omp85 homologs except OEP80 were recovered mainly in the integral membrane fraction (lane 5). Imported Toc75 was degraded partially by thermolysin (Figure 4B, panel Toc75, compare lanes 2 and 3) and almost completely by trypsin (Figure 4B, panel Toc75, compare lanes 6 and 7). Under these conditions, both OEP80tr and Toc75-IV were largely degraded by both proteases (Figure 4B, panels OEP80tr and Toc75-IV, compare lanes 2 and 3, 6 and 7, respectively). The near-complete degradation of Toc75-IV by trypsin in our results appears to contradict the previous report showing its partial resistance to trypsin (Baldwin et al., 2005). This apparent inconsistency may be due to the low translation and import efficiency of Toc75-IV in our study (Figure 4B, panel Toc75-IV). Nonetheless, the obtained results support outer membrane localization of OEP80tr.

**Figure 5:**
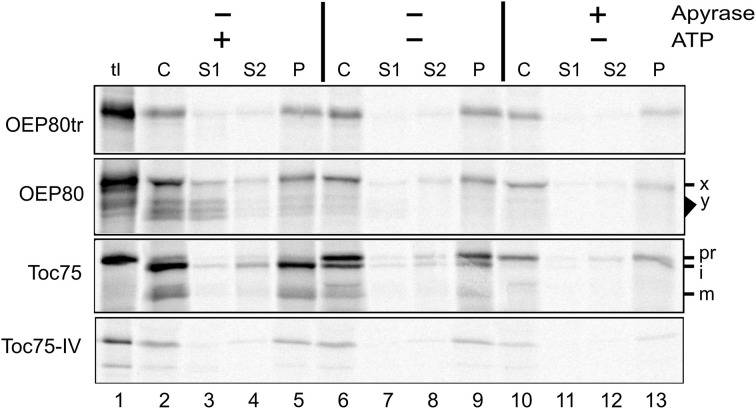
**Energy requirement for OEP80tr import *in vitro***. Translation products including the radiolabeled proteins indicated at left (tl) were incubated without (–) or with (+) apyrase at room temperature for 15 min in the light. The resultant samples were incubated with isolated chloroplasts without (–) or with (+) 3 mM MgATP for 30 min at room temperature in the dark. Chloroplasts were re-isolated, fractionated and examined as described in Figure 4A.

We also tested the ATP requirement for the import of chloroplast Omp85 homologs. Excluding ATP from the reaction disrupted import of Toc75 as evidenced by the increased level of the precursor (pr) and the decreased level of the intermediate (i) and mature form (m) (Figure 5, panel Toc75, compare lanes 2 and 6), but did not affect membrane integration of OEP80tr and Toc75-IV (Figure 5, panels OEP80tr and Toc75-IV, compare lanes 5 and 9). Interestingly, apyrase treatment disrupted import of Toc75 and Toc75-IV but did not affect that of OEP80tr (Figure 5, compare lanes 6 and 10; Figure S2). Again, our result with Toc75-IV appeared to contradict the conclusion of the previous study that chloroplast-association of Toc75-IV did not require ATP (Baldwin et al., 2005). This discrepancy may be due to the use of different methods to deplete nucleoside triphosphates (NTPs) in the reaction. The previous work used gel filtration to remove NTPs from the translation products (Baldwin et al., 2005). By contrast, our assay used apyrase which should remove NTPs not only from the translation products but also from the chloroplasts used for the assay. Thus, our data suggest that a slight amount of ATP is required for Toc75-IV targeting while it is not the case for the targeting of OEP80tr.

Quite interestingly, multiple bands were generated after import of OEP80 (Figure 4A, panel OEP80, lane 2). The band that migrated at the slowest rate corresponded to the 74-kD translation product (indicated as x) and was recovered in all the three fractions (S1, S2, and P), while bands in the area corresponding to 66–71 kD (indicated as y) were found predominantly in the soluble fraction (S1) but were also detected in the integral membrane fraction (P) (Figure 4A, panel OEP80, lanes 3–5). The 74-kD protein was partially digested by thermolysin and trypsin (the band x in Figure 4B, panel OEP80, compare lanes 2 and 3, 6 and 7, respectively). By contrast, the proteins around 66–71 kD were resistant to both proteases (the area y in Figure 4B, panel OEP80, compare lanes 2 and 3, 6 and 7, respectively). Appearance of all the bands was enhanced by the presence of ATP: especially, it appeared that ATP enhanced processing of the 74-kD protein to the smaller proteins around 66–71 kD (Figure 5, panel OEP80, compare lanes 2 and 6).

**Figure 6:**
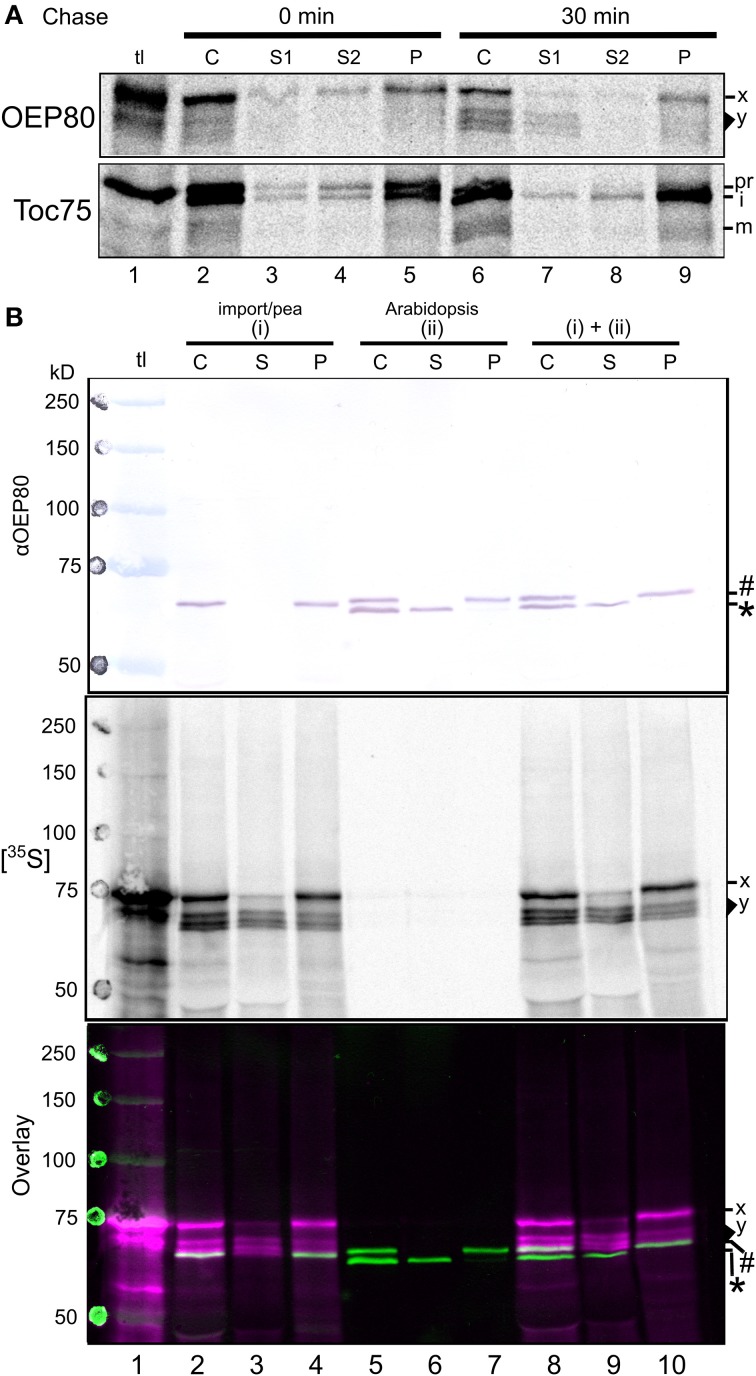
**Processing of OEP80 during chloroplast import *in vitro*. (A)** After 10-min incubation with radiolabeled precursors in the import condition in the dark, pea chloroplasts were re-isolated through a Percoll cushion and divided into two samples. The first sample was immediately lysed and separated into soluble (S1), peripheral membrane (S2), and integral membrane (P) fractions as described in the legend to Figure 4A. The second sample was resuspended in import buffer containing 3 mM MgATP for 30 min at room temperature in the light then separated into the three fractions. The OEP80 precursor of 74 kD and imported products ranging from 66–71 kD are labeled as x and y; precursor, intermediate and mature forms of Toc75 are indicated with pr, i, and m, respectively. **(B)** Intact chloroplasts isolated from pea seedlings and incubated with radiolabeled OEP80 in the import condition for 30 min in light (i) or chloroplasts isolated from *A. thaliana* seedlings (ii) were analyzed directly (C) or lysed hypotonically and separated into soluble (S) and pellet (P) fractions by centrifugation. Proteins in each fraction were separated by SDS-PAGE and transferred to a PVDF membrane. The membrane was incubated with the partially-purified anti-OEP80 antibody and the immunoreactions were visualized by colorimetric assay using alkaline phosphatase (αOEP80), and the radiolabeled proteins in the membrane were visualized by phosphorimager analysis ([^35^S]). The two images were overlaid using ink spots and radiolabeled marking spots (Overlay). In Overlay, radioactive signals are indicated with magenta and the immunoreactive signals with green. The lane tl was loaded with translation products and pre-stained (blue) molecular weight markers. Endogenous OEP80 of 70 kD and the soluble 63-kD protein of unknown identity in the *A. thaliana* chloroplasts recognized by the anti-OEP80 antibody are indicated with a number sign (#) and an asterisk (^*^); the OEP80 precursor of 74 kD and imported products ranging from 66–71 kD are labeled as x and y, respectively.

We wondered about the relevance of these findings. How are the bands of 66–71 kD produced from the 74-kD “precursor?” Do any of these smaller bands correspond to endogenous OEP80? As a first step to answer these questions, we conducted an *in vitro* import chase assay. After 10 min of the import reaction in the dark, intact chloroplasts that mainly contained the radiolabeled precursor were re-isolated, resuspended in import buffer containing ATP, and analyzed immediately or after further incubation for 30 min. The result showed a correlation between the decreased intensity of the 74-kD band mainly in the soluble and peripheral membrane fractions (S1 and S2) and the increased intensity of the 66–71-kD band mainly in the soluble fraction (S1) with a small amount in the integral membrane fraction (P) after the chase (Figure 6A, panel OEP80, compare lanes 2–5 and 6–9). Under the conditions used, a similar pattern was observed for Toc75: after the chase, the precursor (pr) decreased while the intermediate (i) remained roughly constant and the mature form (m) increased (Figure 6A, panel Toc75, compare lanes 2–5 and 6–9). Together, these results provide further support for the 66–71-kD bands being derived from the 74-kD protein of the OEP80 translation product.

To test if any of the 66–71 kD-proteins correspond to endogenous OEP80, the mobility of radiolabeled proteins produced by the import assay was compared with that of immunoreactive endogenous OEP80 in isolated chloroplasts on SDS-PAGE (Figure 6B). We used a partially purified antibody against recombinant OEP80 generated in the previous study (Hsu et al., 2012). As shown in Figure 6B, panel αOEP80, this antibody preparation did not recognize the 74-kD OEP80 translation product (lane 1) but cross-reacted with a single protein around 66 kD in pea chloroplasts (lane 2) and two proteins around 63 and 70 kD in *A. thaliana* chloroplasts [lane 5, indicated as an asterisk (^*^) and a number sign (#), respectively]. Lack of an immunoreactive band in the translation product (Figure 6B, lane 1) was attributed to the low amount of the protein in the sample which was nonetheless sufficient for detection by autoradiography (panel [^35^S]). The 66-kD immunoreactive protein in pea chloroplasts was considered to be the OEP80 ortholog because it was found only in the chloroplast membrane fraction (compare lanes 3 and 4 in Figure 6B, panel αOEP80) and its mobility appeared to correspond to what was reported in another study (Eckart et al., 2002). Among the two immunoreactive *A. thaliana* proteins, the 63-kD protein is a soluble protein of unknown identity (indicated with an asterisk ^*^ in Figure 6B, lane 6) and the 70-kDa membrane protein is endogenous OEP80 (indicated with a number sign # in Figure 6B, lane 7) as reported in the previous study (Hsu et al., 2012). Apparently, OEP80 orthologs from *A. thaliana* (70 kD) and pea (66 kD) migrated at different rates on SDS-PAGE when they were loaded separately, as clearly seen when the bands in neighboring lanes (pea OEP80 in lane 4 and *A. thaliana* OEP80 in lane 5) are compared in Figure 6B, panel αOEP80. Interestingly, however, the two orthologs co-migrated as a single band when the chloroplasts from *A. thaliana* and pea were loaded together (Figure 6B, panel αOEP80, lane 10). That is, the 66-kD pea OEP80 ortholog migrated around 70 kD together with *A. thaliana* OEP80. This may be due to the presence of endogenous proteins in *A. thaliana* chloroplasts other than OEP80 which migrated around 66 kD and affected the mobility of pea OEP80 and possibly that of *A. thaliana* OEP80. The smallest radiolabeled band from the import assay migrated around 66 kD when loaded with pea chloroplasts (included in the area y in lanes 2–4 in Figure 6B, panel [^35^S]). Notably, this protein appeared to migrate at the same rate as endogenous OEP80 in both pea and *A. thaliana* chloroplasts (Figure 6B, panel Overlay, white bands due to the overlap of the immunoreactive green bands and the radioactive magenta bands in lanes 2, 4, 8, and 10). Interestingly, this radiolabeled protein was found in both the soluble and membrane fractions (Figure 6B, panel [^35^S], lanes 3 and 4).

A previous study demonstrated that a C-terminal tag remains intact and does not disrupt the functionality of OEP80 *in vivo* (Hsu et al., 2012). Together, our results suggest that OEP80 may carry an N-terminal extension that is cleaved during import with the presence of ATP, and the processed form may be first released into the aqueous phase before being integrated into the membrane.

## Discussion

The chloroplast originated from a cyanobacterial endosymbiont. The initial phase of chloroplast evolution most likely involved duplication and transfer of genes from the endosymbiont chromosome to the host nuclear genome. Establishment of a protein import system at the outer and inner membranes of the endosymbiont must have been needed to allow replacement of gene copies in the endosymbiont with the duplicated copies in the host, leading to the conversion of the cyanobacterial endosymbiont to the organelle. Available data suggest that ancestral cyanoOmp85 evolved into two essential proteins in chloroplasts, i.e., the core component of the protein import channel, Toc75, and a protein of unknown function, OEP80. Furthermore, the *A. thaliana* genome encodes truncated forms for the chloroplast Omp85 homologs. Results of sequence alignments and structural predictions presented in this study suggest that OEP80 may have a function similar to that of cyanoOmp85. Comprehensive phylogenetic analyses provide support for the grouping of OEP80 and Toc75 as a clade sister to the one including most cyanoOmp85 homologs as long as the analysis used sequences only from cyanobacteria and chloroplasts and the trees were rooted within cyanobacteria. The obtained results also suggest extensive diversification of Omp85 homologs that may interfere with reconstructions of their phylogenetic relationships. Finally, results of our import assay suggest that both Toc75 and OEP80 are processed post-translationally and their import requires ATP, while the truncated forms are integrated into the chloroplast membrane without processing although the energy requirement of the integration appears to differ between Toc75-IV and OEP80tr. Together, the current data suggest that chloroplast Omp85 homologs have diversified in both function and targeting multiple times throughout the evolution of land plants.

Our results suggest that the unique occlusion of the β-barrel by eL6 shown in bacterial Omp85 structures is conserved in cyanoOmp85 and chloroplast Omp85 homologs. This appears to contradict the idea that Toc75 provided a pore for free transport of precursor proteins. It is possible that eL6 may not reach the pore, or its insertion may be dynamic and tightly regulated in Toc75. This idea may be tested by a combination of site-directed mutagenesis and a genetic complementation assay established previously (Shipman-Roston et al., 2010) and crystal structural analysis, which has never been done with chloroplastic Omp85 homologs. Results of these experiments should give valuable clues as to how the Omp85 homolog present in the ancestor of chloroplasts was converted to a chloroplast protein import channel, and how the import channel works.

Strong support for an OEP80-Toc75 clade nested in cyanobacteria was obtained only when distantly-related sequences were excluded. This may be explained by the extensive diversification of Omp85 because the outgroup sequences we used had very low sequence identity with the cyanobacteria and chloroplast sequences (between 14–27% in the region used for phylogenetic analysis). To some degree this justifies their exclusion. Also, the specification of *Gloeobacter* as the root of the cyanobacteria and chloroplast tree is reasonable so long as we assume that both Toc75 and OEP80 are of cyanobacterial origin. One argument against this approach, however, is that this specific assumption is one of the questions that were being tested. Even with the sequences from mitochondria and proteobacteria, our results showed that the chloroplast and cyanobacterial sequences form a clade. However, we cannot deny a possibility that either Toc75 or OEP80 entered plant genomes through horizontal gene transfer from a group of bacteria, which are closely related to but distinct from extant cyanobacteria.

The number of chloroplast Omp85 homologs in the model plant *A. thaliana* has increased as a result of duplications of Toc75 and OEP80 which gave rise to the homologs that lack POTRA domains during species diversification. Generation of Toc75-IV occurred recently, most likely shortly before the divergence of the Arabidopsis genus. By contrast, the duplication of OEP80 leading to OEP80tr homologs occurred at some point before the divergence of angiosperms or possibly that of land plants. A previous study showed a possible involvement of Toc75-IV in non-photosynthetic plastid development (Baldwin et al., 2005), while the function of OEP80tr remains unknown. The presence of OEP80tr in angiosperms, but not in other land plants, suggest that its biological role may be related to the function or the development of plastid types unique to angiosperms. Extensive analysis of publicly-available *OEP80tr* T-DNA insertion mutants should help address this question and ultimately establish the relevance of the duplication event for generating POTRA-less Omp85, which is unique to chloroplasts.

Results of the *in vitro* import assay suggest that OEP080tr was targeted to the outer membrane without a cleavable targeting sequence. Although the size and localization of the endogenous protein need to be tested using a specific antibody, the obtained result suggests that Toc75-IV and OEP80tr may use a similar mechanism for membrane targeting and integration although their energy requirement appears to differ. In addition, our results suggest that OEP80 may carry a cleavable targeting sequence in its N terminus. If so, it would be the second organelle outer membrane protein known to require a cleavable targeting signal. Available data suggest that Toc75 is never dissociated from the membrane, while our result suggests that OEP80 import may involve soluble intermediates. This could be due to the presence of the polyglycine stretch that may act as a membrane anchor in the case of Toc75. Also, if OEP80 goes through a soluble intermediate that is protected from proteases, it must be approaching the outer membrane from the inside of the chloroplast outer membrane. If the orientation of a β-barrel membrane protein is determined by the direction it approaches the membrane, then the orientation of OEP80 should be the same as bacterial Omp85 homologs, i.e., facing both N and C termini to the space between the outer and inner membranes. This hypothesis is at odds with a previous study, which showed that fluorescence from split GFP was seen at the edge of chloroplasts when a recombinant protein comprised of OEP80 with the 11th β-strand of GFP at its N terminus was co-expressed with cytosolically targeted GFP β-strands 1–10 (Sommer et al., 2011). They used an OEP80 construct that contained the N-terminal 52 amino acids that actually does not exist in the mature protein (Hsu et al., 2012). This extra section as well as the added portion of GFP may have prevented proper import of OEP80, thus leaving the entire protein in the cytosol. These ideas are still preliminary, and need to be tested by extensive *in vitro* and *in vivo* analyses.

## Author contributions

Philip M. Day and Kentaro Inoue designed the experiments. Philip M. Day conducted most of the experiments. Daniel Potter conducted phylogenetic analysis. Philip M. Day and Kentaro Inoue wrote the paper. All authors were involved in finalization of the paper.

### Conflict of interest statement

The authors declare that the research was conducted in the absence of any commercial or financial relationships that could be construed as a potential conflict of interest.

## Supplementary material

Table S1 **Sequences used for this study**.

Figure S1 **An alignment of sequences used for structure prediction and phylogenetic analysis**.

Figure S2 **Energy requirement for import of truncated chloroplast Omp85 homologs *in vitro***.
