# Supplementary material for: Evolution and targeting of Omp85 homologs in the chloroplast outer envelope membrane
Source: Front Plant Sci. 2014 Oct 13;5:535. doi: 10.3389/fpls.2014.00535 (PMC4195282; doi:10.3389/fpls.2014.00535)
Supplement: Figure S1 — An alignment of sequences used for structure prediction and phylogenetic analysis. [file DataSheet1.PDF]

Supplementary Figure 1

```
                                                   1
AtOEP80a                      .....................MH...............................................
AlOEP80a                      .....................MH...............................................
GmOEP80a                      .....................M................................................
MtOEP80a                      .....................M................................................
PsOEP80                       ......................................................................
SlOEP80a                      .....................M................................................
HvOEP80a                      .....................MG...............................................
SbOEP80a                      .....................MG...............................................
PtOEP80a                      .....................M................................................
AcOEP80a                      .....................M................................................
BdOEP80a                      .....................MG...............................................
OsOEP80a                      .....................MG...............................................
ZmOEP80a                      .....................MG...............................................
PpOEP80                       .................MC..LSEQSPGASP.......................................
SmOEP80a                      .....................MSSSRAEAPP.......................................
SmOEP80b                      .....................MSSSRAEAPP.......................................
CrOEP80                       ......................................................................
VcOEP80                       ......................................................................
MpOEP80                       .................M...HAESSASCSP.......................................
PgOEP80                       .....TGSSSLSQEG.AMT..MSEV.............................................
PtaOEP80                      .................MT..MAEV.............................................
PmOEP80                       .................MT..MAEV.............................................
AtOEP80b                      ......................................................................
AlOEP80b                      ......................................................................
AtOEP80c                      ......................................................................
GmOEP80b                      ......................................................................
PtOEP80b                      ......................................................................
MtOEP80b                      ......................................................................
psOEP80tr                     ......................................................................
SlOEP80b                      ......................................................................
BdOEP80b                      ......................................................................
SbOEP80b                      ......................................................................
ZmOEP80b                      ......................................................................
OsOEP80b                      ......................................................................
HvOEP80b                      ......................................................................
AcOEP80b                      ......................................................................
NmiOEP80                      .....................MASSGRSFSSSRGERERDEREDVEKGRVESRRALRQLDRDGAMAAVAFC
CsOEP80                       ......................................................................
MpuOEP80                      ......................................................................
OlOEP80                       ......................................................................
AtrOEP80a                     .....................MA...............................................
AtrOEP80b                     ......................................................................
Gloeobacter                   .....................MHP..............................................
Synechocystis6803             ..........MSNQNKSVI..LSP......................................YYRLLL..
Cyanothece8802                ........MDVTNEIKNLS..LSP......................................ILAILL..
Cyanothece51142               M....IGCVDVSNQSKKFH..FSP......................................LLGIIL..
Oscillatoria                  MW.....CVECPKIGIKMR..LSA......................................VLVAVF..
Microcoleus                   .................MR..LSA......................................VLVAVF..
Microcystis                   MEV.......LKNLNFSGR..YSS........................LA............LLLAIF..
Nostoc73102C                  .................MR..LSP......................................VLLAAI..
Anabaena_variabilis           .................MR..LSP......................................VLVAAV..
Trichodesmium101              .................MC..LYP......................................FLVAIF..
Thermosynechococcus           MNNELFSPVGMPSMG..SA..LKT......................................VGVAIA..
Synechococcus8102             .....................MTRRSTRRSP.........................VAVGQGLLGLAL..
Synechococcus8109             .....................MTRRSTRRSS.........................VAVRRTVLGLIL..
Nostoc7107                    .................MR..LSP......................................ILVAAV..
Gloeocapsa                    .................MR..ISP......................................FWAATV..
Nostoc7120a                   .................MR..V................................................
Nostoc7120b                   .................MR..ISS........................................AALL..
Nostoc7120c                   ................................................................MAAV..
Prochlorococcus_marinus_1375  .....................MDGFSTISSM.........................RLFRRGAYVLAL..
Prochlorococcus_marinus_1986  .................MRNNLSKFTKAFTS.........................MAYFSFIFI.....
Prochlorococcus_marinus_9313  .....................MASFLSSGPS.........................DALRRGAFGLVL..
Nostoc73102d                  .................MR..LSP......................................VLVAVL..
Nostoc73102b                  .................MR..VSA........................................TAIF..
Nostoc73102a                  .................MR...................................................
Synechococcus_sp._JA-3-3Ab    ......................................................................
Synechococcus_sp._JA-2-3B'a   .....................MSAAGR.................................QRVLTQAW..
alToc75c                      .................MPT.ISLNGQLIAA.........................PIVY.TTASSYLSS
alToc75a                      .................MAA.FSVNGQLI...........................PTTTSSTSSTSLSS
alToc75b                      ......................................................................
atToc75a                      .................MAA.FSVNGQLI...........................PTATSSTASTSLSS
psToc75                       .................MRT............................................S.....
acToc75                       ..................MA.FYAPGQLLRP.........................NICT....STSIPS
atToc75b                      ......................................................................
atToc75c                      ......................................................................
vcToc75                       .................MLG.RTATC............................................
smToc75                       ......................................................................
ppToc75a                      .................MAA.........................................CLPSTSTAC
ppToc75b                      ..............MLVLLF.VILMGAILRL.........................LVLT.ELGSDALGV
ppToc75c                      MD.....TVLHVSVSSPYST.IAAHRH.............................SSAKRLVPSSSTAC
zmToc75                       ..................MA.LASQGITLR........................................
bdToc75a                      ..................MA.LTSQSLFFS........................................
osToc75a                      ..................MA.LTSQSLFFS........................................
osToc75b                      ......................................................................
crToc75                       .................MQG.LKAT.............................................
bdToc75b                      .................MAP.FASG.............................................
GsToc75                       .................MQL.IENS...........................H........WIEFVSFAD
CmToc75                       MC..V............LGF.LAGDPLRVGA.....................HAL......KICTRSIAS
mpToc75                       .................MAG.LTYT.......................................STSLNT
pgToc75                       ......................................................................
ptaToc75                      .................MSS.ATLQ.................................CSLQLSNTGLPP
pmToc75                       .................MSS.APLQ.................................CSVQLSNTGLPP
atrToc75                      .................MAV.FTVNS..............................HVCTPSLPSLSLNP
MtToc75                       .................MSS............................................SSSF..
GmToc75                       .................MAS.FAAPN...................................NLGSAVLPS
ptToc75                       ..................MS.FTAST..............................NLVT....NAPLNT
slToc75                       .................MAS.IAAHGL.....................................AIPVNR
hvToc75                       ..................MA.LTSQSLFFT........................................
sbToc75                       ..................MA.LTSQGITLR........................................
NmiToc75                      ......................................................................
mpuToc75                      ..................MS.FAMTA......................................SASAAT
csToc75                       ......................................................................
olToc75                       ......................................................................
EcBamA                        .................MA..MKK......................................LLIASL..
EcTamA                        .................MRY...........................I............RQLCCVSL..
NmOMP85                       .................MK..LKQIAS..................................ALMMLGI..
BpFhaC                        .....................MTDATNRFRP..............................GLVGRAL..
ScSam50                       ......................................................................
NcTob55                       ...................................................................M..
```

**AtOEP80a**	....CHND
**AlOEP80a**	....DHHQD
**GmOEP80a**	....LRND
**MtOEP80a**	....PQND
**PsOEP80**	....
**SlOEP80a**	....HQNE
**HvOEP80a**	....PRRD
**SbOEP80a**	....RRPD
**PtOEP80a**	....IKND
**AcOEP80a**	....GRNE
**BdOEP80a**	....PRRD
**OsOEP80a**	....TRRD
**ZmOEP80a**	....CSRH
**PpOEP80**	....AKLA
**SmOEP80a**	....SALQ
**SmOEP80b**	....SALQ
**CrOEP80**	....
**VcOEP80**	....
**MpOEP80**	....RPRPR
**PgOEP80**	.....................................EGSL........................PVSSQ
**PtaOEP80**	.....................................EGSL........................PISSQ
**PmOEP80**	.....................................EGSL........................PISSQ
**AtOEP80b**	....
**AlOEP80b**	....
**AtOEP80c**	....
**GmOEP80b**	....
**PtOEP80b**	....
**MtOEP80b**	....
**psOEP80tr**	....
**SlOEP80b**	....
**BdOEP80b**	....
**SbOEP80b**	....
**ZmOEP80b**	....
**OsOEP80b**	....
**HvOEP80b**	....
**AcOEP80b**	....
**NmiOEP80**	DWVYAGREMLGRGQRELEKLAARWELALLKGDMSLRSEWSLKTWRTIRPSADGG.PVLRISGDGRRVTVN
**CsOEP80**	....
**MpuOEP80**	....
**OlOEP80**	....
**AtrOEP80a**	....ENQGN
**AtrOEP80b**	....
**Gloeobacter**	....LF....GRSGY....
**Synechocystis6803**	....LTSGL.VL....GASPAQATQ
**Cyanothece8802**	....GTAT..VF....SLAQSVRGE
**Cyanothece51142**	....TTTT..VW....VIDKPVRGQ
**Oscillatoria**	....TASA..TF....GLSNSANCQ
**Microcoleus**	....TASA..TF....GLSNSASGQ
**Microcystis**	..............................G........................LFH............
**Nostoc73102C**	....AITV..PL....GGSLSAKAE
**Anabaena_variabilis**	....AITA..PL....SSSLTANAQ
**Trichodesmium101**	....AALT..TF....GISKSANAQ
**Thermosynechococcus**	....TVAI..AL....GS.......
**Synechococcus8102**	....GLPLT................
**Synechococcus8109**	....GIPLV................
**Nostoc7107**	....AIAA..PL....SGSLKVNAQ
**Gloeocapsa**	....AIAA..PL....SLTVPAAGQ
**Nostoc7120a**	....
**Nostoc7120b**	....TL...TTLVASNATQ
**Nostoc7120c**	....AITA..PL....SSSLTANAQ
**Prochlorococcus_marinus_1375**	....AFPLG......GGAAK.....
**Prochlorococcus_marinus_1986**	....
**Prochlorococcus_marinus_9313**	....ALPLL................
**Nostoc73102d**	....AIAA..PL....GSCESANAQ
**Nostoc73102b**	....TL...ATLAAANITQ
**Nostoc73102a**	....
**Synechococcus_sp._JA-3-3Ab**	....MNR
**Synechococcus_sp._JA-2-3B'a**	....PGSAILLF....GVRGIPMNR
**alToc75c**	RRQY.............................YPL...........SSSREL........RFNASSSSR
**alToc75a**	RRKF....................................LSP....SSSRLP........RISSPS.PR
**alToc75b**	....
**atToc75a**	RRKF....................................LSP....SSSRLP........RISTQS.PR
**psToc75**	....VIPNRLTPTLTTHPSRRRNDHITTR
**acToc75**	....RRRKTP........QSNNNSSPF
**atToc75b**	....
**atToc75c**	....
**vcToc75**	....PGRLAA........RPTPRVASR
**smToc75**	....
**ppToc75a**	VV...GRRGLT........GGSSHS..Q
**ppToc75b**	NSAE.............................MVR.E.GLSI..RLCSSLSA........FATTEVVPK
**ppToc75c**	LV...SRRGSA........GASLQS..H
**zmToc75**	....SPPAGS........GG..HSGRR
**bdToc75a**	....PLPAGP........SR..RGRGR
**osToc75a**	....PLAAGP........SRRVRGRGR
**osToc75b**	....
**crToc75**	....PGLRASS......GRPTLRTART
**bdToc75b**	....
**GsToc75**	N...
**CmToc75**	R................................ERR..................................
**mpToc75**	RSSL....................................LHH..CADVFASS........DSAMQS.HA
**pgToc75**	....
**ptaToc75**	AIVF....................................NQP..RCKRTVAT........RATI.....
**pmToc75**	AILF....................................NQP..RCNRTVST........RATI.....
**atrToc75**	....NP..NPNRNRNP........KATVSGLNP
**MtToc75**	....FASNRLTTISTPPPPHRRNSHHISR
**GmToc75**	R...
**ptToc75**	TIF..SSRRKQ........RATV.....
**slToc75**	S..HRRLNS........ANTTTG...
**hvToc75**	....PSPAGP........SR..RGRGR
**sbToc75**	....SPPAGP........RG..HGGRR
**NmiToc75**	....
**mpuToc75**	GRAQ...PARMAA........GVRTKAASA
**csToc75**	....
**olToc75**	....
**EcBamA**	....
**EcTamA**	....L.............................
**NmOMP85**	....
**BpFhaC**	..................................VR..................................
**ScSam50**	....
**NcTob55**	....

```
                                10                                         20
                                 .                                          .
AtOEP80a                      DVRFSSSS................................IRIHSPS.......................
AlOEP80a                      DVRFSSSS................................IRIHSPS.......................
GmOEP80a                      DVRIVSSS................................IKIPLPS.......................
MtOEP80a                      DIRFISSS................................IKIPLPS.......................
PsOEP80                       ......................................................................
SlOEP80a                      DVRFTSSS................................IKLPQFT.......................
HvOEP80a                      GVRFVSSG................................VKLPRAC.......................
SbOEP80a                      .VRFVSSG................................VKLPSAP.......................
PtOEP80a                      DVSFTSSA................................LKIAPFL.......................
AcOEP80a                      DVRFVSSS................................IKLPSFP.......................
BdOEP80a                      .VRFISSA................................VKLPCAS.......................
OsOEP80a                      .VRFVSSG................................VKLPCAD.......................
ZmOEP80a                      .VRFVSSG................................VKLPSAS.......................
PpOEP80                       QARVSFSN................................VKLPPCK.......................
SmOEP80a                      PARILFSG................................IKVPDLSR......................
SmOEP80b                      PARILFSG................................IKVPDLSR......................
CrOEP80                       ...MMLRS................................YR.P..........................
VcOEP80                       .....MTS................................LRFP..........................
MpOEP80                       PARISFST................................VKLPVLE.......................
PgOEP80                       SVRFSWSS................................VKIPDCS.......................
PtaOEP80                      SVRFSWSS................................VKIPECS.......................
PmOEP80                       SVRFSWSS................................VKIPDGS.......................
AtOEP80b                      ......................................................................
AlOEP80b                      ......................................................................
AtOEP80c                      ......................................................................
GmOEP80b                      ......................................................................
PtOEP80b                      ......................................................................
MtOEP80b                      ......................................................................
psOEP80tr                     ......................................................................
SlOEP80b                      ......................................................................
BdOEP80b                      ......................................................................
SbOEP80b                      ......................................................................
ZmOEP80b                      ......................................................................
OsOEP80b                      ......................................................................
HvOEP80b                      ......................................................................
AcOEP80b                      ......................................................................
NmiOEP80                      HARLSYAR................................ISLPGCT.......................
CsOEP80                       ......................................................................
MpuOEP80                      ..MIASST................................ARRPSSS.......................
OlOEP80                       ......................................................................
AtrOEP80a                     PIRFISST................................IKIPTNA.......................
AtrOEP80b                     ......................................................................
Gloeobacter                   ....GQTA..............ASVL............................................
Synechocystis6803             AFSPLNMLAD.........GDNTELLV...............SPS.........................
Cyanothece8802                T.........................................MPP.........................
Cyanothece51142               N.........................................VPSVA.......................
Oscillatoria                  TSSSEPSE..............TGL.................TPS.SV.........KSSNLSELST...
Microcoleus                   TSYSESAE..............EALAA.V...........PPAPSIEL.........PDRPIPEL.....
Microcystis                   ......................................................................
Nostoc73102C                  TAKSSKQT..............TEVL................TLG.........................
Anabaena_variabilis           TPTNSEQT..............LEAI................APA.........................
Trichodesmium101              IFGSSVDT.DLVFISVNSKTQFAKLFS.T....KSFLDLGSSFPSQKLFVSRNNLLSKSWSEAEIKLDFD
Thermosynechococcus           .........................................LSPS.........................
Synechococcus8102             .........................................AAPV.........................
Synechococcus8109             .........................................APPV.........................
Nostoc7107                    TAEGLQQT..............AKVV................KPS.........................
Gloeocapsa                    TVDPSQIK..............SGSQ................PPQ.........................
Nostoc7120a                   ......................................................................
Nostoc7120b                   QAIAAPHT................................ASTPS.........................
Nostoc7120c                   TPTNSEQT..............LEAI................APA.........................
Prochlorococcus_marinus_1375  ......................AEVNW.............EKVPNSKI............NLGEVVWSNV
Prochlorococcus_marinus_1986  ......................................................................
Prochlorococcus_marinus_9313  .........................................AAPA.........................
Nostoc73102d                  TANSLKQT..............TKIF................TLG.........................
Nostoc73102b                  QATAASAK................................TVIPT.........................
Nostoc73102a                  ............................VQ........................................
Synechococcus_sp._JA-3-3Ab    SQQLGLRL..............ASVFA...........................................
Synechococcus_sp._JA-2-3B'a   SRQLRPWL..............TSALT...........................................
alToc75c                      FKSLTCSA....NLPTRKKIEPSSNNVLVKSLSKSFVFCSSAKPKD........................
alToc75a                      VPSIKC.................................SSSLPNR........................
alToc75b                      ......................................................................
atToc75a                      VPSIKC.................................SKSLPNR........................
psToc75                       TSSLKCH...............................LSPSSGDNN.......................
acToc75                       SPLISS......SSSSSSSSSPSSI.........QCAISSSSNPNTNN.........NPLNLNPITTA..
atToc75b                      ......................................................................
atToc75c                      ......................................................................
vcToc75                       PLSVRVQ.................................AAQPQVNQ......................
smToc75                       ......................................................................
ppToc75a                      NESFSALTSQ..QVGRTSRSRHTTV.........SAIAGTASESSS........................
ppToc75b                      PSTLVSCVATTGKARAVNNLSPVQG.....AVHTRKDVAAQFVPKKAR.........STRRQHSIVSAMA
ppToc75c                      NDVFSAFAPQ..QAEKLSRSRHTPI.........SSTAVSAFEPSP........................
zmToc75                       SSSVPAA.....A.APRGWGQPHAG.........QALSISPARYEP........................
bdToc75a                      AVTMSAA.....A...SHNSQPRNH..........PFAVSSSTPKS........................
osToc75a                      STSVSAA.....ASASSHNSQPHGH.......PQQPLAVASSSSKS........................
osToc75b                      ......................................................................
crToc75                       ALVVRAH.................................ASQPSSSN......................
bdToc75b                      ......................................................................
GsToc75                       ......................................................................
CmToc75                       ........................................RNAPRKQF......................
mpToc75                       QPGVRVM.........................NMHSLRGSASGPRR........................
pgToc75                       ......................................................................
ptaToc75                      ....RA................................VSSSSHN..............SVESG......
pmToc75                       ....RA................................VSSSSHD..............SVYSG......
atrToc75                      NPNLRATVCNGVSTSKVNLSSNNAFDPF.KWAKQKSSTVSASNPNPNL.........RATVSNGVSTSKV
MtToc75                       PSSVKCH...............................LSSSDDNNN.......................
GmToc75                       PPPVKCT.....LFPSCNNNNNHSL...........SNPSPSHPPP........................
ptToc75                       NP..RA......SCIKCDIISPPSF.............PSPQNPKP........................
slToc75                       ............TSVTAASLHPLTI......KSKLPSFSSSQNPKPS.......................
hvToc75                       PVTMSAA.....A...SHNSQPRNN..........PFAVSSSAPKS........................
sbToc75                       SSSVPAA.....A.VPRGWGQHHAG.........QALSISPARYEP........................
NmiToc75                      ......................................................................
mpuToc75                      VARLSASKIDA..................KRGGVAATAVASGSPRL........................
csToc75                       ......................................................................
olToc75                       ......................................................................
EcBamA                        ......................................................................
EcTamA                        ......................................................................
NmOMP85                       ......................................................................
BpFhaC                        ......................................................................
ScSam50                       ......................................................................
NcTob55                       ........................................ASSPSA........................
```

```
AtOEP80a                            ..PKEQHS..............................................................
AlOEP80a                            ..SKE.HS..............................................................
GmOEP80a                            ..ISK.HP..............................................................
MtOEP80a                            ..SKP.KP..............................................................
PsOEP80                             ......................................................................
SlOEP80a                            ..PLTLHHHT..................................................LNPFF.....
HvOEP80a                            ......................................................................
SbOEP80a                            ......................................................................
PtOEP80a                            ..HHQTKP..............................................................
AcOEP80a                            ..STN.HP..............................................................
BdOEP80a                            ......................................................................
OsOEP80a                            ......................................................................
ZmOEP80a                            ......................................................................
PpOEP80                             ..............................................................PRR.....
SmOEP80a                            ..QFQQHNA....................................................IPSL.....
SmOEP80b                            ..QFQQHNA....................................................IPSL.....
CrOEP80                             ......................................................................
VcOEP80                             ......................................................................
MpOEP80                             ..............................................................PRP.....
PgOEP80                             ..IER.HP..............................................................
PtaOEP80                            ..VER.HP..............................................................
PmOEP80                             ..IQR.HP..............................................................
AtOEP80b                            ......................................................................
AlOEP80b                            ......................................................................
AtOEP80c                            ......................................................................
GmOEP80b                            ......................................................................
PtOEP80b                            ......................................................................
MtOEP80b                            ......................................................................
psOEP80tr                           ......................................................................
SlOEP80b                            ......................................................................
BdOEP80b                            ......................................................................
SbOEP80b                            ......................................................................
ZmOEP80b                            ......................................................................
OsOEP80b                            ......................................................................
HvOEP80b                            ......................................................................
AcOEP80b                            ......................................................................
NmiOEP80                            ....EVSSG....................................................ACSR.....
CsOEP80                             ......................................................................
MpuOEP80                            ......................................................................
OlOEP80                             ......................................................................
AtrOEP80a                           ..LYTENNQ....................................................SPVP.....
AtrOEP80b                           ......................................................................
Gloeobacter                         .............................................LVAQL....................
Synechocystis6803                   ...SELNFPGPTSE......SEASNY......STLQEAA....PSVINAL..NGENGEISVIP.PEIEQI
Cyanothece8802                      .....ENEAA......................TSLELN.....NEV........................
Cyanothece51142                     ...SIQDPET......................SSLEFL.....DSLPSES..WGNDSSKKVDPYPKVSAI
Oscillatoria                        ...STQKPGEPIS........KNATK......KSLQAHRY...PTFLENFGVTGKAESTEIFLLP.....
Microcoleus                         ....TQNP..PQN........RKAAK..........................TGKGEALTIAVAP.....
Microcystis                         ...........................................GTVMAEE....................
Nostoc73102C                        ...TNQQPEKDTS........QLDSS......KNLKSRSNLT..GIIEEQ....KSRIVAVYP.......
Anabaena_variabilis                 ...TNQQSEQNV.....................SLESPEDFTTVKALADM....QSPSGELST.......
Trichodesmium101                    NLLKIKEITKSSS.I...YKKELANFWDLYVLENLEN......PQLILTN.KSTKTKSEFLVN.......
Thermosynechococcus                 ...RGQEA..............................................................
Synechococcus8102                   ...LAQ...................D......TPV..................VEVEQVEVAQ.......
Synechococcus8109                   ...MAQ...............EAMPE......NQA..................AESKEVVVAP.......
Nostoc7107                          ...TSKPQEKDAG........QNSAR......ESLQSAINSTEAKVLEDT....QSQSVKILP.......
Gloeocapsa                          ...MAQEPLPAT................................GVVVET....KTEQRQL.........
Nostoc7120a                         ......................................................................
Nostoc7120b                         ......................................................................
Nostoc7120c                         ...TNQQPEQDV.....................RSESPEDFTTVKALADM....QSPSVEFST.......
Prochlorococcus_marinus_1375        DNGKTQREIAKSN.IKEDSVEKISQD....KRTQENL......DEIVESNIKEDSVEKISQDK.......
Prochlorococcus_marinus_1986        .......................SNN......SELAA......RDLLYKE...GEFKKIKINN.......
Prochlorococcus_marinus_9313        ...RAQ...............TPEQD......TQPPA......AEILVN..DDVQVEEVATPE.......
Nostoc73102d                        ...TNQQAEKGTA........QSHFS......QGLEFHPNTI..GIPNSE..YLKSQSVKALT.......
Nostoc73102b                        ......................................................................
Nostoc73102a                        ......................................................................
Synechococcus_sp._JA-3-3Ab          ...........................................CGVVLRT....................
Synechococcus_sp._JA-2-3B’a         ...........................................RGMVLCT....................
alToc75c                            .......................SSN......CNLR..................................
alToc75a                            ...DTET................SSK......DSLL..................................
alToc75b                            ......................................................................
atToc75a                            ...DTET................SSK......DSLL..................................
psToc75                             ...DSFN.........................SSLL..................................
acToc75                             ...TTND................NNN......KSFT..................................
atToc75b                            ......................................................................
atToc75c                            ......................................................................
vcToc75                             ....TDQ.............ECSSSSN....CSGSTF.................................
smToc75                             ......................................................................
ppToc75a                            ..EDSQV.............NIREGG......ESLDW.................................
ppToc75b                            D.FDSDSPTENFG......P...SSE......SDVNW.................................
ppToc75c                            ..GDSQG.............NLGEDG......ESLDW.................................
zmToc75                             ...AARPP..............................................................
bdToc75a                            ...ESKTS..............................................................
osToc75a                            ...ESKGS..............................................................
osToc75b                            ......................................................................
crToc75                             ...HAEQQ............ECSSSG......SGVSV.................................
bdToc75b                            ...........Y..........................................................
GsToc75                             ...............................TSHVQLDC...............................
CmToc75                             ...PADRTGQRWH..................RSTTWL.................................
mpToc75                             ....................QRASSQ......NSLL..................................
pgToc75                             ......................................................................
ptaToc75                            ...HGNDGFEPI........RWGSKN......SRHV..................................
pmToc75                             ...HGNDGFEPI........RWDSKN......SRNI..................................
atrToc75                            N.LSSNNAFDPF........KWAKQK......SSII..................................
MtToc75                             ...NNND.........................SSSLF.................................
GmToc75                             ..................................LF..................................
ptToc75                             ...HSNS.............KQQKRQ......NSLL..................................
slToc75                             ...SSNT................RDN......SNIF..................................
hvToc75                             ...ESKTS..............................................................
sbToc75                             ...AARPP..............................................................
NmiToc75                            ......................................................................
mpuToc75                            ....................QPRAEI......SSID..................................
csToc75                             ...DEEEDDEEY........EEGDEE............................................
olToc75                             ......................................................................
EcBamA                              ......................................................................
EcTamA                              ......................................................................
NmOMP85                             ......................................................................
BpFhaC                              ......................................................................
ScSam50                             ......................................................................
NcTob55                             ......................................................................
```

```
                                                                             30
                                                                              .
AtOEP80a                       ........................................LL.TNL......................QS
AlOEP80a                       ........................................LL.TNL......................KS
GmOEP80a                       ............................................TC......................PL
MtOEP80a                       ............................................TS......................PF
PsOEP80                        ......................................................................
SlOEP80a                       ..........TN.........................LHLIL.QNF......................PK
HvOEP80a                       ....................................................................PA
SbOEP80a                       ...........................................ASA......................PS
PtOEP80a                       ............................................SL......................PF
AcOEP80a                       .......................................FVF.SNL......................PF
BdOEP80a                       ............................................PA......................PD
OsOEP80a                       ............................................AA......................PA
ZmOEP80a                       ...........................................APS......................PF
PpOEP80                        .................................PATRLQILP............................
SmOEP80a                       .................................LDRINSPLA.AAR......................SL
SmOEP80b                       .................................LDRINSPLA.AAR......................SL
CrOEP80                        ....................................................................PM
VcOEP80                        ....................................................................PS
MpOEP80                        .................................PVSSSPYLA.SSS......................SF
PgOEP80                        .....................................PRFNL.AQF......................PL
PtaOEP80                       .....................................PRFNL.SQF......................PL
PmOEP80                        .....................................TRFNL.AQF......................PL
AtOEP80b                       ......................................................................
AlOEP80b                       ......................................................................
AtOEP80c                       ......................................................................
GmOEP80b                       ......................................................................
PtOEP80b                       ......................................................................
MtOEP80b                       ......................................................................
psOEP80tr                      ......................................................................
SlOEP80b                       ......................................................................
BdOEP80b                       ......................................................................
SbOEP80b                       ......................................................................
ZmOEP80b                       ......................................................................
OsOEP80b                       ......................................................................
HvOEP80b                       ......................................................................
AcOEP80b                       ......................................................................
NmiOEP80                       ..........MNNA.......................RGDVS.AAT......................CR
CsOEP80                        ......................................................................
MpuOEP80                       ....................................................................PF
OlOEP80                        ......................................................................
AtrOEP80a                      ..........LQGSKPSLMVENRACPVRFTSSSIKIPEFFLG.PKT......................PF
AtrOEP80b                      ......................................................................
Gloeobacter                    .....GFT............................................................PA
Synechocystis6803              DGDRLGQTMEISAGNLDVGVDDLPPMAPVDSAELAQANELPH.GNA.......VAQ.......VA...PR
Cyanothece8802                 ....................................ANDLILRSDA.......QLELE....DLN...PS
Cyanothece51142                K........EAISSSEKSTVSSQPS...IEQQAAIESNDVII.SDP.......WLDESSDTVDPY...PK
Oscillatoria                   ..........KNPSKQENTIG...........WIEETKTAIS.RDLGKISESNQPHNIAQIVELKSCSPL
Microcoleus                    ..........ARGENDRKPPD...........RFTDTPSPVS.PSL...............LQLK...PE
Microcystis                    ...................................EPQEMVF..........................PR
Nostoc73102C                   ..........ANPAAMPAVVTERSRSA...GGHAYATPGVIV.PT..........STTLKTAQQT...PQ
Anabaena_variabilis            ..........DKSTKSAAT................TKKDVIV.PTV.........ETLVAT...N...PP
Trichodesmium101               ....KQFS..VSISNLE...........MFNNRNQNFQKLVLSKSTETKS..E...............PL
Thermosynechococcus            ....................................................................PT
Synechococcus8102              ..................................L...DALE............................
Synechococcus8109              ..................................TVGEDAL.............................
Nostoc7107                     ..........MQSD.....................ARSQVMV.PTIAQ.....APETIPNTDTQQ...PN
Gloeocapsa                     ...........NSAKL...................LGQTPVT.PT...................T...PQ
Nostoc7120a                    ......................................................................
Nostoc7120b                    ..................................KQNENLVI..........................PV
Nostoc7120c                    ..........DKSTKSAAT................TKKDVIV.PTL.........ETLVAT...K...PP
Prochlorococcus_marinus_1375   ..................................KTEEDIVEITESSDKD..................DS
Prochlorococcus_marinus_1986   ..................................KNTNQYLN..........................NR
Prochlorococcus_marinus_9313   ..................................GVAEESLG............................
Nostoc73102d                   ..........VNSASL...................ASPEVVV.PT..........STMAQTLQRN...PN
Nostoc73102b                   ..................................AKAGNLVV..........................PI
Nostoc73102a                   ......................................................................
Synechococcus_sp._JA-3-3Ab     .....AFA............................................................NG
Synechococcus_sp._JA-2-3B'a    .....AFAF.AEASR.....................................................SA
alToc75c                       .....................................KP.LAISL.................VVTSVSSA
alToc75a                       .....................................KN.LAKPL.................AVASVSSA
alToc75b                       ......................................................................
atToc75a                       .....................................KN.LAKPL.................AVASVSSA
psToc75                        .....................................KT.....IS................TTVAVSSA
acToc75                        .....................................KN.LA........................LSAA
atToc75b                       ......................................................................
atToc75c                       ......................................................................
vcToc75                        .....................................SQPAPIGRL................SIFGIPSA
smToc75                        ......................................................................
ppToc75a                       .....................................KQ.AAL......................VATGV
ppToc75b                       .....................................RQ.AAL...................VTTGVGAA
ppToc75c                       .....................................RQ.AAL......................VVAGA
zmToc75                        .....................................WR.GSLA....................AAIRAA
bdToc75a                       .....................................KTTLALG....................SAIAAA
osToc75a                       .....................................KT.FALA....................SAITAA
osToc75b                       ......................................................................
crToc75                        .....................................NQPSALGRL................GKFGLSSA
bdToc75b                       .....................................RTALA......................SAISAA
GsToc75                        ..................................YRRRDKLVCKQP......................QN
CmToc75                        ..................................SKRGEPLTCSWQ.....................SQM
mpToc75                        .....................................SS.FPWR.....................KVASC
pgToc75                        ......................................................................
ptaToc75                       .....................................KA.SL........................GVGI
pmToc75                        .....................................GA.SL........................GAGI
atrToc75                       .....................................GK.IL........................GISA
MtToc75                        ....................................RKT.....LT................ATVAISS.
GmToc75                        .....................................KA.IAI.........................SS
ptToc75                        .....................................KA.LAF......................SGTAA
slToc75                        .....................................AS.KQLQ..................NVVKTSAA
hvToc75                        .....................................KTDLALG....................SAIAAA
sbToc75                        .....................................RG.GSSS....................SAIRAA
NmiToc75                       ......................................E...............................
mpuToc75                       .....................................RS........................KRGVGGA
csToc75                        .....................................EE.......................EEEGEEAA
olToc75                        ......................................................................
EcBamA                         ......................................................................
EcTamA                         ......................................................................
NmOMP85                        ......................................................................
BpFhaC                         ......................................................................
ScSam50                        ......................................................................
NcTob55                        ......................................................................
```

```
                                            40        50                                      60
AtOEP80a                      CSKTF..........VSHLSNTRNSL.......................NQMLQ.....SLKNRHTPPPR
AlOEP80a                      CSKTF..........VSQLCNTRLSL.......................TQMLE.....SLRNRHT.PPR
GmOEP80a                      ..RTA..........HSHIANATNSI.......................AQLIN.....SFTSHSA....
MtOEP80a                      ..KTL..........HSHFTNATNSF.......................SHLIH.....SFTTHST....
PsOEP80                       ......................................................................
SlOEP80a                      FQHPF..........HRNGG.ISQNL.......................SKFTH.....PFHQKFN....
HvOEP80a                      ......................................................................
SbOEP80a                      ......................................................................
PtOEP80a                      FSQFV..........QTKLT...............................FLD.....SLLTRTR....
AcOEP80a                      CSQTL..........TSNLIKTRESI.......................TNFIS.....SITNRGK....
BdOEP80a                      ......................................................................
OsOEP80a                      ......................................................................
ZmOEP80a                      ......................................................................
PpOEP80                       ..............NQSPLGNFVSAA.......................EGLIQ.....SFCTKWV....
SmOEP80a                      LQRVL............PPRAATSGR.......................EPLLQ.....SLQAR......
SmOEP80b                      FQRVL............PPRAATSGR.......................EPLLQ.....SLQAR......
CrOEP80                       LAAIT.................................................................
VcOEP80                       FPDL..................................................................
MpOEP80                       FSRVSRK..KKNDESQNPVQNVVRSA.......................ENLLQ.....NAWARWG....
PgOEP80                       ..RAF..........ASNLEKARDFA.......................KGFIG.....SVVHGNR....
PtaOEP80                      ..RAF..........ASNLEKARNFA.......................KGFIG.....SVVHGNR....
PmOEP80                       ..RAF..........ASNLEKARDFA.......................KGFIG.....SVVHGNR....
AtOEP80b                      ......................................................................
AlOEP80b                      ......................................................................
AtOEP80c                      ......................................................................
GmOEP80b                      ......................................................................
PtOEP80b                      ......................................................................
MtOEP80b                      ......................................................................
psOEP80tr                     ......................................................................
SlOEP80b                      ......................................................................
BdOEP80b                      ......................................................................
SbOEP80b                      ......................................................................
ZmOEP80b                      ......................................................................
OsOEP80b                      ......................................................................
HvOEP80b                      ......................................................................
AcOEP80b                      ......................................................................
NmiOEP80                      SQSLLMK..KRR...........GAP.......................GAILK.....LIGAHWA....
CsOEP80                       ......................................................................
MpuOEP80                      ASVTT.........ASSPASTA................................................
OlOEP80                       ......................................................................
AtrOEP80a                     FTNT...................EKP.......................NEFLH.....KLNNSLTKPPK
AtrOEP80b                     ......................................................................
Gloeobacter                   LLAQEQA...............................................................
Synechocystis6803             VAQVEQ....ENLIAETKTDNQTDHV...EPQSPL.........................MAQAAVEEE.
Cyanothece8802                VS..............................................................SLKKSI
Cyanothece51142               VSTIQETKPIPENLPNSPTAISQEQT...ELKTSEALT................H...KSLGQTSLDKAI
Oscillatoria                  ENQQPD........RDSTTNKVMGRTSC....PPDQKLAKN........GETYQFKQAETISERALGNP.
Microcoleus                   LASNPS........PASSSQNALPEAQ......QDSSLA.............................P.
Microcystis                   IEQIVQ................................................................
Nostoc73102C                  INP.............SPTQQPSPAP...DIPPP............................EIQQPTP.
Anabaena_variabilis           MQE............SNSASKIAPV....EIGKPTSSAVFRSQYQTINYGNYGR.....SLTSGVSSSP.
Trichodesmium101              INKQFTATIPQGKIVENNSQNSQNVVLSKSTETKSESLVNKQFIANIPQGKIVEK...ESLDSQNMVLSK
Thermosynechococcus           LSQN..................................................................
Synechococcus8102             VEA...................................................................
Synechococcus8109             VEQ...................................................................
Nostoc7107                    TVPD..........SSSPATQPTPEP..ENTNPPTT...IEPAFPTTP.GN..........TQPENTTP.
Gloeocapsa                    PQPSPVFPSPIQGVPATPPQQPVPV.....TPPPGA.............GG...........APQDETP.
Nostoc7120a                   ......................................................................
Nostoc7120b                   VEETPVQL..............................................................
Nostoc7120c                   IQE............SNPTTKIASV....EIGKPTSTAVFRSQYQTINYGSYGR.....SLTSGVSASP.
Prochlorococcus_marinus_1375  VEKEAEAIR.............................................................
Prochlorococcus_marinus_1986  ISAFNFA...............................................................
Prochlorococcus_marinus_9313  IEQIQVT...............................................................
Nostoc73102d                  LT..............................................................QQQTP.
Nostoc73102b                  IEDTPTRI..............................................................
Nostoc73102a                  ......................................................................
Synechococcus_sp._JA-3-3Ab    LSMRS.................................................................
Synechococcus_sp._JA-2-3B'a   LLMGS.................................................................
alToc75c                      AA.......................LFL.....................PRLFSL...LSIV........
alToc75a                      AS...............................................FFLFRI...SNL.........
alToc75b                      ......................................................................
atToc75a                      AS...............................................FFLFRI...SNL.........
psToc75                       AA...............................................SAFFLT...GSLH........
acToc75                       AA...............................................GVLFHL...SSSV........
atToc75b                      ......................................................................
atToc75c                      ......................................................................
vcToc75                       LS....................................................................
smToc75                       ......................................................................
ppToc75a                      VA...............................................GL...................
ppToc75b                      VT....................................................................
ppToc75c                      VA...............................................GL...................
zmToc75                       ASAGAHHGSD..PVPAEPRIEL.PAIFTVFSEAAKT............GAAFFIA...SSSAA.......
bdToc75a                      AS...............................................GAFLLA...SS..........
osToc75a                      AS...............................................GAFLLA...SSG.........
osToc75b                      ......................................................................
crToc75                       LS....................................................................
bdToc75b                      AASDSRDPPP.......PR....PVVPT..........................................
GsToc75                       VSSQQRRK...HRIISSFQQTRNIYSQC..........................................
CmToc75                       LDSMARSRRKRHRNTTKPGVKR...SWC..........................................
mpToc75                       VA...............................................GVASGF...SSV.........
pgToc75                       ......................................................................
ptaToc75                      AA...............................................SIVFQL...............
pmToc75                       AA...............................................SIVFQL...............
atrToc75                      AA...............................................GLVCHI...FAC.........
MtToc75                       AA...............................................SALFLT...DSL.........
GmToc75                       AA...............................................SILMHC...TPLSP.......
ptToc75                       VA...............................................TIFLRF...TPV.........
slToc75                       IG...............................................AGFYAL...SSGV........
hvToc75                       AS...............................................GAFLLA...SS..........
sbToc75                       ASAGAQPGSD..PVPAEPRIEL.PAIFTVFSEAAKT............GAAFFIA...SSGAA.......
NmiToc75                      ......................................................................
mpuToc75                      LAKATK...........PL....KALAC.....................GIIGVT...TAFT........
csToc75                       EAQKEEDE..............................................................
olToc75                       ......................................................................
EcBamA                        ......................................................................
EcTamA                        ......................................................................
NmOMP85                       ......................................................................
BpFhaC                        ......................................................................
ScSam50                       ......................................................................
NcTob55                       ......................................................................
```

```
                                70                             80        90          100
AtOEP80a                      SVRRPNLPT.....................QMLNSVTQLMIGKSSPISL..SLIQS..TQFNWSE.SRD.
AlOEP80a                      SVRRPNLPT.....................QMLNSVTQLMIGKSSPISL..SLIQS..TQLNWSSGSGD.
GmOEP80a                      ..................................ELTRSVIQKSS.........................
MtOEP80a                      ..................................QLTRSVLQKSH.........................
PsOEP80                       ......................................................................
SlOEP80a                      .......PQ.........................NAILQFLSKPRNIN...........PFSWSL.....
HvOEP80a                      .......PA.............................................................
SbOEP80a                      .......PA.............................................................
PtOEP80a                      ...FPNSP.......................LLCSASLSLTRPSSP....................GPD.
AcOEP80a                      ..................................N.EKFFTSKSS.........................
BdOEP80a                      .......PA.............................................................
OsOEP80a                      .......PA.............................................................
ZmOEP80a                      .......PA.............................................................
PpOEP80                       .......PI.......................AIRARE................................
SmOEP80a                      ......................................................................
SmOEP80b                      ......................................................................
CrOEP80                       ...............................MQGPVAV................................
VcOEP80                       ......................................................................
MpOEP80                       .......PD.......................EKKVEEFF..............................
PgOEP80                       ..........................................................NALH........
PtaOEP80                      ..........................................................NALN........
PmOEP80                       ..........................................................NALQ........
AtOEP80b                      ......................................................................
AlOEP80b                      ......................................................................
AtOEP80c                      ......................................................................
GmOEP80b                      ......................................................................
PtOEP80b                      ......................................................................
MtOEP80b                      ......................................................................
psOEP80tr                     ......................................................................
SlOEP80b                      ......................................................................
BdOEP80b                      ......................................................................
SbOEP80b                      ......................................................................
ZmOEP80b                      ......................................................................
OsOEP80b                      ......................................................................
HvOEP80b                      ......................................................................
AcOEP80b                      ......................................................................
NmiOEP80                      .......GA.......................MGRFKA......................VAWKLGRGA.
CsOEP80                       ...............................MEGQAGS................................
MpuOEP80                      ....PSRPC.....................NTASKSSVGV..................EPARGGLGEGR.
OlOEP80                       ......................................................................
AtrOEP80a                     FKHLMRPPC...............................FSPQNPIEILHSLINSFTNPLKFYL.....
AtrOEP80b                     ......................................................................
Gloeobacter                   ......PPAA...............................APEAPPAG..........PAQ........
Synechocystis6803             .VEAVEMEA..............TEETTGVTEETPEETPSFTPDAPPTNTE......GTPG.........
Cyanothece8802                AAESTPLPT.........TPDSDLPPQTQA...........QETPPPTPTE.......TPT.........
Cyanothece51142               AAEAT...................PQQETA...........QETAPPNNNR...................
Oscillatoria                  .TSSKRNPALDG......TPVGYVPHSPRDIAQ....APLPPDNSAPESP.......TP...........
Microcoleus                   .SEAAVTPAWIA......AELG.KPEESGALAQNAQVTPRPAPTRPPANT.......VP...........
Microcystis                   ............................................SPPATGE......TTP..........
Nostoc73102C                  .S.....PT......PETTPV...PENVNPPTTEPLLPTTPEPSTAPDVP........FPDGQ.......
Anabaena_variabilis           .S.....PQKKAN.LPSPVPN...PLDTGATTAKQLVQAPEQPAPQPEVA........PPSTE.......
Trichodesmium101              STETKSEPLVNKQ.FIANIPQGKIVEKESLDSQNMVLSKSTETKSEPLVNK..QFIANIPQGKIVEKESL
Thermosynechococcus           ......QPA.............................ATPAPPTPPS......................
Synechococcus8102             ......................................................................
Synechococcus8109             ......................................................................
Nostoc7107                    .STI..EPA.....FPTTPGNTQPENTTPSTIEPAFPTTP.GTTQPENT.........TPSTI.......
Gloeocapsa                    .SQLQ.VPI.........TPE..APLDAPPGE.....ESVPAPATPPQ......................
Nostoc7120a                   ......................................................................
Nostoc7120b                   .........................................GNVTPPETEVTPQFSTKSRVIAQ......
Nostoc7120c                   .S.....PQKTAN.LPSPAPN...PLETRATTAKQLVQAPEQPAPQPEVA........PPTTE.......
Prochlorococcus_marinus_1375  ......................................................................
Prochlorococcus_marinus_1986  ......................................................................
Prochlorococcus_marinus_9313  ......................................................................
Nostoc73102d                  .S.....PS......PSTTPG...GENVKLPAATPV..................................
Nostoc73102b                  .........................................ETIASPETIVAQQFS.QNPVAAQTGA...
Nostoc73102a                  ......................................................................
Synechococcus_sp._JA-3-3Ab    .......AAW...............................AQSAPPPGTLDLRTNGDTPAQ........
Synechococcus_sp._JA-2-3B’a   .......PAW...............................AQSVPQPGTLDLRTGGDTPAQ........
alToc75c                      .......................................................RGG...GGGGKGGGR
alToc75a                      ...PSVLTG.GG.........G.................................GGG...GGDGNFGGF
alToc75b                      ......................................................................
atToc75a                      ...PSVLT.............G.................................GGG...GGDGNFGGF
psToc75                       ....SPFPNFSGLNAAAGGGAG.................................GGG...GGSSSSGG.
acToc75                       ...PNLFSSFVG.........G.................................GGG...GGGGWNGG.
atToc75b                      ......................................................................
atToc75c                      ......................................................................
vcToc75                       ..AFALVPNFGG........FG...............................GSGSG.RGGGGGGGGG.
smToc75                       ......................................................................
ppToc75a                      ..STATILALSG.........G................................DGGG..FGGFISGGG.
ppToc75b                      ...GTALLALGG..........................................DGGG..FGGFGSGGG.
ppToc75c                      ..STGTIPALSA.........G................................DGGG..FGGFISGGG.
zmToc75                       .FLLGSFGGFGG.........G.................................AGG.LFGGGGGGGGW
bdToc75a                      .......SGFGG.........G.................................SGGPL.GGGGGWGG.
osToc75a                      .......GGFGG.........G.................................AGGPLGGGGGGWGA.
osToc75b                      ......................................................................
crToc75                       ..AFVLVPNFGG........FG...............................GNGGN.RGGGGGGGGG.
bdToc75b                      ......................................................................
GsToc75                       ......................................................................
CmToc75                       .....................G...............................SIGDRTLRGGGGRGGN.
mpToc75                       ....SAISILGG.........G.................................NGG...GWGGEGGGF
pgToc75                       ......................................................................
ptaToc75                      ......ASPPGG.........G.................................NGG...GWMGGWGG.
pmToc75                       ......ASPPGG.........G.................................NAG...GWMGGWGG.
atrToc75                      .....RLSSYGG.........N.................................GGG...GGGGNGGG.
MtToc75                       .....PDFNFSG.........G.................................GGG...GGGSSSGG.
GmToc75                       .FLPNPLAKLGG.........G.................................GGN...TGGGGGGGD
ptToc75                       ......IDSGGG.........D...............................WFGGGSWLGGGGGGGG.
slToc75                       ...SDVLLSGNG.........G.................................GGS...GRGGSGGG.
hvToc75                       .......GGFGG.........G.................................PGGPL.GGGGGWGG.
sbToc75                       .FLLGSFGGFGG.........G.................................AGG.LFGGGGGGGGW
NmiToc75                      .....................G.................................GGG...GGDGDGGSF
mpuToc75                      ...IQPMHALQL.......AFG.................................GGG...GGDANGGG.
csToc75                       ......................................................................
olToc75                       ......................................................................
EcBamA                        ......................................................................
EcTamA                        ......................................................................
NmOMP85                       ......................................................................
BpFhaC                        ......................................................................
ScSam50                       ......................................................................
NcTob55                       ......................................................................
```

```
                                     110            120                                    130
AtOEP80a                      ........ENVE......TIRGLSSP.........................LLCCASLS.LTRPN.....
AlOEP80a                      ........ENVE......IIRGLNSP.........................LLCCASLS.LTRPN.....
GmOEP80a                      ...................................................LLCSATLS.LTG.......
MtOEP80a                      ...................................................SLCSTSLS.LNA.......
PsOEP80                       ......................................................................
SlOEP80a                      ......................SNTP.........................LLCCASIA.LAQSNL....
HvOEP80a                      .........................P.......................THALLSAALPPFAH.......
SbOEP80a                      .........................P.......................APALLSAALP.FAH.......
PtOEP80a                      ........PK.............SLP.........................ILCSASLS.LSQSQLR...
AcOEP80a                      ...................................................LLCSSSLA.LARQE.....
BdOEP80a                      .........................P.......................AHALLSAALP.FAH.......
OsOEP80a                      .........................P.......................APTLLSAALP.FAR.......
ZmOEP80a                      .........................P.......................APALLSAALP.FAH.......
PpOEP80                       ..........SL......IKHANFKP..........FMYMNA........APIRLASLS.MS..Q.....
SmOEP80a                      ..........IL......GSLRQR................HA........ALFCSASLG.AGD.D.....
SmOEP80b                      ..........IL......GSLRQR................HA........ALFCSASLG.AGD.D.....
CrOEP80                       .....................GGAGP.........................GALSGALA.TAGGGS....
VcOEP80                       .........................P.........................RAVSSLLS.C.........
MpOEP80                       ........NKFL......LGHRERWE..........NRFLSG........AAICSTSLA.LADNR.....
PgOEP80                       ......................RRRP.........................LLCSTSLA.IANRENKPPS
PtaOEP80                      ......................RRMP.........................LLCSTSIA.IANRENKPPS
PmOEP80                       ......................RRMP.........................LLCSTSIT.IANRENKVPS
AtOEP80b                      ......................................................................
AlOEP80b                      ......................................................................
AtOEP80c                      ......................................................................
GmOEP80b                      ......................................................................
PtOEP80b                      ......................................................................
MtOEP80b                      ......................................................................
psOEP80tr                     ......................................................................
SlOEP80b                      ......................................................................
BdOEP80b                      ......................................................................
SbOEP80b                      ......................................................................
ZmOEP80b                      ......................................................................
OsOEP80b                      ......................................................................
HvOEP80b                      ......................................................................
AcOEP80b                      ......................................................................
NmiOEP80                      ........ARC..........LERIPRGGHVASLDDVGYMTAGKD.....MAFCSTSLS.MSSSSWNEKR
CsOEP80                       .....................DTAGP.........................GT.................
MpuOEP80                      ........GGV..........LLRAPPGGA.....................PVTTHHLA.DVADAQP...
OlOEP80                       ......................................................................
AtrOEP80a                     .....................LRPSP.........................LLCSATLS...........
AtrOEP80b                     ......................................................................
Gloeobacter                   .........................PAET........................PPAA.............
Synechocystis6803             .........................PTQT.......................LPSFT.............
Cyanothece8802                .........................PVES........................PSDQ.............
Cyanothece51142               ...........................ED........................PDEA.............
Oscillatoria                  .........................PVDI........................PAPS.............
Microcoleus                   .........................PL...........................................
Microcystis                   .........................PVAA........................PTET.............
Nostoc73102C                  ......................QRTPAPR........................PGATP............
Anabaena_variabilis           ......................EPAPAPE..........................TT.............
Trichodesmium101              ......DSQNVVLS...KSTETKSEPLVNKQFIA.................NIPQGKIV.E.........
Thermosynechococcus           .........................PEPV........................PESP.............
Synechococcus8102             ......................................................................
Synechococcus8109             ......................................................................
Nostoc7107                    ......................E..PA.F........................PGNT.............
Gloeocapsa                    .........................PAPL........................PEGV.............
Nostoc7120a                   ......................................................................
Nostoc7120b                   .......................NSPV........................VLPSTPR............
Nostoc7120c                   ......................QPAPAPA........................PGTT.............
Prochlorococcus_marinus_1375  .........................KNDN.........................................
Prochlorococcus_marinus_1986  .........................KKNN.VLIV.................D..................
Prochlorococcus_marinus_9313  .........................PAEQ.QEL.....................................
Nostoc73102d                  ......................................................................
Nostoc73102b                  .....................SKNSPV........................VLKSAQEL...........
Nostoc73102a                  ......................................................................
Synechococcus_sp._JA-3-3Ab    .........................PSPT........................PT...............
Synechococcus_sp._JA-2-3B'a   .........................PSPT........................PP...............
alToc75c                      ........GG..................GG..........NFGGDGGFWRKLFSLAVPV.AVA.......
alToc75a                      ........GG..................GD............GNDGGFWGKLFAPAPAV.A.........
alToc75b                      ......................................................................
atToc75a                      GGG....GGG..................GD............GNDGGFWGKLFSPSPAV.A.........
psToc75                       ........GG..................GG.......WFN..GDEGSFWSRILSPARAI.A.........
acToc75                       ........GG..................GG............GGNDGFWSRLFSPAAIA.KD........
atToc75b                      ......................................................................
atToc75c                      ......................................................................
vcToc75                       ........GG...............PDSG...........NGQPDGGWAIPLFDLAASA...........
smToc75                       ......................................................................
ppToc75a                      ........GG...............................GGGGWRFWLENIAWADAR.K.........
ppToc75b                      ........GG..................GG............GGDGWRFW...LDSGIAW.ADAR.....
ppToc75c                      ........GG..................GG...........GGGGWRFWLENIAWADAR.K.........
zmToc75                       GA......GG..................GGA.........GGGGGGDFWSWLFSAGAAN.A.........
bdToc75a                      ........GG..................GGAGGGA.....GGGGGGSFWSRIFSAGAAN.A.........
osToc75a                      ........GG..................GGGGG.......GGGGGGGFWSRIFSGGAAH.A.........
osToc75b                      ......................................................................
crToc75                       ........GG.................SGG..........QGQPDGGLPLPLYELAEES.S.........
bdToc75b                      ........GG..................GG.................FLSWLFAAGPAH.AAA.......
GsToc75                       .AYSHGSGSGK..............P.........................PFSSSFHS.E.........
CmToc75                       .SYVDGVAGGF..............P............................................
mpToc75                       ........GG..................GG...........DGSGGNGWRSVFDNGMAF.AD........
pgToc75                       ......................................................................
ptaToc75                      ........GG..................DG............GG..NFWSRIGAALADA.K.........
pmToc75                       ........GG..................DG............GG..NFWSRIGAALADA.K.........
atrToc75                      ........GG..................DS............GGFGRFWSELLSPVAVA...........
MtToc75                       ........GG..................GG.......WFNNGGDGGDFWSRILSPSTAF.A.........
GmToc75                       GW.....FGG..................GG............GGNGGFWSRMFAAVA.............
ptToc75                       ........GG..................DG............GSGGEFWKNLFSVASAN.A.........
slToc75                       ........GG..................GG............GD..SFWSKIFSPAAIA...........
hvToc75                       ........GG..................GGAGAG......GGGGGGGFWSRIFSAGAAN.A.........
sbToc75                       ........GA..................GGA.........GGGGGGDFLSRLFSVGAAH.A.........
NmiToc75                      LQY....DGGMALAEAEEGAQSDSDDEEGG..........DEGKGGGDEEREDGGGAAG.S.........
mpuToc75                      ........GG..................GG............GGENNRFLGSVLAT..............
csToc75                       ........GG...............EVAAG..........EEGDD.........................
olToc75                       ......................................................................
EcBamA                        ...................................................LFSSATVY.G.........
EcTamA                        ...................................................CLSGSAVA.ANV.......
NmOMP85                       ......................................................................
BpFhaC                        ........AGL........................................LFAVAACA.Q.........
ScSam50                       ......................................................................
NcTob55                       ......................................................................
```

```
                              140                                                 150
AtOEP80a                      ..........ESTQSV...............EGK...........................DTVQQQKG.
AlOEP80a                      ..........ESTQSV...............EGK...........................DIVQQQKG.
GmOEP80a                      ...............................DRKRKC..........PIRR........LASLSLAEEA.
MtOEP80a                      ................................ANRSP..........PL............SLSSAEET.
PsOEP80                       ......................................................................
SlOEP80a                      ...............................DGT.............PLSG...................
HvOEP80a                      ..........................IGRAIDAAARRVAAS..FPRVPAARAET.........PAAP...
SbOEP80a                      ..........................IGRAIDAAARRLGSC..LPRVPVARADP.........AAPP...
PtOEP80a                      ..........DSTQSD.............................................SVVAQQKSG
AcOEP80a                      ..........DLNQNV.............LVDGKEPP..........PLK...............AQLV.
BdOEP80a                      ..........................IGRAIDAAARRVAAS..FPRVPAARAET.........AAAP...
OsOEP80a                      ..........................IGRAIDGVVRHVARS..LPRLPVARAET.....GAGAAAAPIA.
ZmOEP80a                      ..........................IGRAIDAAARRLGAC..LPRVPVARADT.........APPP...
PpOEP80                       ...............................DQSGDP..........KVESVR..........PSRRKV.
SmOEP80a                      ...............................QRKENS..........MAVAVR.........KGSRGK..
SmOEP80b                      ...............................QRKENS..........MVVAVR.........KGSRGK..
CrOEP80                       .........AEAKAKA...............GGAADG..........AVGAGG.......GGGGGKGKS.
VcOEP80                       ......................................................................
MpOEP80                       ...............................DYKRPE..........KIAADRL.P...GGGQGSSRKG.
PgOEP80                       QE.......LESSQNASKDHASTTESLNDLSNGDSQR..........YTVRDT.......EDTAGQQPK.
PtaOEP80                      LE.......LESSQNANKDYASTTDGLNDLSNGDSHR..........HTVQDT.......EDTAGQQQK.
PmOEP80                       LE.......LESSQNAGKDYASTSETLTDLSNGDSHG..........PTVQGT.......ADTAGQQQK.
AtOEP80b                      ......................................................................
AlOEP80b                      ......................................................................
AtOEP80c                      ......................................................................
GmOEP80b                      ......................................................................
PtOEP80b                      ......................................................................
MtOEP80b                      ......................................................................
psOEP80tr                     ......................................................................
SlOEP80b                      ......................................................................
BdOEP80b                      ......................................................................
SbOEP80b                      ......................................................................
ZmOEP80b                      ......................................................................
OsOEP80b                      ......................................................................
HvOEP80b                      ......................................................................
AcOEP80b                      ......................................................................
NmiOEP80                      GDGDGDKVWAEEQQSA...............DVEESE..........KLLSAK........GGGARRRSG
CsOEP80                       ......................................................................
MpuOEP80                      .........QQQQQQQ...............QAAETP..........PPSSSS........SSVSHDDAN
OlOEP80                       ......................................................................
AtrOEP80a                     .............SSF...............EGQSP.........................GVLVHQKG.
AtrOEP80b                     ......................................................................
Gloeobacter                   ...................................TP........AVPDT....PP...AEPQQAPTE..
Synechocystis6803             ....................................P..........PAS.....PST.TTPA.......
Cyanothece8802                ....................................P..........PSV.....PEI.PTPTQPPASQT
Cyanothece51142               ....................................D..........P.......PVV.PTPEVIPAPE.
Oscillatoria                  ....................................P........EAPPA.....P...PVPRTTPTP..
Microcoleus                   ...............................................PPG.....PA..PQPRQTPA...
Microcystis                   ...............................................PTET........PSPAETPTTAP
Nostoc73102C                  .............NPQN.................VNP..........PTT.....PES.TQPNTAPEA..
Anabaena_variabilis           ...............................................PGT.....ENF.NTPNATPET..
Trichodesmium101              .........KESLDSQN.......VVLSKSTETKSEPLVNKQFTANIPTTEIVTESEKYISPQSQQAQT.
Thermosynechococcus           ...............................................PAA.....PST.PAPVGQPPA..
Synechococcus8102             ......................................................................
Synechococcus8109             ......................................................................
Nostoc7107                    .............QPEN.................TNPPT..I.EPAFPTT.....PGN.TQPNGATET..
Gloeocapsa                    .............QPE..................VAP..........PLT.....P....QPEAQPEAT.
Nostoc7120a                   ...............................................P......................
Nostoc7120b                   ...............................................PVV.....P...PAP........
Nostoc7120c                   ...............................................PGT.....ENF.NTPNATPET..
Prochlorococcus_marinus_1375  .......................................................PVE.ITESNTKKDSI
Prochlorococcus_marinus_1986  ...............................................................NGKRD..
Prochlorococcus_marinus_9313  .......................................................PLN.ASEQLS.....
Nostoc73102d                  ....................................S..........PTT.....PRN.IKPNTAPEA..
Nostoc73102b                  ...............................NSQAIP..........PAS.....PW..PSP........
Nostoc73102a                  ......................................................................
Synechococcus_sp._JA-3-3Ab    ......................................................................
Synechococcus_sp._JA-2-3B’a   ......................................................................
alToc75c                      ..........DEEQSPD.............WDSHGLPA.....NIVVQLNKLSG................
alToc75a                      ..........DEEQSPD.............WDSHGLPA.....NIVVQLNKLSG................
alToc75b                      ......................................................................
atToc75a                      ..........DEEQSPD.............WDSHGLPA.....NIVVQLNKLSG................
psToc75                       ..........DEPKSED.............WDSHELPA.....DITVLLGRLSG................
acToc75                       ..........EESESQD.............WDPHGLPA.....NIIVPLSKLSG................
atToc75b                      ......................................................................
atToc75c                      ......................................................................
vcToc75                       .............EGKE.............TD.................SKKGR................
smToc75                       ......................................................................
ppToc75a                      ..........DEEGADG.............FDPHGLQV.....GRTVPVSKLNP................
ppToc75b                      .........NDEEGGDA.............FDPHGLQV.....GRNVPVSRLNP................
ppToc75c                      ..........DEEGADA.............FDSHGLQV.....GRTVPVSKLNP................
zmToc75                       ..........DDKSSGD.............WDAHGLPV.....NMTVPLTKLSG................
bdToc75a                      ..........DEKSSGD.............WDPHGLPA.....NITVPLSKLSG................
osToc75a                      ..........DEKSSGD.............WDPHGLPA.....NINVPMTKLSG................
osToc75b                      ......................................................................
crToc75                       ..........DEKEKQQ.............KD.................DKR..................
bdToc75b                      ..........EPSSKQG.............WDAHGLGA......RAVPLARLDG................
GsToc75                       .............................................ESPLD.....PED............
CmToc75                       .............................................DLP......................
mpToc75                       .........GRNDESEQ.............FDSHGLRV.....DMRVHITKLNP................
pgToc75                       ......................................................................
ptaToc75                      ..........KEDESEA.............FDSHGLPA.....GFAVQPNRLSA................
pmToc75                       ..........KEDESEA.............FDSHGLPA.....GLAVQLNRLSA................
atrToc75                      ..........KEEENQE.............WDPHGLPA.....NILVQINKLSG................
MtToc75                       ..........DEPKSQD.............WDSHELPA.....NITVLPNKLSG................
GmToc75                       ..........DESQSQE.............WDSHGLPA.....NIVVQLNKMSG................
ptToc75                       ..........DESQSQD.............WDSHGLPA.....NIVVQLNKLSG................
slToc75                       ..........GEEESQE.............WDSHGLPA.....NIVVQLNKLSG................
hvToc75                       ..........DDKSSAD.............WDPHGLPA.....NITVPLSKLSG................
sbToc75                       ..........DDKSSGD.............WDAHGLPV.....NMTVPLTKLSG................
NmiToc75                      ..........QKTASDQ.............FQ....RI.....QKTIPWDKLQE................
mpuToc75                      ..................................NLPT................................
csToc75                       ..........DNEDYTD...LSKGTFFCADVKANGLPV................................
olToc75                       ......................................................................
EcBamA                        ......................................................................
EcTamA                        ......................................................................
NmOMP85                       ......................................................SPLA............
BpFhaC                        ................................AQLLPGARD.............................
ScSam50                       ......................................................................
NcTob55                       ......................................................................
```

i.

```
                                     160       170                 180
AtOEP80a                        .....H..SVSRNA......EERVLISEVLVRTKDGEE...................LERKDLEMEALAA
AlOEP80a                        .....H..SVSRNA......EERVLISEVLVRTKDGEE...................LERKDLEMEALAA
GmOEP80a                        .....Q..QKARQN......EERVLISEVLVRNKDGEE...................LERKDLEAEAAQA
MtOEP80a                        .....Q..LKTRQN......EERVLISEVLVRNKDGEE...................LERKDLEAEAAQA
PsOEP80                         ......................................................................
SlOEP80a                        .....P..KTGSGN......EERVLISEVLVRNKDGEE...................LERKDLESEALNA
HvOEP80a                        ....LP..RRHGKDGGGGGGEERVLISEVAVRGKDGEP...................LERPELEEAAAAA
SbOEP80a                        .....PLARRHGKD..GGGPEERVLISEVAVRGKDGEP...................LERAELEAAAAAA
PtOEP80a                        GASGVH..GPSRYD......EERVLISEVLVRNKDGEE...................LERKDLEAEALAA
AcOEP80a                        .....S..RVGRED......EERVLISEVLIRNKDGDE...................LERKDLEEEAAAA
BdOEP80a                        ....LP..RRHGKD..GGGGEERVLISEVAVRGKDGEP...................LERPELEEAAAAA
OsOEP80a                        ....LP..RRQKDGGGGGGGEERVLISEVAVRGKDGEP...................LERPELEAAAAAA
ZmOEP80a                        .....P..RRHGKD..GGGAEERVLISEVAVRGKDGEP...................LERPELEAEAAAA
PpOEP80                         PGLQIG..QEAPQE......EERVLISEILIQDKDGQE...................LTDPELNSTAWQA
SmOEP80a                        ............EE......EERVLISEVLVQDKDGQE...................LENSEILATARQA
SmOEP80b                        ............EE......EERVLISEVLVQDKDGQE...................LENSEILATARQA
CrOEP80                         ............EP......EDRILISEVEVVGCDG.........................ELKSIAKQA
VcOEP80                         ....................WLWLLRCEVEVVGCDG.........................ELKQIAREA
MpOEP80                         ....LSV.PAGAED......EERVLISEILIQDKDGQD...................LEDAELLATVRCA
PgOEP80                         .QACVC..RPANED......QERVLISEVLFQNKDGDE...................LENPELLSSAMGA
PtaOEP80                        .QACVC..RHAKED......QERVLISEVLFQNKDGDE...................LENPELLSSAMGA
PmOEP80                         .QACLC..RPAKED......QERVLISEVLFQNKDGDD...................LDNPELLSSAMGA
AtOEP80b                        ......................................................................
AlOEP80b                        ......................................................................
AtOEP80c                        ......................................................................
GmOEP80b                        ......................................................................
PtOEP80b                        ......................................................................
MtOEP80b                        ......................................................................
psOEP80tr                       ......................................................................
SlOEP80b                        ......................................................................
BdOEP80b                        ......................................................................
SbOEP80b                        ......................................................................
ZmOEP80b                        ......................................................................
OsOEP80b                        ......................................................................
HvOEP80b                        ......................................................................
AcOEP80b                        ......................................................................
NmiOEP80                        QGARMV..EDATEE......EERVLISEILVKDMDGQD...................LEDQELADIARKS
CsOEP80                         ....................EEPILISEVEVRGVDG.........................ELKRIAESK
MpuOEP80                        .....G..GDSEED......ERPILISSV...DFDG......................ETPELEQLARSV
OlOEP80                         ......................................................................
AtrOEP80a                       .....S..RHGRED......QERVLISEVLVQNKDGEE...................LENKELAGVAVSA
AtrOEP80b                       ......................................................................
Gloeobacter                     .........PAAPA......EPQVLVAEVRVVASKGK.....................LGDTLEGRVYNA
Synechocystis6803               .........PAEEE.......PRVLVSEVLVTGTT.........................PELELLVYNA
Cyanothece8802                  .........PLTEE..SPGPEPQVLVVEVVVSGAE.........................TELENLVYNT
Cyanothece51142                 .........PETEETDTTGEEPRVLVVEVLVEGAD.........................PELQDLVYNT
Oscillatoria                    .........PAGQS......DSQVLVAEVVVSGVEG.........................QLEDEVYRV
Microcoleus                     .........PAGAA......EPQVLVAEVTVTGATD.........................DLESEIYRV
Microcystis                     .........PVPAE......ESRVLVAEVVVQGAD.........................RELENLVYNT
Nostoc73102C                    .............N......DPRVLVSEVLIRSQSGQ.....................LSPELEEQVYRV
Anabaena_variabilis             .............T......EPRVLVSEVLVRPQSGQ.....................LTPELEAQVYNV
Trichodesmium101                .........STEEE......EPLVLVAEVVVTGVDG.........................ELQDEVYRV
Thermosynechococcus             .............D......EPKVLIAEVVVEGAT.........................PELEQLVYQV
Synechococcus8102               .....................PRVLISEVLIEGIGGHP....................EEERLQVAAYGA
Synechococcus8109               .....................PRVLISEVLIEGIEGHP....................EEERLQISTYDA
Nostoc7107                      .............T......EPRVLVSEVLVRPQTGQ.....................LTPELEEKVYQV
Gloeocapsa                      .........P..DT......APQVLVSEVVVSAETGV.....................LDPELENQVYQA
Nostoc7120a                     ....................IVVILVVGLLVNLEHQQV................................
Nostoc7120b                     ....................KPATTVNELVVTATDVQIV..................GATPELQEIIRQV
Nostoc7120c                     .............T......EPRVLVSEVLVRPQSGQ.....................LTPELETQVYNV
Prochlorococcus_marinus_1375    .........ENDNE......ATLVLISEVVIKGLENHP....................DEDRLRYAAYDA
Prochlorococcus_marinus_1986    .............S......EENVLISEIIIEGWENHP....................EGRKLELAAYDS
Prochlorococcus_marinus_9313    .............E......EPRVLITEVIIEGIAGHP....................EQERVELAAYDA
Nostoc73102d                    .............N......DPRVLVSEVVVKSQTGQ.....................LSPELENQVYRV
Nostoc73102b                    ....................KTPATESTLVVTATDVQVV..................GANQELQEIIRKV
Nostoc73102a                    ....................ITVVAIAVMLVGGKNPEA................................
Synechococcus_sp._JA-3-3Ab      .........PAA.G......EPEVLVAEVVV...EGT.....................DDPELIEQVYKA
Synechococcus_sp._JA-2-3B’a     .........PTA.G......EPEVLVAEVVV...EGT.....................EDPELINRVYSV
alToc75c                        ....................LKKYKISDIMFFDRRR.....................KATVETDDSFFEM
alToc75a                        ....................FKKYKVSDIMFFDRRR.....................QTTIGTEDSFFEM
alToc75b                        ......................................................................
atToc75a                        ....................FKKYKVSDIMFFDRRR.....................QTTIGTEDSFFEM
psToc75                         ....................FKKYKISDILFFDRNK.....................KSKVETQDSFLDM
acToc75                         ....................FKKYKISEVLFLDLWN.....................RIIGGPEDSFFEM
atToc75b                        ......................................................................
atToc75c                        ......................................................................
vcToc75                         ....................NWKDLITDTEDLEHKPGERTGTNRCVEIVIEGW..PEVGNLPTESELKDL
smToc75                         ......................................................................
ppToc75a                        ....................AKRYKVSEVELIDKRT.....................NQPVNSKDAFYEL
ppToc75b                        ....................AKRYRISEVELVDKRT.....................KQPIDAKDAFYEL
ppToc75c                        ....................SKRYKVAEVVLIDKRT.....................NQPVNVKDAFYEL
zmToc75                         ....................LKRYKLSELKFFDRAA..................AGGGAYTGPEDSFFEM
bdToc75a                        ....................LKRYKLSELKFFDRAAPA................GSGGTEAGPEDSFFEM
osToc75a                        ....................LKRYKISELKFFDRAA..................GGGGAFTGPEDSFFEM
osToc75b                        ......................................................................
crToc75                         ....................NWKNLVTDSEDLEEKPGERSGTNRCVEIVIEGW..PDVGNLPTADELKDL
bdToc75b                        ....................RKRYKVSDVSFLDATT..................PRCGGRTGPEDPLFES
GsToc75                         ....................HSLLLVGGLSLND........................WLNKLVKRESVNS
CmToc75                         ...................NRGSFLL..........................WLTLCWHTVQVWLRAPTA
mpToc75                         ....................SKKYKVSGVELVDKRT.....................KQAVSSKDPFYEL
pgToc75                         ......................................................................
ptaToc75                        ....................SKRYKVSGVDLYDIRN.....................SKTILPSDPFYEL
pmToc75                         ....................YKKYKLSGVELFDGRT.....................QKSVGSNDPFYEL
atrToc75                        ....................FKKYKTSEILFFDKKR.....................GSVVGTEDSFFEM
MtToc75                         ....................FKKYKISDIIFLDRNT.....................KTKIGTEDSFLDM
GmToc75                         ....................FKKYKVSDISFFDRNR.....................KMKVGTEDSFFEM
ptToc75                         ....................FKKYKLSEILFFDRRR.....................WTTVGTEDSFFEM
slToc75                         ....................FKKYKVSDILFFDRRR.....................GSTVGTEDSFFEM
hvToc75                         ....................LKRYKLSELKFFDRAAPG................GGGATDAGPEDSFFEM
sbToc75                         ....................LKRYKLSELKFFDRAA..................AGGGAYTGPEDSFFEM
NmiToc75                        ....................NKRYSAEEVVLVDLIT.....................KQVIQKKDELYNH
mpuToc75                        .........................................................GPGIPTKAELFGG
csToc75                         .........................................................GPGVPTEEELFAG
olToc75                         ......................................................................
EcBamA                          ....................AEGFVVKDIHFEGLQRVAVG.........................AALLS
EcTamA                          ...........................RLQVEGLSG.........................QLEKNVRAQ
NmOMP85                         ....................FADFTIQDIRVEGLQRTEPS.........................TVFNY
BpFhaC                          ......................................................................
ScSam50                         ......................................................................
NcTob55                         ......................................................................
```

```
                                     190       200       210       220       230       240
AtOEP80a                      LKACRANSALTIREVQEDVHRI.....IESGYFCSCTPVA.....VDTRDGIRLMFQVEPNQEFRGLVCE
AlOEP80a                      LKACRANSALTIREVQEDVHRI.....IESGYFCSCTPVA.....VDTRDGIRLMFQVEPNQEFRGLVCE
GmOEP80a                      LKACRPNSALTVREVQEDVHRI.....INSGYFSSCMPVA.....VDTRDGIRLVFQVEPNQEFQGLVCE
MtOEP80a                      LKACRPNSALTVREVQDDVHRI.....INSGYFCSCVPVA.....VDTRDGIRLVFQVEPNQEFQGLVCE
PsOEP80                       ..................................SCVPVA.....VDTRDGIRLVFQVEPNQEFQGLVCE
SlOEP80a                      LKACRPNSALTVREVQEDVHRI.....VASGYFCSCMPVA.....VDTRDGIRLVFQVEPNQEFHGLVCE
HvOEP80a                      LRACRPNAALTVREVQEDVHRV.....VESGLFRSCMPVA.....VDTRDGIRLVFEVEPNQDFHGLVCE
SbOEP80a                      LRACRPNAALTVREVQEDVHRI.....VESGLFRSCMPVA.....VDTRDGIRLVFEVEPNQDFHGLVCE
PtOEP80a                      LKACRANSALTVREVQEDVHRV.....ISSGYFCSCMPVA.....VDTRDGIRLVFQVEPNQEFHGLVCE
AcOEP80a                      LKACRPNSALTVREVQEDVHRI.....MERGYFCSCMPVA.....VDTRDGIRLVFQVEPNQEFQGLVCE
BdOEP80a                      LRACRPNAALTVREVQEDVHRV.....VESGLFRSCMPVA.....VDTRDGIRLVFEVEPNQDFHGLVCE
OsOEP80a                      LRACRPNAALTVREVQEDVHRV.....VESGLFRSCMPVA.....VDTRDGIRLVFEVEPNQDFHGLVCE
ZmOEP80a                      LRACRPNAALTVREVQEDVHRI.....VESGLFRSCMPVA.....VDTRDGIRLVFEVEPNQDFHGLVCE
PpOEP80                       LKACKPNFALTTSEVQADVHRI.....IDTGFFASCMPSA.....EDTRDGVRLIFKVEPNQELRGLVCN
SmOEP80a                      LKSCKSNYALTTKEVQEDVHRI.....IETGFFASCMPVA.....EDTRDGVRLIFKVEANQELRGLVCN
SmOEP80b                      LKSCKSNYALTTKEVQEDVHRI.....IETGFFASCMPVA.....EDTRDGVRLIFKVEANQELRGLVCN
CrOEP80                       L.TTRPNFAYTLEEVKADVRRV.....FATGWFSEVSPDA.....EDTRDGVKLRIRVKPNEEVRSLTAA
VcOEP80                       L.TTRPNFAYTLEEVKADVRRV.....FATGWFAEVSPDA.....EDTRDGVKLRIRVRPNEEVRSLSAV
MpOEP80                       LKACKPNFALTTSEVQEDVHRI.....IDTGFFASCMPTA.....EDTRDGVKLIFKVESNQELKGLVCN
PgOEP80                       LKACKPNSALTIQEVQEDVHRV.....IDCGYFYSCMPVA.....VDTRDGIRLIFKVEPYQEFKGLICE
PtaOEP80                      LKACKPNSALTIQEVQEDVHRV.....IDCGYFYSCMPVA.....VDTRDGIRLIFKVEPYQEFKGLICE
PmOEP80                       LKACKPNSALTIQEVQEDVHRV.....IDCGYFYSCMPVA.....IDTRDGIRLIFKVEPYQEFKGLICE
AtOEP80b                      ............MGAQKSIHAG................................................
AlOEP80b                      ............MGAQKSIHAG................................................
AtOEP80c                      ......................................................................
GmOEP80b                      ............MGAQKSIHAG................................................
PtOEP80b                      ............MGAQKSIHAG................................................
MtOEP80b                      ............MGAQKSIHAG................................................
psOEP80tr                     ............MGAQKSIHAG................................................
SlOEP80b                      ............MGAQKSIHAG................................................
BdOEP80b                      ............MGAQQSVNAG................................................
SbOEP80b                      ............MGAQQSVNAG................................................
ZmOEP80b                      ............MGAQQSVNAG................................................
OsOEP80b                      ............MGAQQSVNAG................................................
HvOEP80b                      ............MGAQQSVNAG................................................
AcOEP80b                      ............MGAQKSIHAG................................................
NmiOEP80                      LKTSKPNFAFTAREVQEDVHRI.....IDSGYFASCLPTA.....EDTRDGVRLVFKVEPNQEMKGLIMN
CsOEP80                       L.TIKPNFAYTLSEVRDDIQRV.....FDAGYFQQILPAA.....EDTRDGIKLTLQATANPVIKGVVVT
MpuOEP80                      V.FIKPNFSYTKREIQTDLQRI.....FDTGKFSTVAPMT.....VDTRDGVALTFKLVANPVVRGFIVK
OlOEP80                       .MTLKPNYSYRAREVAGESHVIK....SELGIFKSVRALA.....KDTRDGMRVTFEVVPFEILRGVELQ
AtrOEP80a                     LKSCRPNSALTVQEVQEDVHRI.....IDSGYFQSCMPVA.....VDTRDGIRLIFQVEPNQEFKGLICE
AtrOEP80b                     ......................................................................
Gloeobacter                   I.RTRPGQTTTRTQLQQDINAV.....FATGYFADVKATP.....EDTPLGVRVTFDVSPNPTLEAVSTE
Synechocystis6803             I.RTQPGRTTTRTQLQEDVNAI.....YATGYFSNVRVAP.....SDTPLGVRVTFEVQANPVFTGLNIR
Cyanothece8802                I.QTRPGRTATRSQLQEDVNAI.....YATGFFANVKVTP.....ADTPLGVRITFEVDPNPTLAKVVIE
Cyanothece51142               I.QTRPGRTATRSQLQEDVNAI.....YATGYFANVEVTP.....ADTPLGVRITYNVEVNPVLEQVVVN
Oscillatoria                  I.STQPGRTTTRTQLQQDINAI.....FATGFFRNVRAVP.....EDTPLGVRVTFVVQANPILRGVQIT
Microcoleus                   I.STQPGRTTNRSQLQQDINAI.....FATGFFRNVRAVP.....EDTPLGVRVTFIVQPNPVLRGVTIT
Microcystis                   I.RTRPGRSTTRSQLQEDINAV.....FATGFFADVRAVP.....QDTPLGVRVTFEVQPNPVFQGVQIQ
Nostoc73102C                  I.RTQPGRTTTRSQLQEDINAI.....FGTGFFSNVQASP.....EDTPLGVRVSFVVQPNPVLSKVEVQ
Anabaena_variabilis           I.RTQPGRTTTRSQLQEDINAI.....FGTGLFSNVQASP.....EDTPLGVRVSFIVQPNPVLSKVEIQ
Trichodesmium101              I.NTQPGETTTRSQLQKDINAI.....FATGFFQNVKAIP.....EDTPLGVRIIFKVEPNPILTSVIIE
Thermosynechococcus           I.STRPGSTTTRTQLQQDTNAI.....FATGFFADVNAVP.....RDTPLGVRITFVVRPYPVLRAVQVA
Synechococcus8102             M.QVRPGSRVTREELQRDLNAI.....QATGWFSDVRINP.....VNGPLGVQVVVQVEPFPTLSRVELD
Synechococcus8109             M.RVRPGMRVNREELQNDLNGI.....QATGWFSDVRIVP.....QNGPLGVQVVVQVQPFPPLSAVEIN
Nostoc7107                    I.RTQPGRTTTRTQLQEDINSI.....FGTGFFSNVQALP.....EDTPLGVRVSFVVQPNPVLTKVQVE
Gloeocapsa                    V.RTVPGRTTTRTQLQEDINAI.....FATGFFSNVRAEP.....TDTPLGVRVTFVVQPNPVLRSVQVQ
Nostoc7120a                   ................................LADVPVGT.....KDLSDGKV.................
Nostoc7120b                   I.KTQTGGDTSQTQLQRDVAAI.....LETGLFASANVNS.....RTTPSGLNVVYQVQPV.IVRSLQLT
Nostoc7120c                   I.RTQPGRTTTRSQLQEDINAI.....FGTGFFSNVQASP.....EDTPLGVRVSFIVQPNPVLSKVEIQ
Prochlorococcus_marinus_1375  M.NIRPGSRVSRLQLKRDLNSI.....YATGWFSGLEIQP.....IDTPLGVQLSVNVQPNPIFTEVEII
Prochlorococcus_marinus_1986  M.TIKPGSVINNKKLKNDLNSI.....YASGWFSGVKIKS.....QDGPLGVRLIVSVVPNPILKKVKLN
Prochlorococcus_marinus_9313  M.VVRPGSRVTRDELKRDLDAI.....YATGWFSDVRIEP.....SDGPLGVQLVVQVQPNPLLTKVELD
Nostoc73102d                  I.RTQPGQTTTRSQLQEDINAI.....FGTGFFSNVQASP.....EDTPLGVRVSFVVQPNPVLSKVEVQ
Nostoc73102b                  I.KTQVGGETSQSQLQKDVATI.....LATGLFSNVSVNS.....FSTQAGLNVVYQVQPI.IVRSLQLS
Nostoc73102a                  .................................LADSPST.....QDGASGIV.................
Synechococcus_sp._JA-3-3Ab    I.RTRAGEVSTRSQLREDINAV.....FATGFFADVRAVP.....SDTPLGVRVTFEVKPNPILKAVRTE
Synechococcus_sp._JA-2-3B'a   I.RTRAGEVSTRSQLREDINAV.....FATGLFADVRAVP.....SDTPLGVRVTFEVKPNPILKAVRTE
alToc75c                      V.SIQEGGVYTKAQLQKELETL.....TTCGWFEKVDFEG..KTKPDTGLGVTISFAESMWQGAERFRCI
alToc75a                      V.SIRPGGVYTKAQLQKELETL.....ATCGMFEKVDLEG..KTKPDGTLGVTISFAESTWQSADRFRCI
alToc75b                      ......................................................................
atToc75a                      V.SIRPGGVYTKAQLQKELETL.....ATCGMFEKVDLEG..KTKPDGTLGVTISFAESTWQSADRFRCI
psToc75                       V.SLKPGGVYTKAQLQKELESL.....ATCGMFEKVDMEG..KTKPDGTLGLTISFAESMWERADRFRCI
acToc75                       V.SLRPGGIYTKATLQKELETL.....ATCGMFEKVELEG..KTNPDGTICINIAFTESTWQSADKFRCI
atToc75b                      ......................................................................
atToc75c                      ......................................................................
vcToc75                       L.TVQEGHIFEKQDLLDDRRKLE...IQYEDYIAEVEIRTEFVDDVSNHQRVVYRFT......PHKFRGI
smToc75                       .....................................MEG..VTQADGTIKININFVESQWQAAKSVRCI
ppToc75a                      V.TLRAGGVATKAQLEAEVENL.....MNCGMFQSVEMDG..VTNLDGSLKMQVSFIESQWQPADSFRCV
ppToc75b                      V.TLRRGGIATKAQLDAESENL.....MNCGMFQSVEMEG..VTKLDGSLKLQIMFTESQWQGADSFRCV
ppToc75c                      V.TLRAGGVATKAQLEAEVENL.....MNCGMFQSVEMDG..VTNLDGSLKMQISFTESQWQPADSFRCV
zmToc75                       V.TLQPGGVYTKSQLLKELETL.....VSCGMFERVDLEV..KPKPDNTIGLTVSFVESVWSAAKQFKCI
bdToc75a                      V.TLQPGGVYTKSQLLKELETL.....VSCGMFERVDLEG..KAKPDGTLGLTVSFVESVWSAAKQFKCI
osToc75a                      V.TLQPGGVYTKSQLLKELETL.....VSCGMFERVDLEG..KAKPDGTLGLTVSFVESVWSAAKQFKCI
osToc75b                      ......................................................................
crToc75                       L.TVQEGHIFEKQDLLDDRRKLE...IQYEDYIAEVEIRTEYVDGKSNHQRVVYKFT......PHQFRGI
bdToc75b                      S.SLRPGGVYTRAKILEELKAL.....SSSGLFERVRLNP.VRPNPDGTLALTVAYAESAWPAARRLRCA
GsToc75                       A.KPSEGLMNKDDEQNRKVRKIVIE.............................................
CmToc75                       L.ALSVPTAATEEEAAEIYAEHPND..QTKGPLRQVVLRG..............................
mpToc75                       V.TLRPGGIATKAQLESEVENL.....LNCGMFQTVEMEG..VTQADGSLKMQISFVESQWQPADSFRCV
pgToc75                       ......................................................................
ptaToc75                      L.SLKPGAIYTKAHLQAELDNL.....TSSGMFEKVDLEA..IAQPDGTIKLKFAFLESQWQSSESFRCI
pmToc75                       L.TLKPGAVYTKAHLQAEMDNL.....TSSGMFEKVDMEG..ITQPDGTIKLKFSFLESQWQSSDSFRCI
atrToc75                      V.SLKPGGIYTKAHLQKELETL.....ATCGMFEKVDLEA..KTKPDGTIAVNISFAESTWQSADAFKCI
MtToc75                       V.SLKPGGVYTRAQLQKEIESL.....ATCGMFEKVDMEG..KTNADGSLALTISFAESMWEKADRFRCI
GmToc75                       V.SLRPGGVYTKGQLQKELETL.....ATSGMFEKVDLEG..KTNPDGSIGVTISFSESTWQSADGFRCI
ptToc75                       V.SLRPGGVYTKAQLQKELESL.....ATCGMFEKVDMEG..KTNPDGTIGITISFTESTWQSADKFRCI
slToc75                       L.SLRPGGVYTKAQLQKELETL.....ATSGMFEKVDLDA..KTNPDGTVGVTISFLESTWQSADKFRCI
hvToc75                       V.TLQPGGVYTKSQLLKELETL.....VSCGMFERVDLEG..KAKPDGTLGLTVSFTESVWSAAKQFKCI
sbToc75                       V.TLQPGGVYTKSQLLKELETL.....VSCGMFERVDLEV..KPKPDNTIGLTVSFVESVWSAAKQFKCI
NmiToc75                      V.QIRPGGLFNKDEVNEDLQSL.....IETGLFVSVDAEV..QVGSQGGLRVQYGYREKRWPRPRSLKLM
mpuToc75                      L.NARKGNEVSKDDLSADLEAL.....LNTGLFANVDANV..TPKGKDGYAVEFVFREKIWPGVQTFKVK
csToc75                       L.NCQPGFVCSRSELSQDLRTL.....LQSGLFANVDARV..QHKGKGKYTLEFTFSENLWQPIKSFK..
olToc75                       ......................................................................
EcBamA                        M.PVRTGDTVNDEDISNTIRAL.....FATGNFEDVRVLR........DGDTLLVQVKERPTIASITFS
EcTamA                        LSTIESDEVTPDRRFRARVDDAIREGLKALGYYQP.............ADG....TIEFDLRPPPKKGRQVLI
NmOMP85                       L.PVKVGDTYNDTHGSAIIKSL.....YATGFFDDVRVET.....ADG....QLLLTVIERPTIGSLNIT
BpFhaC                        .LNRIDDRQRKEQLQRDIERALTRPPVELNPQSEAAAPA...RKPDATSGHTVTVH.............
ScSam50                       ........MTSSSGVDNEIS........................LDSPMPI.....FNESSTLKPIRVA
NcTob55                       ....PGNPVEAATAKIDKAAAI......................VHQKAAAVVTEPKEPQP........
```

iii.

```
                            250                                                   260
                            .                                                     .
AtOEP80a                      N..........................................ANV.LPSKFIHEAFR............
AlOEP80a                      N..........................................ANV.LPSKFIQEAFR............
GmOEP80a                      G..........................................ANV.LPAKFLEDSMR............
MtOEP80a                      G..........................................ANV.IPAKFLENSFR............
PsOEP80                       G..........................................ANV.IPAKFFRELFR............
SlOEP80a                      G..........................................ASV.LPARFIEDSFR............
HvOEP80a                      G..........................................ANM.LPSKFLDDSFR............
SbOEP80a                      G..........................................ANM.LPSKFLEDAFR............
PtOEP80a                      G..........................................ASV.LPTKFLQDAFR............
AcOEP80a                      G..........................................ANV.LPSKFLEDSFR............
BdOEP80a                      G..........................................ANM.LPSKFLDDSFR............
OsOEP80a                      G..........................................ANM.LPSKFLEDAFH............
ZmOEP80a                      G..........................................ANL.LPSKFLEDAFC............
PpOEP80                       G..........................................ANV.LPTRVVQNAFR............
SmOEP80a                      G..........................................ANV.LPTKVIEDAFR............
SmOEP80b                      G..........................................ANV.LPTKVIEDAFR............
CrOEP80                       G..........................................ASL.LPTSVIQEAFE............
VcOEP80                       G..........................................ASL.LPACVVQQAFEG.LGNHHH....
MpOEP80                       G..........................................ANV.LPTRVVQDAFH............
PgOEP80                       G..........................................ANV.LPTRIVEDAFR............
PtaOEP80                      G..........................................ANV.LPTRIVEDAFR............
PmOEP80                       G..........................................ANV.LPTRIVEDAFR............
AtOEP80b                      ......................................................................
AlOEP80b                      ......................................................................
AtOEP80c                      ......................................................................
GmOEP80b                      ......................................................................
PtOEP80b                      ......................................................................
MtOEP80b                      ......................................................................
psOEP80tr                     ......................................................................
SlOEP80b                      ......................................................................
BdOEP80b                      ......................................................................
SbOEP80b                      ......................................................................
ZmOEP80b                      ......................................................................
OsOEP80b                      ......................................................................
HvOEP80b                      ......................................................................
AcOEP80b                      ......................................................................
NmiOEP80                      G..........................................GNV.LPSRVVEDAFR............
CsOEP80                       G..........................................ANV.LPQREIEEAFR............
MpuOEP80                      G..........................................CDE.YPTRLLHQAFK............
OlOEP80                       G..........................................LEA.VPAKVVEETFR............
AtrOEP80a                     E..........................................ANV.LPTKFLEDAFR............
AtrOEP80b                     ......................................................................
Gloeobacter                   G..........................................STL.LPEAVVQETFK............
Synechocystis6803             TVPETAEG..................................KERI.LPQEVVDETFG............
Cyanothece8802                TGTDTEK....................................QRV.LPPEVIQEIFG............
Cyanothece51142               TVPDIED....................................QRA.LPPEKVEEIFG............
Oscillatoria                  G..........................................QEV.LPQTVIDEAFG............
Microcoleus                   G..........................................QQV.LPQSVIDESFR............
Microcystis                   IAPETMD....................................KSI.LPAGVVEEIFA............
Nostoc73102C                  ANPGTGV....................................PSV.LPPTTVDEVFK............
Anabaena_variabilis           ANPGTNV....................................PSV.LPQATADEIFR............
Trichodesmium101              G..........................................AEV.FPESETERIFN............
Thermosynechococcus           GN..........................................QV.LTQEKVNEIFA............
Synechococcus8102             PV.........................................SEE.LPEAVVEEIFS............
Synechococcus8109             GD.........................................EENLLSDELIEETFA............
Nostoc7107                    ANPGTGV....................................ASV.LPASTADEVFK............
Gloeocapsa                    ANPGTNV....................................PSV.LPAEVVNNIFQ............
Nostoc7120a                   ......................................................................
Nostoc7120b                   G..........................................AKA.LTYSVAQPRFQ............
Nostoc7120c                   ANPGTNV....................................PSV.LPQATADEIFR............
Prochlorococcus_marinus_1375  PS.........................................NSL.LTQSIIRDIFK............
Prochlorococcus_marinus_1986  PK.........................................NTI.IPNEFVDNVFT............
Prochlorococcus_marinus_9313  PP.........................................DVG.LSESVIEETFL............
Nostoc73102d                  ANPGTGV....................................ASV.LPANTVDEVFR............
Nostoc73102b                  G..........................................AKV.LTYQVAQQKLQ............
Nostoc73102a                  ...............................................TPEATIH................
Synechococcus_sp._JA-3-3Ab    G..........................................AKV.LDPEVLKKAFA............
Synechococcus_sp._JA-2-3B’a   G..........................................AKV.LDPEVLRAAFA............
alToc75c                      NVGLMTQPKPV.KID.SDMTEKEMIEYLRSQEKEYKQRIKKARPCL.LPGSIQREAMLM...........
alToc75a                      NVGLMVQSKPI.EMD.SDMTDKEKLEYYRSLEKDYKRRIDRARPCL.LPAPVYGEVMQM...........
alToc75b                      ......................................................................
atToc75a                      NVGLMVQSKPI.EMD.SDMTDKEKLEYYRSLEKDYKRRIDRARPCL.LPAPVYGEVMQM...........
psToc75                       NVGLMGQSKPV.EMD.PDMSEKEKIEFFRRQEREYKRRISSARPCL.LPTSVHEEIKDM...........
acToc75                       NVGLLPQSKQI.EMD.PDMTDKEKMEYFRSQEKDYRRRIEKARPCL.LPAPVHAEILHM...........
atToc75b                      ......................................................................
atToc75c                      .......................MIEYLGSQEKEYKQRIEKARPSR.GNAYAKR................
vcToc75                       NA....................................IDIKGASL.MPAEEMERICTECL.........
smToc75                       NVGLLAQTKQP.DFN..SMTEKQQQEYLGKQEEEYKKRVRKARKCM.LPRRIEQEISGW...........
ppToc75a                      NVGLLAQSRGP.EGD.EKMTDQQREELAKSQEEEYKNRLRKARPCM.LPKNVEREISGW...........
ppToc75b                      NVGLMAQSRGP.ESE.EKLTDKQREELAKSQEEEYKNRLRKSRPCM.LPKSVEREISGW...........
ppToc75c                      NVGLLSQSKGP.EGD.EKMTDQQREELAKSQEEEYKNRLRQARPCM.LPKSVEREISSW...........
zmToc75                       NVGLMSQSGQV.DFD.QDMTEREKMDYLRKQERDYQQRVRGAKPCI.LPENVRGEVLGM...........
bdToc75a                      NVGLMAQSGQA.DFD.QDMTEREKMDYLRKQERDYQQRVRGAMPCI.LPESVRGEVLAM...........
osToc75a                      NVGLMSQSGQV.DFD.QDMTEREKMDYLRKQERDYQQRVRGAKPCI.LPDNVRGEVLGM...........
osToc75b                      ..........................................................M...........
crToc75                       NA....................................IDIKGAAL.MPASEVERICNECL.........
bdToc75b                      N.........................RQRQFESEYERRLGRAAPCV.LPERVRAEVAEM...........
GsToc75                       ..............D............................SRV.LNRDVVRRTVE............
CmToc75                       ............PVD............................RLL.LPRDRLEQAVNRILQAN......
mpToc75                       NVGLLSQSRMP.DSE.ASMTDQQREEMAKIQEEEYKTRLRRARPCM.LPKIVEREISGW...........
pgToc75                       ......................................................................
ptaToc75                      NVGLLAQPKPK.EVDFNSMSDSQKERFIRDQELEYRRRIRDSRSCI.LPKSVEREIQKW...........
pmToc75                       NVGLLVQPKPR.EVDFNSMSDSQKERFIREQEQEYRRRIRESRSCI.LPKSVEREIQRW...........
atrToc75                      NVGLLPQSKPV.DMD.PDMTDREKFEYIKRQEAEYRKRMERSRPCL.LPIRVQKEVLAM...........
MtToc75                       NVGLMGQSKPI.EMD.PDMSEKEKIEFFRRQEREYKRRIDTSRPCL.LAPEVYKEIRGM...........
GmToc75                       NVGLMQQTKPV.EMD.ADMTDKERLEYYLSQEREYKRRIERARPCL.LPRYVHNEILDM...........
ptToc75                       NVGLMQQSKPI.EMD.PDMTDKEKLEYYRSQEKDYRRRIEKARPCL.LPTQVHREVLQM...........
slToc75                       NVGLMPQSKPI.EMD.ADMTEKEKLEYFNSQEQDYRRRIERSRPCL.LPVSVQREILQL...........
hvToc75                       NVGLMAQSGQA.DFD.QDMTEREKMDYLRKQERDYQQRVRGAMPCI.LPESVRGEVLAM...........
sbToc75                       NVGLMSQSGQV.DFD.QDMTEREKMDYLRKQERDYQQRVRGAKPCI.LPENVRGEVLGM...........
NmiToc75                      DAP..........................................H.FPKELKKQIWKD...........
mpuToc75                      GAT..........................................A.LPPEIEKEVLTQ...........
csToc75                       ..........................................KGSLLIPTEEVARVMNE...........
olToc75                       ...............................................LPKDVEQEVLKA...........
EcBamA                        GNKSVKD.......D....MLKQNLEASGVRVGES............LDRTTIADIEKG.LEDFYYSVGK
EcTamA                        AKVTPGVPVLIGGTD............................VVLRGGARTDKDYLKL.LDTR......
NmOMP85                       GAKMLQN.......D....AIKKNLESFGLAQSQY............FNQATLNQAVAG.LKEEYLGRGK
BpFhaC                        ............AVD.LDFGVE.....................GRLFDPAPLVQDYLN............
ScSam50                       GVVTTGT.......DH...............................IDPSVLQAYLD............
NcTob55                       ..................................YQ...........VPNPIIEDHLL............
```

```
                                                                                                 270
AtOEP80a                      .............................................................DGFGKVINI
AlOEP80a                      .............................................................DGFGKVINI
GmOEP80a                      .............................................................DGYGKIINL
MtOEP80a                      .............................................................NGYGKVINL
PsOEP80                       .............................................................NGHGKVINI
SlOEP80a                      .............................................................DGYGKIVNI
HvOEP80a                      .............................................................DRHGKIINI
SbOEP80a                      .............................................................DRHGKIINI
PtOEP80a                      .............................................................GGYGKVVNI
AcOEP80a                      .............................................................DAYGKIVNI
BdOEP80a                      .............................................................DRHGKIINI
OsOEP80a                      .............................................................DRHGKIINI
ZmOEP80a                      .............................................................VRHGKIINI
PpOEP80                       .............................................................AEFGKVVNI
SmOEP80a                      .............................................................NEYGKVVNI
SmOEP80b                      .............................................................NEYGKVVNI
CrOEP80                       .............................................................GMPGRTLNL
VcOEP80                       ..................................HHLANA..HDESEMRCRAL.......YGMPGRTLNL
MpOEP80                       .............................................................DEYGKVVNI
PgOEP80                       .............................................................DEYGKVVNY
PtaOEP80                      .............................................................DEYGKVVNY
PmOEP80                       .............................................................DEYGKIVNY
AtOEP80b                      ......................................................................
AlOEP80b                      ......................................................................
AtOEP80c                      ......................................................................
GmOEP80b                      ......................................................................
PtOEP80b                      ......................................................................
MtOEP80b                      ......................................................................
psOEP80tr                     ......................................................................
SlOEP80b                      ......................................................................
BdOEP80b                      ......................................................................
SbOEP80b                      ......................................................................
ZmOEP80b                      ......................................................................
OsOEP80b                      ......................................................................
HvOEP80b                      ......................................................................
AcOEP80b                      ......................................................................
NmiOEP80                      .............................................................EEFGKVVNI
CsOEP80                       .............................................................DQAGRTLNF
MpuOEP80                      .............................................................PQFGKVMNT
OlOEP80                       .............................................................PMMGKPINS
AtrOEP80a                     .............................................................DGYGKVVNI
AtrOEP80b                     ......................................................................
Gloeobacter                   .............................................................NQVGQTLNF
Synechocystis6803             .............................................................EQYGKILNL
Cyanothece8802                .............................................................EQYNKILNL
Cyanothece51142               .............................................................DQYGETLNL
Oscillatoria                  .............................................................DQYGKILNL
Microcoleus                   .............................................................DQYGKILNL
Microcystis                   .............................................................SQYGKTLNL
Nostoc73102C                  .............................................................EQYGKILNL
Anabaena_variabilis           .............................................................PQYGKILNL
Trichodesmium101              .............................................................EQYGETLNL
Thermosynechococcus           .............................................................PQIGRTLNL
Synechococcus8102             .............................................................PDYGRTLNL
Synechococcus8109             .............................................................SDYGRTLNL
Nostoc7107                    .............................................................DQYGKILNL
Gloeocapsa                    .............................................................PQYGRILNL
Nostoc7120a                   ......................................................................
Nostoc7120b                   .............................................................SQIGKPISP
Nostoc7120c                   .............................................................AQYGKILNL
Prochlorococcus_marinus_1375  .............................................................TDFGKTLNL
Prochlorococcus_marinus_1986  .............................................................KYYGTTLNL
Prochlorococcus_marinus_9313  .............................................................PDYGRTLNL
Nostoc73102d                  .............................................................EQYGKILNL
Nostoc73102b                  .............................................................SQIGTSISP
Nostoc73102a                  ......................................................................
Synechococcus_sp._JA-3-3Ab    .............................................................DQEGRVLNF
Synechococcus_sp._JA-2-3B’a   .............................................................DQEGRVLNF
alToc75c                      .............................................................LRDQRSLSA
alToc75a                      .............................................................LRDQGKVSA
alToc75b                      ......................................................................
atToc75a                      .............................................................LRDQGKVSA
psToc75                       .............................................................LAEQGRVSA
acToc75                       .............................................................LRERGKVSA
atToc75b                      ......................................................................
atToc75c                      ................................................................SRSLSA
vcToc75                       .............................................................PPQPYMVDI
smToc75                       .............................................................LSAESRVTA
ppToc75a                      .............................................................LRGETRVTA
ppToc75b                      .............................................................LRSETRVTA
ppToc75c                      .............................................................LRGETRVTA
zmToc75                       .............................................................MKKQEKVSA
bdToc75a                      .............................................................MKKQEKVSA
osToc75a                      .............................................................MKKQEKVSA
osToc75b                      .............................................................VKRQRKVSS
crToc75                       .............................................................PKQPYMVDI
bdToc75b                      .............................................................VRRQGKVTA
GsToc75                       .............................................................PLYGKKLDP
CmToc75                       .............................................................QNADASIDY
mpToc75                       .............................................................LRTETRVTA
pgToc75                       ..................................................................RVTA
ptaToc75                      .............................................................LLNEGRVTA
pmToc75                       .............................................................LRVEGRVTA
atrToc75                      .............................................................LREQGKVSA
MtToc75                       .............................................................LSKQGRVSA
GmToc75                       .............................................................LKRHGMVSA
ptToc75                       .............................................................LREQGKVSA
slToc75                       .............................................................LREKGTVSA
hvToc75                       .............................................................MKKQEKVSA
sbToc75                       .............................................................MKKQEKVSA
NmiToc75                      .............................................................IKIDKVCTV
mpuToc75                      .............................................................VRKQSSCTV
csToc75                       ................V............................................AVQPGPTGL
olToc75                       .............................................................ARKSQNCTV
EcBamA                        YSASVKAVVTPLPRNRVDLKLVFQEGVSAEIQQINIVGNHAFTTDELISHFQLRDEVPWWNVVGDRKYQK
EcTamA                        .............................................................PAIGTVLNQ
NmOMP85                       LNIQITPKVTKLARNRVDIDITIDEGKSAKITDIEFEGNQVYSDRKLMRQMSLTEGGIWTWLTRSNQFNE
BpFhaC                        .................................................................RPLDN
ScSam50                       .............................................................DTIMKSITL
NcTob55                       ......................................................................
```

```
                                                 280          290           300          310          320
AtOEP80a                         KRLEEAITSINGWYME.RG.LFGIV.....SDIDTLSGGIVRLQVAEA...EVNNISIRF..........
AlOEP80a                         KRLEEAITSINGWYME.RG.LFGIV.....SDIDTLSGGIVRLQVAEA...EVNNISIRF..........
GmOEP80a                         RRLDEAISSINNWYME.RG.LFAMV.....SAVEILSGGILRLQVSEA...EVDNISIRF..........
MtOEP80a                         RRLDEAISSINDWYME.RG.LFAMV.....SAVEILSGGILRLQVSEA...EVNNISIRF..........
PsOEP80                          RRLDETISSINDWYME.RG.LFAMV.....SAVEILSGGILKLHVSEA...EVNNISIRF..........
SlOEP80a                         KRLDEIISSINGWYME.RG.LFGAV.....SGIEMLSGGMIRLEVSEA...EVNNITIRF..........
HvOEP80a                         RHLDQVIKSINGWYQE.RG.LTGMV.....SYAEILSGGILRLQVSEA...EVNNINIRF..........
SbOEP80a                         RHLDKVIKSVNGWYQE.RG.LTGLV.....SYAEILSGGILRLQVSEA...EVNNISIRF..........
PtOEP80a                         KQLDEVISSINSWYME.RG.LFGMV.....SNAEILSGGIIRLQIAEA...EVNDISIRF..........
AcOEP80a                         RRLNEVIHSIDGWYRE.RG.LFGLV.....SDLEILSNGIIRLQVSEA...EVNNITIRF..........
BdOEP80a                         RHLDQVIKSVNGWYQE.RG.LTGMV.....SYAEILSGGILRLQVSEA...EVNNINIRF..........
OsOEP80a                         RHLDQVIKSVNGWYQE.RG.LTGLV.....SYAEILSGGILRLQVSEA...EVNNINIRF..........
ZmOEP80a                         RHLDQVIKSVNGWYQE.RG.LTGLV.....SYAEILSGGILRLEVSEA...EVNNISIRF..........
PpOEP80                          QRLNTVLDTLNGWYRA.RG.LFGQV.....TDVEILQDGVIKVQVAEA...EVHNINIRF..........
SmOEP80a                         QRLNTVLDTLNGWYRD.RG.YFAQV.....TDLEIMPDGILKVQFAEA...VVNNINIRF..........
SmOEP80b                         QRLNTVLDTLNGWYRD.RG.YFAQV.....TDLEIMPDGILKVQFAEA...VVNNINIRF..........
CrOEP80                          GSLQKAIARLDAWYHD.RG.IMGMV......SDYTFEGGQLQLQCAEA...VVGSVTLRF..........
VcOEP80                          GSLQKAIATLDGWYHD.RG.IMGMV......SDYTFEDGQLQLQCAEA...LVNSVTLRF..........
MpOEP80                          QRLNKVLDTLNGWYRD.RG.LFGQV.....TDVEILSDGIIRVQVAEA...EVHNINIRF..........
PgOEP80                          QRLNKVLNLIDNWYVE.RG.LFAKV.....ADVEMLSGGIIRLHVSEA...EVNNITIRF..........
PtaOEP80                         QRLSKVLNSIDNWYGE.RG.LFAKV.....ADVEMLSGGIIRLLVSEA...EVNNITIRF..........
PmOEP80                          QRLNKVLNLIDNWYGE.RG.LFAKV.....ADVEMLSGGIIRLHVSEA...EVNNITIRF..........
AtOEP80b                         .................RA...................................................
AlOEP80b                         .................RA...................................................
AtOEP80c                         ......................................................................
GmOEP80b                         .................KA...................................................
PtOEP80b                         .................KA...................................................
MtOEP80b                         .................KA...................................................
psOEP80tr                        .................KA...................................................
SlOEP80b                         .................KA...................................................
BdOEP80b                         .................KA...................................................
SbOEP80b                         .................KA...................................................
ZmOEP80b                         .................KA...................................................
OsOEP80b                         .................KA...................................................
HvOEP80b                         .................KA...................................................
AcOEP80b                         .................KA...................................................
NmiOEP80                         GRLNVVLDTLNGWYRD.RG.LFGQV.....TDVEILSNGIIRVQVAEA...EVNNVTIRF..........
CsOEP80                          PAFSTAVKRLNKWYED.RG.IFGQVCHS.PEDSFDDTGGVINVKLAEM...VVGNLNLRY..........
MpuOEP80                         NVLEKEVTKIRDWYEA.RK.IPAEW.....YSVVINPDGLLEIAITEP...RIGEVQIRF..........
OlOEP80                          NHVSGAVEYFKAYAESKRG.IDSIE..G.ISLLEEPSDGVLRLGFVDV...DINDVKVEID.........
AtrOEP80a                        RHLDGVINSINGWYME.RG.LFGMV.....SEIEILSGGILKLQVSEA...EVNNITIRF..........
AtrOEP80b                        ......................................................................
Gloeobacter                      GELQRGVKSLEEWYAE.RGYVLAKVA....DV.QSTPDGKVKLTVTEG...IIEDIRV............
Synechocystis6803                RELQEGIKTINEWYSN.QGYDLAQVV....GSPQVGADGQVTLVIAEG...IVENIQVRF..........
Cyanothece8802                   RELQNGIKKLNEWYSE.QGYDLAQVI....GSPKVGEDGTVTLIISEG...IIQEIEVRF..........
Cyanothece51142                  RELQEGIREINEWYAE.QGFDLAQVI....GSPEVSEDGIVTLVIAEG...VIEDVQVRF..........
Oscillatoria                     RQFEEGVKKINKWYQD.NGYVLGQVI....DAPQVSDDGNVTLQVAEG...VVEGVEVRF..........
Microcoleus                      RRFEEGVKKINAWYQE.NGYVLGQIT....DAPQVGEDGTVTLVVAEG...QIESVDVRF..........
Microcystis                      RELQAGVKKINDWYSK.NGYDLAQVI....GAPQVTPDGRVILVISEG...LIEKIQVRY..........
Nostoc73102C                     RDLQEGIKQLNKRYQD.QGYVLANVI....GAPQVAENGVVTLQVAEG...VVENIRVRF..........
Anabaena_variabilis              RDLQEGIKELTKRYQD.QGYVLANVV....GAPQVSENGVVTLQVAEG...VVENISVRF..........
Trichodesmium101                 KVFEQGVEQVDQWYQD.NGYVLGQVI....GAPQVGDDGTVTLEVAEG...KIKDIQVRF..........
Thermosynechococcus              RELQAGIEKINTFYRD.NGYILGQVV....GTPQVDPDGVVTLQVAEG...VVEQVTYRF..........
Synechococcus8102                NDLQKRMKDLQAWYAG.QGYSLARIS....GPERVSPEGVVTLKLVQG...SVAGVEVEF..........
Synechococcus8109                NDLQQRMKALQASVAG.LGYSLARVS....GPERVSQQGVVTLKLLQG...SVSGIEVKF..........
Nostoc7107                       RDLQEGIKTLTKRYQD.QGYVLANVI....GAPQVAENGVVTLQVAEG...VVENIKVRF..........
Gloeocapsa                       RQLQAGIQQLNRWYQD.NGYVLAQVV....AAPQVSADGVVTLEVAEG...VVEDIQVRF..........
Nostoc7120a                      .........IDG.YAQ.YG...........DYVGV....PETGGFSQT...IVQDIQIRF..........
Nostoc7120b                      EGLKQAVAQVNQWYAD.NGYNLARVL.....SIEPNRQGILNINVAEG...LVSDIKFRF..........
Nostoc7120c                      RDLQEGIKELTKRYQD.QGYVLANVV....GAPQVSENGVVTLQVAEG...VVENISVRF..........
Prochlorococcus_marinus_1375     NVLKLRMNKLKDWYSK.RGFSLARIS....GPNRITPKGKVQLKVQEG...TISDVEVVF..........
Prochlorococcus_marinus_1986     NELQNRINLIKKWYTE.KGYSLSRIN....GPERISGDGVIILNVEEG...IISDIEFRF..........
Prochlorococcus_marinus_9313     NELQARMKELQQWYAN.EGYSLARVT....GPTRVSPEGVVQLKVTQG...TVAGVEVQF..........
Nostoc73102d                     RDLQESIKQLNKRYQD.KGYVLANVI....GAPQVSENGVVTLQVAEG...VVENIRVRF..........
Nostoc73102b                     AGLQQAVAQINKWYAD.NGYNLARVL.....SIKPSSQGILTVNVAEG...LVSDIKFRF..........
Nostoc73102a                     .........IEGEYAK.YG...........DYIGVSLGDSQTLEISQK...IVKDIQIRF..........
Synechococcus_sp._JA-3-3Ab       GELQAAVAEIEQWYAD.KGYVLAKVT....DV.RSSEDGTLTLEIAEG...EIEDIRV............
Synechococcus_sp._JA-2-3B'a      GELQLAVAEIEQWYAD.KGYVLAKVT....DV.RSSEDGTITLEIAEG...VIEDIRV............
alToc75c                         RLLHKVKERVLKWYQD.QGYSYANVVNF.....GNLNSKELVFEVMEG...DITQLVIQF..........
alToc75a                         RLLQRIRDRVQKWYHD.EGYACAQVVNF.....GNLNTKEVVCEVVEG...DITQLVIQF..........
alToc75b                         ......................................................................
atToc75a                         RLLQRIRDRVQKWYHD.EGYACAQVVNF.....GNLNTKEVVCEVVEG...DITQLVIQF..........
psToc75                          RLLQKIRDRVQSWYHE.EGYACAQVVNF.....GNLNTREVVCEVVEG...DITKLSIQY..........
acToc75                          RLLQKIRDRVQKWYHD.EGYACAQVVNF.....GNLNTKEVVCEVVEG...DITQLVVQF..........
atToc75b                         ......................................................................
atToc75c                         RLLHKVKERVLKWYPD.QGYSFANVVSF.....GNLNSKELIFEVMEG...DITQLVIQF..........
vcToc75                          AVMDKVRNRIEQWYQS.RGLPFCYVGFF.....DGMEDGILRANVTEA...KVDNVSVRF..........
smToc75                          RMLQRIRERIQKWYHD.EGYACAQVVNF.....GNLNTNEIVCEVVEG...DITDVQIMF..........
ppToc75a                         RMLQRIRDRVQKWYHD.EGYACAQVVNF.....GNLNTNEVVCEVVEG...DITKVQIQF..........
ppToc75b                         RMLQRIRDRVQKWYHD.EGYACAQVVNF.....GNLNTNEVVCEVVEG......................
ppToc75c                         RMLQRIRDRVQKWYHD.EGYACAQVVNF.....GNLNTNEVVCEVVEG...DITKLQIQF..........
zmToc75                          RMLQKIRDHVQKWYHN.EGFVCAQVVNF.....GNLNTNEVVCEVVEG...DITKVEYQF..........
bdToc75a                         RMLQKIRDHVQKWYHN.EGFVCAQVVNF.....GNLNTSEVVCEVVEG...DITKVEYQF..........
osToc75a                         RLLQRIRDHVQKWYHN.EGFVCAQVVNF.....GNLNTSEVVCEVVEG...DITKVEYQF..........
osToc75b                         GLLKRMAGRIERWYHD.EGFHCAQVVSY....HGNLDAGEVVCEVVEG...DITKVEYQF..........
crToc75                          AVMDKVRNRIEQWYQS.RGLPFCYVGFF.....DGMDDGILRANVTEA...KIDNVSVRF..........
bdToc75b                         RLLQRVRDMVQKWYHD.EGFVCAQVVNF.....GNLDAGEVVCEVVEG...EVTGVEYQF..........
GsToc75                          VLVKDVITTLNVWYEE.NGYKFSRINKS.....GPFRNGVLSFRAIEP...RFAGIRLVY..........
CmToc75                          TAIRRSIDSLNAFYGE.HGYPLALVRRA...PDTPIKDGILVLEAYEP...LVARVLVEY..........
mpToc75                          RMLQRIRDRVQRWYHE.EGYACAQVVNF.....GNLNTNEVVCEVVEG...DITKLQIQF..........
pgToc75                          RLLQRIRDRVQKWYHD.QGYACAQVVNF.....GNLNSREIVCEVVEG...DITKVDVQF..........
ptaToc75                         RQLQRIRDRVQRWYHE.QGYACAQVVNF.....GNLNSKEIVCEVVEG...DITKVDVQF..........
pmToc75                          RLLQRIRDRVQKWYHD.QGYACAQVVNF.....GNLNSREIVCEVVEG...DITKVDVQF..........
atrToc75                         RLLQKIRDRVQKWYHD.EGYACAQVVNF.....GNLNTREVVCEVVEG...DITQLVVQF..........
MtToc75                          RLLQKIRDRVQKWYHD.EGYACAQVVNF.....GNLNTREVVCEVVEG...DITQLNIQF..........
GmToc75                          RLLQRIRDRVQKWYHD.EGYACAQVVNF.....GNLNTKEVVCEVVEG...DITQLDIQF..........
ptToc75                          RLLQKIRDRVQKWYHD.EGYACAQVVNF.....GNLNTKEVVCEVVEG...DITQLVIQY..........
slToc75                          RLLQKIRDKVQQWYHD.NGYACAQVVNF.....GNLNTKEVVCEVVEG...DITQMVIQF..........
hvToc75                          RMLQKIRDHVQKWYHN.EGFVCAQVVNF.....GNLNTSEVVCEVVEG...DITKVEYQF..........
sbToc75                          RMLQKIRDHVQKWYHN.EGFVCAQVVNF.....GNLNTNEVVCEVVEG...DITKVEYQF..........
NmiToc75                         RTLSKLKQQVEKWYHD.KGYVFAQVVVF.....DGMDTGELVAKVQEG...YISKVKMVF..........
mpuToc75                         RVLATMKNIIEGYYTS.KGLTFGTISHF.....DGMETGDVIAHVIEG...EITRVKAVF..........
csToc75                          QKLAGIKNIVEGWYQK.RGFVFGYISHY.....EGMETGDIVARVVEG...RVNKVNVVQ..........
olToc75                          RVLATAKNIVEGYYTS.KGLTFGTISHF.....DGMENGEVIAHVVEG...EITRVQAVF..........
EcBamA                           QKLAGDLETLRSYYLD.RGYARFNIDSTQVSLTPDKKGIYVTVNITEGDQYKLSGVEVSGNLAGH.SAEI
EcTamA                           GDYENFKKSLTSIALR.KGYFDSEFTKAQLG.........IALGLHKA......................
NmOMP85                          QKFAQDMEKVTDFYQN.NGYFDFRILDTDIQTNEDKTKQTIKITVHEGGRFRWGKVSIEGDTNEVPKAEL
BpFhaC                           EQLFLLVKALSAALYD.RGYATSIVT....FVPPGVVDGVLKLKVEWG...RIKG...............
ScSam50                          GQLVKNADVLNKRLCQ......................HHIALNAKQS.........FHF..........
NcTob55                          .............................................TPA...TVNSIEIHGANNT......
```

	330
AtOEP80a	....LDRK.............TGEPTK....
AlOEP80a	....LDRK.............TGEPTK....
GmOEP80a	....LDRK.............TGETTM....
MtOEP80a	....LDRK.............TGETTV....
PsOEP80	....LDRK.............TGETTV....
SlOEP80a	....LD.K.............TGEPTV....
HvOEP80a	....LDRR.............TGEPTT....
SbOEP80a	....LDRK.............TGEPTL....
PtOEP80a	....LDRK.............TGEPTK....
AcOEP80a	....LDRK.............TGEPTT....
BdOEP80a	....LDRR.............TGEPTI....
OsOEP80a	....LDRR.............TGEPTV....
ZmOEP80a	....LDRK.............TGEPTL....
PpOEP80	....LDRK.............TGEPTV....
SmOEP80a	....LDRK.............TGEPTV....
SmOEP80b	....LDRK.............TGEPTV....
CrOEP80	....LDAA.............SGQPKA....
VcOEP80	....LDST.............SGQPTA....
MpOEP80	....LDRK.............TGEPTT....
PgOEP80	....LDRK.............TGEPTV....
PtaOEP80	....LDRK.............TGEPTV....
PmOEP80	....LDRK.............TGEPTV....
AtOEP80b	....
AlOEP80b	....
AtOEP80c	....
GmOEP80b	....
PtOEP80b	....
MtOEP80b	....
psOEP80tr	....
SlOEP80b	....
BdOEP80b	....
SbOEP80b	....
ZmOEP80b	....
OsOEP80b	....
HvOEP80b	....
AcOEP80b	....
NmiOEP80	....LDRK.............TGEPTQ....
CsOEP80	....VDRK.............TGEVRE....
MpuOEP80	....LDRK.............TGEPVV....
OlOEP80	....PSIDAK.............TGKENK....
AtrOEP80a	....LDRK.............TGEPNT....
AtrOEP80b	....
Gloeobacter	....KGNT....................
Synechocystis6803	....FDSEDE..............PVQ....
Cyanothece8802	....FNAEDD..............PVE....
Cyanothece51142	....FNAEDE..............PVD....
Oscillatoria	....LNKEGEATD.......AKGQPIK....
Microcoleus	....LNKEGEATD.......ATGQPIG....
Microcystis	....FNVEQE..............PVK....
Nostoc73102C	....RNKDGQETD.......EKGQPIR....
Anabaena_variabilis	....RNKEGQDVN.......EQGQPIR....
Trichodesmium101	....LNSEGETVD.......EEGNVIK....
Thermosynechococcus	....LNKEGE..............PTK....
Synechococcus8102	....LNSEGTSTD.......ENGDPIR....
Synechococcus8109	....LTQDGDDTD.......ENGNPIR....
Nostoc7107	....RNKEGQETD.......DQGRPIR....
Gloeocapsa	....IN.EGEATD.......DEGRPIE....
Nostoc7120a	....IDDNNQSLD.......DQGKPII....
Nostoc7120b	....VNDDGKTID.......SNGNPVG....
Nostoc7120c	....RNKEGQDVN.......EQGQPIR....
Prochlorococcus_marinus_1375	....LDEEGNSIR.......ENGKPVR....
Prochlorococcus_marinus_1986	....IGSDGESVV.......D.GKPRK....
Prochlorococcus_marinus_9313	....LNKEGDSTD.......EKGEPIN....
Nostoc73102d	....RNKDGQETD.......EKGQPIR....
Nostoc73102b	....VNDEGKTVD.......SKGNPVG....
Nostoc73102a	....IN..............DKGLPIK....
Synechococcus_sp._JA-3-3Ab	....QGNT....................
Synechococcus_sp._JA-2-3B'a	....QGNT....................
alToc75c	....QD..............KLGNVVE....
alToc75a	....QD..............KLGNVVE....
alToc75b	....
atToc75a	....QD..............KLGNVVE....
psToc75	....LD..............KLGNVVE....
acToc75	....QD..............KLGNVCE....
atToc75b	....
atToc75c	....QD..............KLGNVAE....
vcToc75	....VRPKLTGDSELEYSVHDEGRIVKAE..
smToc75	....QD..............KMGLPVE....
ppToc75a	....QD..............KMGFPSV....
ppToc75b	....
ppToc75c	....QD..............KMGFPSV....
zmToc75	....QD..............KLGNIVE....
bdToc75a	....QD..............KLGNFVE....
osToc75a	....QD..............KLGNFVE....
osToc75b	....LD..............KLGNVVD....
crToc75	....VRPKLTGDSELEYSVYDEGKVVKAD..
bdToc75b	....LD..............KLGNDVE....
GsToc75	....LD..............DNREPCN....
CmToc75	....VD..............ANQEPCS....
mpToc75	....LD..............KMGTPCE....
pgToc75	....QD..............KMGNTCE....
ptaToc75	....QD..............KMGNTCE....
pmToc75	....QD..............KMGNTCE....
atrToc75	....QD..............KLGNVCE....
MtToc75	....LD..............KLGNVVE....
GmToc75	....QD..............KLGNVVE....
ptToc75	....QD..............KLGNVVE....
slToc75	....QD..............KLGNVCE....
hvToc75	....QD..............KLGNFVE....
sbToc75	....QD..............KLGNIVE....
NmiToc75	....TD..............AGGNPMPQ...
mpuToc75	....LD..............DQMQPV..N..
csToc75	....VD..............DEGNTKKGA..
olToc75	....VD..............ENLQPMPGN..
EcBamA	EQLTKIEPGELYNGTKVTKMEDDIKKLLGRYGYAYPRVQSMPEINDADKTVKLRVNV..DAGNRFYVRKI
EcTamA	..........FWD..............................ID.............YNSGERYRFGHV
NmOMP85	EKLLTMKPGKWYERQQMTAVLGEIQNRMGSAGYAYSEISVQPLPNAETKTVDFVLHI..EPGRKIYVNEI
BpFhaC	...........WL..............................ID................GKPLE....
ScSam50	....
NcTob55	....RRGLLDHVFKPVVE......ETASPTTTL..

```
                                                                                                340
AtOEP80a                       ...G....................................................KTSPETILRQLT..
AlOEP80a                       ...G....................................................KTSPETILRQLT..
GmOEP80a                       ...G....................................................KTKPETILRQIT..
MtOEP80a                       ...G....................................................KTKPETILRQIT..
PsOEP80                        ...G....................................................KTKPETILRQIT..
SlOEP80a                       ...G....................................................KTRPETILRQLT..
HvOEP80a                       ...G....................................................KTKPETILQQLT..
SbOEP80a                       ...G....................................................KTQPETILRQLT..
PtOEP80a                       ...G....................................................KTKPETILRQLT..
AcOEP80a                       ...G....................................................KTKPETILRQLT..
BdOEP80a                       ...G....................................................KTQPDTILRQLT..
OsOEP80a                       ...G....................................................KTQPETILRHLT..
ZmOEP80a                       ...G....................................................KTQPETILRQLT..
PpOEP80                        ...G....................................................KTRPDTLLRQLT..
SmOEP80a                       ...G....................................................KTRPETLLRQLT..
SmOEP80b                       ...G....................................................KTRPETLLRQLT..
CrOEP80                        ...KT...................................................RTRPHITTRHLT..
VcOEP80                        ...SATILKPTSSASTTTTITTTSTSTGNSNSNNDGNSENGTTATKKKPPALHKPPRTKPHVVTRHLTG.
MpOEP80                        ...G....................................................KTQPETLLRQLT..
PgOEP80                        ...G....................................................KTRPETLLRQLT..
PtaOEP80                       ...G....................................................KTRPETLLRQLT..
PmOEP80                        ...G....................................................KTRPETLLRQLT..
AtOEP80b                       ......................................................................
AlOEP80b                       ......................................................................
AtOEP80c                       ......................................................................
GmOEP80b                       ......................................................................
PtOEP80b                       ......................................................................
MtOEP80b                       ......................................................................
psOEP80tr                      ......................................................................
SlOEP80b                       ......................................................................
BdOEP80b                       ......................................................................
SbOEP80b                       ......................................................................
ZmOEP80b                       ......................................................................
OsOEP80b                       ......................................................................
HvOEP80b                       ......................................................................
AcOEP80b                       ......................................................................
NmiOEP80                       ...G....................................................KTRPETLLRQLM..
CsOEP80                        ...EG...................................................ATRPEVVLRQLA..
MpuOEP80                       ...G....................................................STRPEMITRHLKS.
OlOEP80                        ...V....................................................LSKVDSVMPYFEN.
AtrOEP80a                      ...G....................................................KTRPETILQQLT..
AtrOEP80b                      ......................................................................
Gloeobacter                    ........................................................RTRDFIVTREVA..
Synechocystis6803              ...G....................................................RTRDFIITREMR..
Cyanothece8802                 ...G....................................................RTRKFIVTREMQ..
Cyanothece51142                ...G....................................................RTRDFIVTREMQ..
Oscillatoria                   ...G....................................................NTREFIITREVE..
Microcoleus                    ...G....................................................NTRQFVITREVE..
Microcystis                    ...G....................................................KTREFIITREMR..
Nostoc73102C                   ...G....................................................RTQDYIITRELE..
Anabaena_variabilis            ...G....................................................RTQDYIITREVE..
Trichodesmium101               ...G....................................................RTREYIITREIE..
Thermosynechococcus            ...Q....................................................RTRDFVISREMD..
Synechococcus8102              ...G....................................................KTKEWVITREIS..
Synechococcus8109              ...G....................................................KTREWVITREVS..
Nostoc7107                     ...G....................................................RTQEYIIKREVE..
Gloeocapsa                     ...G....................................................RTRDFIITRELA..
Nostoc7120a                    ...G....................................................RTQKDFILGLLK..
Nostoc7120b                    ...G....................................................RTKPDFLRQQLK..
Nostoc7120c                    ...G....................................................RTQDYIITREVE..
Prochlorococcus_marinus_1375   ...G....................................................KTKRWVIDRELL..
Prochlorococcus_marinus_1986   ...G....................................................KTKDWVIKRELK..
Prochlorococcus_marinus_9313   ...G....................................................KTKPWVITREIS..
Nostoc73102d                   ...G....................................................RTQAYIITRELE..
Nostoc73102b                   ...G....................................................RTKPDFLQQQLK..
Nostoc73102a                   ...G....................................................RTQKDFIIGLLR..
Synechococcus_sp._JA-3-3Ab     ........................................................RTRDFIITRELK..
Synechococcus_sp._JA-2-3B'a    ........................................................KTRDFIITRELK..
alToc75c                       ...G....................................................NTQIPVVRRQIPK.
alToc75a                       ...G....................................................NTQVPVVRRELPK.
alToc75b                       ......................................................................
atToc75a                       ...G....................................................NTQVPVVRRELPK.
psToc75                        ...G....................................................NTEGPVVQRELPK.
acToc75                        ...G....................................................NTQLPVVRRELPK.
atToc75b                       ......................................................................
atToc75c                       ......................................................................
vcToc75                        ............................................................KVIEASGFQ.
smToc75                        ...G....................................................NTQVPIIERELPS.
ppToc75a                       ...G....................................................HTQEVIIQRELPD.
ppToc75b                       ......................................................................
ppToc75c                       ...G....................................................RTQEVIIKRELPD.
zmToc75                        ...G....................................................NTQLQIIDRELPQ.
bdToc75a                       ...G....................................................NTQIPIIDRELPQ.
osToc75a                       ...G....................................................NTQIPIIDRELPQ.
osToc75b                       ...G....................................................NTSIPLIDRELPQ.
crToc75                        ............................................................KIIEASGFQ.
bdToc75b                       ...G....................................................RTRVPVIDRELPQ.
GsToc75                        ..KG....................................................RTKSDTIEKAVG..
CmToc75                        ...G....................................................RTHASVIARALGYD
mpToc75                        ...G....................................................VTKMQIIGRELPD.
pgToc75                        ...G....................................................NTQEVIIRKELPK.
ptaToc75                       ...G....................................................NTREIIIRRELPK.
pmToc75                        ...G....................................................NTQEIIIRRELPK.
atrToc75                       ...G....................................................NTELPIIRRELPK.
MtToc75                        ...G....................................................NTENPVVQRELPK.
GmToc75                        ...G....................................................NTQVPVIQRELPR.
ptToc75                        ...G....................................................NTQLPVVKRELPK.
slToc75                        ...G....................................................NTQYPVVRRELPR.
hvToc75                        ...G....................................................NTQIPIIDRELPQ.
sbToc75                        ...G....................................................NTQLPIIDRELPQ.
NmiToc75                       ..PG....................................................STKWHVVERELPM.
mpuToc75                       ..RG....................................................RTHPRVIQRE..H.
csToc75                        ...G....................................................EVQPEILRELP...
olToc75                        ..KG....................................................VTHPRVIQRE..H.
EcBamA                         RFEGND..................................................TSKDAVLRREMRQ.
EcTamA                         TFEG...................................................SQIRDEYLQNLVP..
NmOMP85                        HITGNN..................................................KTRDEVVRRELRQ.
BpFhaC                         ...G....................................................TRDRMMVFSAMP..
ScSam50                        ..QGNTYISDE............................................KETHDVVPLMEVV..
NcTob55                        ...GEA..................................................LARISTATQKLT..
```

```
                                                     350       360       370       380
AtOEP80a                      ...TK.......................KGQVYSMLQGKRDVDTVLAMGIM....EDVSIIPQPA.GDSG
AlOEP80a                      ...TK.......................KGQVYSMLQGKRDVDAVLAMGIM....EDVSIIPQPA.GDTG
GmOEP80a                      ...TK.......................KGQVYSMLEGKRDVETVLTMGIM....EDVSIIPQPA..DTG
MtOEP80a                      ...TK.......................KGQVYSMHQGKRDVETVLTMGIM....EDVSIIPQPA..DTG
PsOEP80                       ...TK.......................KGQVYSMHQGKRDVETVLTMGIM....EDVSILPQPA..DTG
SlOEP80a                      ...TK.......................KGQVYSMLQGKRDVDTVLAMGIM....EDVSIIPQPA.GDTG
HvOEP80a                      ...TK.......................KGQAYNRAQVKRDVETILTMGIM....EDVTIIPQPV.GDSN
SbOEP80a                      ...TK.......................KGQAYNRAQVKRDVETILTMGIM....EDVTIIPQPV.GDSN
PtOEP80a                      ...TK.......................KGQVYSMLQGKRDVDTVLTMGIM....EDVSFIPQPA.EDTG
AcOEP80a                      ...TK.......................KGQVYSLLQGKRDVETVLTMGIM....EDVSIIPQPA.GDTG
BdOEP80a                      ...TK.......................KGQAYNRAQVKRDVETILTMGIM....EDVTIIPQPV.GDSN
OsOEP80a                      ...TK.......................KGQAYNRAQVKRDVETILTMGIM....EDVTIIPQPV.GDSN
ZmOEP80a                      ...TR.......................KGQAYNRAQVKRDVETILTMGIM....EDVTIIPQPV.GDSS
PpOEP80                       ...TK.......................KGQVYSLQQGRRDVETVFTMGIM....EDVNLVPQPA.GDTG
SmOEP80a                      ...TK.......................KGQVYSLQQGRRDAETVFTMGIM....EDVNIVPQAA.GDTG
SmOEP80b                      ...TK.......................KGQVYSLQQGRRDAETVFTMGIM....EDVNIVPQAA.GDTG
CrOEP80                       ...TK.......................PGQVYNLRGIRRDINAVYSTGLF....EDVNVVTREA.ADST
VcOEP80                       ...TR.......................PGDVYNLRAVRRDLNAVYATGLF....EDVNVSTREA.EDST
MpOEP80                       ...TK.......................KGQVYSLQQGRRDVETVFTMGIM....EDVNLVPQPA.GDTG
PgOEP80                       ...TK.......................KGQVYSLQQGRRDVETVLTMGIM....EDVSILPQHA.GDTG
PtaOEP80                      ...TK.......................KGQVYSLQQGRRDVETVLTMGIM....EDVSIVPQHA.GDTG
PmOEP80                       ...TK.......................KGQVYSLQQGRRDVETILTMGIM....EDVSIVPQHA.GDTG
AtOEP80b                      ......................................................................
AlOEP80b                      ......................................................................
AtOEP80c                      ......................................................................
GmOEP80b                      ......................................................................
PtOEP80b                      ......................................................................
MtOEP80b                      ......................................................................
psOEP80tr                     ......................................................................
SlOEP80b                      ......................................................................
BdOEP80b                      ......................................................................
SbOEP80b                      ......................................................................
ZmOEP80b                      ......................................................................
OsOEP80b                      ......................................................................
HvOEP80b                      ......................................................................
AcOEP80b                      ......................................................................
NmiOEP80                      ...TE.......................PGQVYSLQQGRRDVETVFTMGIM....EDVNLVPQPS.GDTG
CsOEP80                       ...TR.......................PGQVYNVRQAKMDIDAVYSMGLF....DDVTILPQPA.EDST
MpuOEP80                      ...VK.......................PGKVLHARMVE.DLQDLIATAGL....ETATITWAPR.RDET
OlOEP80                       ...IK.......................TTKVLNGMRVRETIDRFRAQNPRFG.EPQINMLPKPG.....
AtrOEP80a                     ...TK.......................KGQVYSLQQVKRDVETVLAMGIM....EDVSIIPQPA.GDTG
AtrOEP80b                     ......................................................................
Gloeobacter                   ...LK.......................PGGVFNRNAIQRDLQRVYALNIF....KDVGLDLGQG.QDPQ
Synechocystis6803             ...LK.......................PGDVFNRNRAQTDLQRVYSLGLF....EDVRLSFNPG.SDPT
Cyanothece8802                ...LK.......................SGNVFNRRIAQTDLQRVFSLGLF....EDVRISFSPG.EDPK
Cyanothece51142               ...LE.......................PGDVFNRKTAQFDLQRIFGLGLF....EDVRISFSPG.EDPR
Oscillatoria                  ...LK.......................PGDVFNRNKAERDLRRVFGLGIF....EDVRLALEPGATDPR
Microcoleus                   ...LK.......................PGDIFNRTKAERDLRRVFGLGIF....EDVRLALEPGTTDPR
Microcystis                   ...LK.......................AGDIFNRNTAQQDLQRVFGLGLF....EDVRLSFSPG.SDPR
Nostoc73102C                  ...LK.......................PGQTFNRNTVQKDLQRVYGLGLF....EDVNVSLDPG.TDPS
Anabaena_variabilis           ...LK.......................PGQVFNRNTVQKDLQRVFGTGLF....EDVNVSLDPG.TDPT
Trichodesmium101              ...LK.......................TGDIFQRQTAEKDIRRVFDLGIF....EDVRLGLEPAPDDPN
Thermosynechococcus           ...TQ.......................PGVVLNQKTVQADLRRLFELGLF....EDVQVALEPG.QDPR
Synechococcus8102             ...VQ.......................PGDTFNRNKLEKDIKRLYGTQLF....SDVKVTLKPVPEQPG
Synechococcus8109             ...IQ.......................PGDPFNRNQLERDIKRLYTTQLF....SDVKVTLRPVPEEPG
Nostoc7107                    ...LK.......................PGQIFNRNTVQKDLQRVYGLGLF....EDVNVSLDPG.TDPS
Gloeocapsa                    ...LQ.......................PGQVFNRTVVQQDLQRVFGLGLF....EDVNVSLNPG.QDPR
Nostoc7120a                   ...LK.......................PGQIFREEALQADLGRLQKLESF....TYVRAYRE...ENPF
Nostoc7120b                   ...LQ.......................PGQVFQENIVKQDVQQLYRTGLF....QSVNVAFA...GDAT
Nostoc7120c                   ...LK.......................PGQVFNRNTVQKDLQRVFGTGLF....EDVNVSLDPG.TDPT
Prochlorococcus_marinus_1375  ...SR.......................PGLTFNRNDLESDIKRLYGLSLF....SDIKVTLKPIVGEPG
Prochlorococcus_marinus_1986  ...TI.......................PGSIFNRTILEADIKRLYATSLF....DDVKVSLGPDIKNPG
Prochlorococcus_marinus_9313  ...IK.......................PGEVFNRNQLEADIKRLYGTSLF....SDVKVTLKPVAGEPG
Nostoc73102d                  ...LK.......................PGQIFNRNTVQKDLQRVYGLGLF....EDVNVSLDPG.TDPS
Nostoc73102b                  ...LK.......................PGQIFQENVVKQDIQQLYRTGLF....EQVNVALE...GDAT
Nostoc73102a                  ...LK.......................PGQVFREDLLQADLQRLRRLESF....NQVNVYPK...EDAS
Synechococcus_sp._JA-3-3Ab    ...TK.......................PGDVFNRNQIQKDLQAVFDLNLF....QDVNVGLNPG.QDPN
Synechococcus_sp._JA-2-3B'a   ...SK.......................PGDVFNRNQIQEDLQAVFNLNLF....QDVNVGLNPG.QDPD
alToc75c                      ..QLR.......................PGHVLNMKVANEAVRNIHSLGLF....SNIEIMPVPDEKKEG
alToc75a                      ..QLR.......................QGYVFNIEAGKQALRNINSLGLF....SNIEVNPRPDEKNEG
alToc75b                      ..................................MEAVNEAVRKIKSL...........VIPHPDEK.TN
atToc75a                      ..QLR.......................QGYVFNIEAGKKALSNINSLGLF....SNIEVNPRPDEKNEG
psToc75                       ..QLL.......................PGHTFNIEAGKQALRNINSLALF....SNIEVNPRPDEMNEG
acToc75                       ..QLQ.......................QGHVFNIEAGKLALRNINSLALF....SNIEVNPRPDEKNEG
atToc75b                      ..................................MEAVKEAVRKIKSL...........VIPHADEK.DN
atToc75c                      .............................GHVLNIKVVHEAVRNIHSLGLF....SNIEIMPIPNEKREG
vcToc75                       ..HLHSF....................VKGNHYHVEDGYDAMNSIFSCGLL....EDINIEPEQDPQDVN
smToc75                       ..QLS.......................SGKIFNIEAGKKALRNINNLSLF....SNIEVNPRPDEEIEG
ppToc75a                      ..QLR.......................SGQIFNIEAGKKALRNVNALALF....SNIEVNPRPDEENDG
ppToc75b                      .........................................................IEVNPRPDEENDG
ppToc75c                      ..QLK.......................AGNIFNIEAGKKALRNVNALALF....SNIEVNPRPDEDNDG
zmToc75                       ..QLR.......................PGHIFNIGAGKQALKNINSLALF....SNIEVNPRPDETKEG
bdToc75a                      ..QLR.......................PGHIFNIGAGKQALKNINSLALF....SNIEVNPRPDETKEG
osToc75a                      ..QLR.......................PGHIFNIGAGKQALKNINSLALF....SNIEVNPRPDETKEG
osToc75b                      ..QLR.......................PGHIYNNGAGKQALKNIDSLGLF....STIEVQPRPDETNQG
crToc75                       ............................RGHHYHVEDGYDAMNSIFACGLL....EDINIEPEQDPSDVN
bdToc75b                      ..QLR.......................PGHIFSIEAGKQAQKNIHSLGVF....SNIE..........
GsToc75                       ...IQ.......................RGQVFRWS..DRYWERITELGLFDYVRAELEL......RDDQ
CmToc75                       GLGVE..KEMPDHQRHSRKSRSAQKACPRKVPFRWT..EQHFQRLQQLGLFESVTADVRVIDAVTPHDSP
mpToc75                       ..QLR.......................PGQVFNIEAGKKALRNVNGLALF....SNIEVNPRPDEESDG
pgToc75                       ..QLR.......................RGHIFNIEAGKQALRNVNSLALF....SNIEVNPRPDEKSEG
ptaToc75                      ..QLR.......................RGHIFNIEAGKQALRNVNSLALF....SNIEVNPRPDEKNEG
pmToc75                       ..QLR.......................RGHIFNIEAGKQALRNVNSLALF....SNIEVNPRPDEKSEG
atrToc75                      ..QLR.......................QGHVFNIEAGKQALRNINSLALF....SNIEVNPRPDEKNEG
MtToc75                       ..QLL.......................PGHTFNIEAGKQALRNINSLALF....SNIEVNPRPDEKNEG
GmToc75                       ..QLR.......................PGYTFNIEAGKQALRNVNSLALF....SNIEVNPRPDETNEG
ptToc75                       ..QLR.......................QGQVFNIEAGKQALRNINSLALF....SNIEVNPRPDEKNEG
slToc75                       ..QLR.......................QGKVFNIEAGKQALRNINSLALF....SNIEVNPRPDEKNEG
hvToc75                       ..QLR.......................PGHIFNIGAGKQALKNINSLALF....SNIEVNPRPDETKEG
sbToc75                       ..QLR.......................PGHIFNIGAGKQALKNINSLALF....SNIEVNPRPDETKEG
NmiToc75                      ..GLQ.......................RGMMYNIEDGRKALRDVFMLQLF....ENVQVVPKPDEGEEG
mpuToc75                      ..NFQ.......................VGKLYNVEDAKTALRDIFLLQLF....DNVQVVPRPDERDQS
csToc75                       ...FK.......................PGQLYNAEDGRKALRDVFALGLF....DNVQIFPRPNPKDEA
olToc75                       ..NFK.......................VGKLYNVEDAKTALRDIFLLQLF....DNVQVVPRPDERDPS
EcBamA                        .........M..................EGAWLGSDLVDQGKERLNRLGFF....ETVDTDTQRVPGSPD
EcTamA                        ...FK.......................EGDEYESKDLAELNRRLSATGWF....NSVVVAPQFDKARET
NmOMP85                       .........M..................ESAPYDTSKLQRSKERVELLGYF....DNVQFDAVPLAGTPD
BpFhaC                        .......GW...................QDKVLNVFDIDQAIYNINNGGKT....GNITIVPADE.....
ScSam50                       .......................................................SQLDILPPKTFTAKT
NcTob55                       ............................RFGIFKED..........GFGVFI...SDARQQQQEQFQSPT
```

ii.

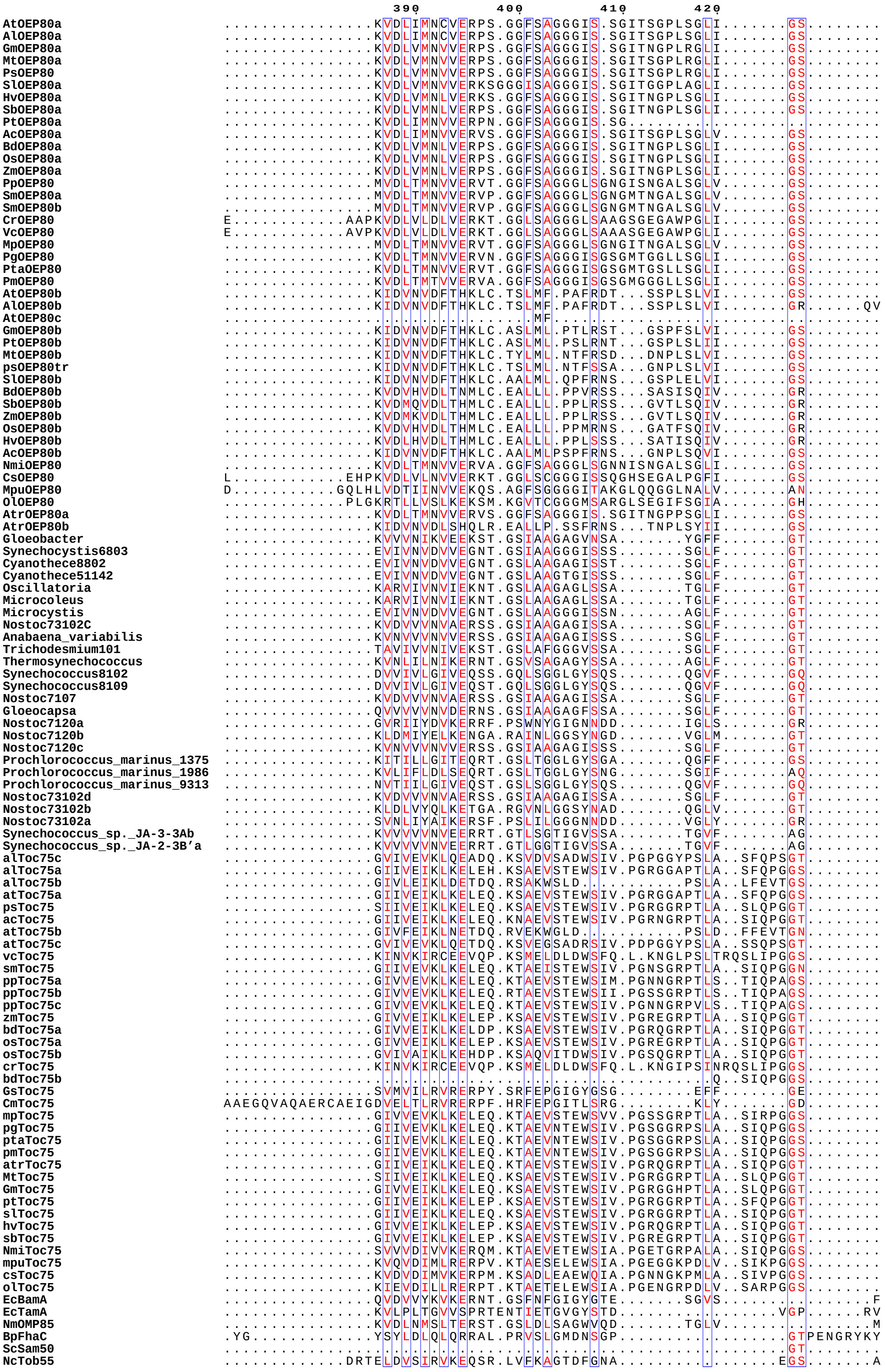

390 400 410 420
AtOEP80a ...............KVDLIMNCVERPS.GGFSAGGGIS.SGITSGPLSGLI.......GS........
AlOEP80a ...............KVDLIMNCVERPS.GGFSAGGGIS.SGITSGPLSGLI.......GS........
GmOEP80a ...............KVDLVMNVVERPS.GGFSAGGGIS.SGITNGPLRGLI.......GS........
MtOEP80a ...............KVDLVMNVVERPS.GGFSAGGGIS.SGITSGPLRGLI.......GS........
PsOEP80 ...............KVDLVMNVVERPS.GGFSAGGGIS.SGITSGPLRGLI.......GS........
SlOEP80a ...............KVDLVMNVVERKSGGGISAGGGIS.SGITGGPLAGLI.......GS........
HvOEP80a ...............KVDLVMNLVERKS.GGFSAGGGIS.SGITNGPLSGLI.......GS........
SbOEP80a ...............KVDLVMNLVERPS.GGFSAGGGIS.SGITNGPLSGLI.......GS........
PtOEP80a ...............KVDLIMNVVERPN.GGFSAGGGIS.SG...........................
AcOEP80a ...............KVDLIMNVVERVS.GGFSAGGGIS.SGITSGPLSGLV.......GS........
BdOEP80a ...............KVDLVMNLVERPS.GGFSAGGGIS.SGITNGPLSGLI.......GS........
OsOEP80a ...............KVDLVMNLVERPS.GGFSAGGGIS.SGITNGPLSGLI.......GS........
ZmOEP80a ...............KVDLVMNLVERPS.GGFSAGGGIS.SGITNGPLSGLI.......GS........
PpOEP80 ...............MVDLTMNVVERVT.GGFSAGGGLSGNGISNGALSGLV.......GS........
SmOEP80a ...............MVDLTMNVVERVP.GGFSAGGGLSGNGMTNGALSGLV.......GS........
SmOEP80b ...............MVDLTMNVVERVP.GGFSAGGGLSGNGMTNGALSGLV.......GS........
CrOEP80 E...........AAPKVDLVLDLVERKT.GGLSAGGGLSAAGSGEGAWPGLI.......GS........
VcOEP80 E...........AVPKVDLVLDLVERKT.GGLSAGGGLSAAASGEGAWPGLI.......GS........
MpOEP80 ...............MVDLTMNVVERVT.GGFSAGGGLSGNGITNGALSGLV.......GS........
PgOEP80 ...............KVDLTMNVVERVN.GGFSAGGGISGSGMTGGLLSGLI.......GS........
PtaOEP80 ...............KVDLTMNVVERVT.GGFSAGGGISGSGMTGSLLSGLI.......GS........
PmOEP80 ...............KVDLTMTVVERVA.GGFSAGGGISGSGMGGGLLSGLI.......GS........
AtOEP80b ...............KIDVNVDFTHKLC.TSLMF.PAFRDT...SSPLSLVI.......GS.........
AlOEP80b ...............KIDVNVDFTHKLC.TSLMF.PAFRDT...SSPLSLVI.......GR......QV
AtOEP80c ....MF...
GmOEP80b ...............KIDVNVDFTHKLC.ASLML.PTLRST...GSPFSLVI.......GS........
PtOEP80b ...............KIDVNVDFTHKLC.ASLML.PSLRNT...GSPLSLII.......GS........
MtOEP80b ...............KIDVNVDFTHKLC.TYLML.NTFRSD...DNPLSLVI.......GS........
psOEP80tr ...............KIDVNVDFTHKLC.TSLML.NTFSSA...GNPLSLVI.......GS........
SlOEP80b ...............KIDVNVDFTHKLC.AALML.QPFRNS...GSPLELVI.......GS........
BdOEP80b ...............KVDVHVDLTNMLC.EALLL.PPVRSS...SASISQIV.......GR........
SbOEP80b ...............KVDMQVDLTHMLC.EALLL.PPLRSS...GVTLSQIV.......GR........
ZmOEP80b ...............KVDMKVDLTHMLC.EALLL.PPLRSS...GVTLSQIV.......GR........
OsOEP80b ...............KVDVHVDLTHMLC.EALLL.PPMRNS...GATFSQIV.......GR........
HvOEP80b ...............KVDLHVDLTHMLC.EALLL.PPLSSS...SATISQIV.......GR........
AcOEP80b ...............KIDVNVDFTHKLC.AALMLPSPFRNS...GNPLSQVI.......GS........
NmiOEP80 ...............KVDLTMNVVERVA.GGFSAGGGLSGNNISNGALSGLI.......GS........
CsOEP80 L...........EHPKVDLVLNVVERKT.GGLSCGGGISSQGHSEGALPGFI.......GS........
MpuOEP80 D..........GQLHLVDTIINVVEKQS.AGFSGGGGITAKGLQQGGLNALV.......AN........
OlOEP80 ............PLGKRTLLVSLKEKSM.KGVTCGGGMSARGLSEGIFSGIA.......GH........
AtrOEP80a ...............KVDLTMNVVERVS.GGFSAGGGIS.SGITNGPPSGLI.......GS........
AtrOEP80b ...............KIDVNVDLSHQLR.EALLP.SSFRNS...TNPLSYII.......GS........
Gloeobacter ...............KVVVNIKVEEKST.GSIAAGAGVNSA......YGFF.......GT........
Synechocystis6803 ...............EVIVNVDVVEGNT.GSIAAGGGISSS......SGLF.......GT........
Cyanothece8802 ...............EVIVNVDVVEGNT.GSLAAGAGISST......SGLF.......GT........
Cyanothece51142 ...............EVIVNVDVVEGNT.GSLAAGTGISSS......SGLF.......GT........
Oscillatoria ...............KARVIVNVIEKNT.GSLAAGAGLSSA......TGLF.......GT........
Microcoleus ...............KARVIVNVIEKNT.GSLAAGAGLSSA......TGLF.......GT........
Microcystis ...............EVIVNVDVVEGNT.GSLAAGGGISSN......AGLF.......GT........
Nostoc73102C ...............KVDVVVNVAERSS.GSIAAGAGISSA......SGLF.......GT........
Anabaena_variabilis ...............KVNVVVNVVERSS.GSIAAGAGISSS......SGLF.......GT........
Trichodesmium101 ...............TAVIVVNIVEKST.GSLAFGGGVSSA......SGLF.......GT........
Thermosynechococcus ...............KVNLILNIKERNT.GSVSAGAGYSSA......AGLF.......GT........
Synechococcus8102 ...............DVVIVLGIVEQSS.GQLSGGGLGYSQS......QGVF.......GQ........
Synechococcus8109 ...............DVVIVLGIVEQST.GQLSGGGLGYSQS......QGVF.......GQ........
Nostoc7107 ...............KVDVVVNVAERSS.GSIAAGAGISSA......SGLF.......GT........
Gloeocapsa ...............QVVVVVNVDERNS.GSIAAGAGFSSA......SGLF.......GT........
Nostoc7120a ...............GVRIIYDVKERRF.PSWNYGIGNNDD......IGLS.......GR........
Nostoc7120b ...............KLDMIYELKENGA.RAINLGGSYNGD......VGLM.......GT........
Nostoc7120c ...............KVNVVVNVVERSS.GSIAAGAGISSS......SGLF.......GT........
Prochlorococcus_marinus_1375 ...............KITILLGITEQRT.GSLTGGLGYSGA......QGFF.......GS........
Prochlorococcus_marinus_1986 ...............KVLIFLDLSEQRT.GSLTGGLGYSNG......SGIF.......AQ........
Prochlorococcus_marinus_9313 ...............NVTIILGIVEQST.GSLSGGLGYSQS......QGVF.......GQ........
Nostoc73102d ...............KVDVVVNVAERSS.GSIAAGAGISSA......SGLF.......GT........
Nostoc73102b ...............KLDLVYQLKETGA.RGVNLGGSYNAD......QGLV.......GT........
Nostoc73102a ...............SVNLIYAIKERSF.PSLILGGGNNDD......VGLY.......GR........
Synechococcus_sp._JA-3-3Ab ...............KVVVVVNVEERRT.GTLSGTIGVSSA......TGVF.......AG........
Synechococcus_sp._JA-2-3B'a ...............KVVVVVNVEERRT.GTLGGTIGVSSA......TGVF.......AG........
alToc75c ...............GVIVEVKLQEADQ.KSVDVSADWSIV.PGPGGYPSLA..SFQPSGT........
alToc75a ...............GIIVEIKLKELEH.KSAEVSTEWSIV.PGRGGAPTLA..SFQPGGS........
alToc75b ...............GIVLEIKLDETDQ.RSAKWSLD..........PSLA..LFEVTGS........
atToc75a ...............GIIVEIKLKELEQ.KSAEVSTEWSIV.PGRGGAPTLA..SFQPGGS........
psToc75 ...............SIIVEIKLKELEQ.KSAEVSTEWSIV.PGRGGRPTLA..SLQPGGT........
acToc75 ...............GIIVEIKLKELEQ.KNAEVSTEWSIV.PGRNGRPTLA..SIQPGGT........
atToc75b ...............GIVFEIKLNETDQ.RVEKWGLD..........PSLD..FFEVTGN........
atToc75c ...............GVIVEVKLQETDQ.KSVEGSADRSIV.PDPGGYPSLA..SSQPSGT........
vcToc75 ...............KINVKIRCEEVQP.KSMELDLDWSFQ.L.KNGLPSLTRQSLIPGGS........
smToc75 ...............GIIVEVKLKELEQ.KTAEISTEWSIV.PGNSGRPTLA..SIQPGGN........
ppToc75a ...............GIVVEVKLKELEQ.KTAEVSTEWSIM.PGNNGRPTLS..TIQPAGS........
ppToc75b ...............GIVVEVKLKELEQ.RTAEVSTEWSII.PGSSGRPTLS..TIQPAGS........
ppToc75c ...............GIVVEVKLKELEQ.KTAEVSTEWSIV.PGNNGRPVLS..TIQPAGS........
zmToc75 ...............GIVVEIKLKELEP.KSAEVSTEWSIV.PGREGRPTLA..SIQPGGT........
bdToc75a ...............GIVVEIKLKELDP.KSAEVSTEWSIV.PGRQGRPTLA..SIQPGGT........
osToc75a ...............GIVVEIKLKELEP.KSAEVSTEWSIV.PGREGRPTLA..SIQPGGT........
osToc75b ...............GIVAIKLKEHDP.KSAQVITDWSIV.PGSQGRPTLA..SIQPGGT........
crToc75 ...............KINVKIRCEEVQP.KSMELDLDWSFQ.L.KNGIPSINRQSLIPGGS........
bdToc75b ....Q..SIQPGGS........
GsToc75 ...............SVMVILRVRERPY.SRFEPGIGYGSG........EFF.......GE........
CmToc75 AAEGQVAQAERCAEIGDVELTLRVRERPF.HRFEPGITLSRG........KLY.......GD........
mpToc75 ...............GIVVEVKLKELEQ.KTAEVSTEWSVV.PGSSGRPTLA..SIRPGGS........
pgToc75 ...............GIIVEVKLKELEQ.KTAEVNTEWSIV.PGSGGRPSLA..SIQPGGS........
ptaToc75 ...............GIIVEVKLKELEQ.KTAEVNTEWSIV.PGSGGRPSLA..SIQPGGS........
pmToc75 ...............GIIVEVKLKELEQ.KTAEVNTEWSIV.PGSGGRPSLA..SIQPGGS........
atrToc75 ...............GIIVEIKLKELEQ.KTAEVSTEWSIV.PGRQGRPTLA..SIQPGGT........
MtToc75 ...............SIIVEIKLKELEQ.KSAEVSTEWSIV.PGRGGRPTLA..SLQPGGT........
GmToc75 ...............GIVVEIKLKELEQ.KSAEVSTEWSIV.PGRGGHPTLA..SLQPGGT........
ptToc75 ...............GIIVEIKLKELEP.KSAEVSTEWSIV.PGRGGRPTLA..SFQPGGT........
slToc75 ...............GIIVEIKLKELEQ.KSAEVSTEWSIV.PGRGGRPTLA..SIQPGGT........
hvToc75 ...............GIVVEIKLKELEP.KSAEVSTEWSIV.PGRQGRPTLA..SIQPGGT........
sbToc75 ...............GIVVEIKLKELEP.KSAEVSTEWSIV.PGREGRPTLA..SIQPGGT........
NmiToc75 ...............SVVVDIVVKERQM.KTAEVETEWSIA.PGETGRPALA..SIQPGGS........
mpuToc75 ...............KVQVDIMLRERPV.KTAESELEWSIA.PGEGGKPDLV..SIKPGGS........
csToc75 ...............KVDVDIMVKERPM.KSADLEAEWQIA.PGNNGKPMLA..SIVPGGS........
olToc75 ...............KIEVDILLRERPT.KTAETELEWSIA.PGENGRPDLV..SARPGGS........
EcBamA ...............QVDVVYKVKERNT.GSFNFGIGYGTE......SGVS...................F
EcTamA ...............KVLPLTGVVSPRTENTIETGVGYSTD...............VGP......RV
NmOMP85 ...............KVDLNMSLTERST.GSLDLSAGWVQD.......TGLV...................M
BpFhaC .YG............YSYLDLQLQRRAL.PRVSLGMDNSGP.................GTPENGRYKY
ScSam50 ....GT........
NcTob55 ............DRTELDVSIRVKEQSR.LVFKAGTDFGNA.......................EGS.......A

```
                                         430       440                 450         460
AtOEP80a                          .FAYSHR..NLFGRNQKLNVSLE...............RG.Q.IDSIFRINY.TDP......WIEGD..
AlOEP80a                          .FAYSHR..NLFGRNQKLNVSLE...............RG.Q.IDSIFRINY.TDP......WIEGD..
GmOEP80a                          .FAYSHR..NVFGKNQKLNISLE...............RG.Q.IDSVYRINY.TDP......WIQGD..
MtOEP80a                          .FAYSHR..NVFGRNQKLNVSLE...............RG.Q.VDLIVRANY.TDP......WIQGD..
PsOEP80                           .FAYSHR..NVFGRNQKLNISLE...............RG.Q.VDLIVRANY.TDP......WIQGD..
SlOEP80a                          .CAIYHK..NLFGRNQKLNLSLE...............RG.Q.IDSIFRINY.TDP......WIEGD..
HvOEP80a                          .FAYSQR..NVFGRNKKLNLSLE...............RG.Q.IDSIFRLNY.TDP......WIDGD..
SbOEP80a                          .FAYSHR..NVFGRNKKLNLSLE...............RG.Q.IDSIFRLNF.TDP......WIDGD..
PtOEP80a                          .FAYSHR..NVFGRNQKLNISLE...............RG.Q.IDSIFRINY.TDP......WIEGD..
AcOEP80a                          .FAYSHR..NVFGRNQKLNVSWE...............RG.Q.IDSIFRINY.TDP......WIEGD..
BdOEP80a                          .FAYSHR..NVFGRNKKLNLSLE...............RG.Q.IDSIFRLNY.TDP......WIDGD..
OsOEP80a                          .FAYSHR..NVFGRNKKLNLSLE...............RG.Q.IDSIFRLNY.TDP......WIDGD..
ZmOEP80a                          .FAYSHR..NVFGRNKKLNLSLE...............RG.Q.IDSIFRLNF.TDP......WIDGD..
PpOEP80                           .FAYSHR..NAFGRNQKLNVSLE...............RG.Q.VDSMFRINY.TDP......WIEGD..
SmOEP80a                          .FAYSHR..NVFGRNQKLNVSLE...............RG.Q.VDSMFRINY.TDP......WIEGD..
SmOEP80b                          .FAYSHR..NVFGRNQKLNVSLE...............RG.Q.VDSMFRINY.TDP......WIEGD..
CrOEP80                           .FSYNER..NLFGLNQRLSVSAE...............IG.Q.VDKLFRLQH.TDP......WVNSD..
VcOEP80                           .FSYNER..NLFGLNQRLSVSAE...............IG.Q.VDKLFRITH.NDP......WVNSN..
MpOEP80                           .FAYSHR..NAFGRNQKLNISLE...............RG.Q.VDSMFRVNY.TDP......WIEGD..
PgOEP80                           .FAYSHR..NVFGCNQKLNISVE...............RG.Q.HDSSFKLNY.TDP......WIEGD..
PtaOEP80                          .FAYSHR..NVFGRNQKLNISLE...............RG.Q.LDSSFKINY.TDP......WIEGD..
PmOEP80                           .FAYSHR..NVFGQNQKLNISLE...............RG.Q.HDSSFKINY.TDP......WIEGD..
AtOEP80b                          .LCIKHP..NLFGGSEKLDVSWD...............KG.L.YDSNVLVAF.RRPR.P..EWR.....
AlOEP80b                          FLCIKHP..NLFGGSEKLDVSWD...............KG.L.YDSNVLVAF.RRPR.P..EWR.....
AtOEP80c                          ......................................HG.I.KGCMIPMYW..........WL.....
GmOEP80b                          .LCIKHP..NLFGGSEKLDVSWD...............KG.L.YDSNVLVAY.RRPR.P..EWL.....
PtOEP80b                          .LCIKHP..NLFGGSEKLDVSWD...............KG.L.SDSNVSITY.RRPR.P..EWL.....
MtOEP80b                          .LCIKHP..NLFGGSEKLDVSWD...............KG.L.YDSNILIAY.RRPR.E..ECF.....
psOEP80tr                         .LCIKHP..NLFGGSEKLDVSWD...............KG.L.YDSNILIAY.RRPR.Q..ECR.....
SlOEP80b                          .LCIKHP..NLFGKSEKLDVLWD...............KG.L.YDSNILITY.RKPR.P..EWL.....
BdOEP80b                          .ISLKHP..SLFGRSEKLDVILD...............KG.I.NDSNVVVAF.RRPR.P..EWL.....
SbOEP80b                          .ISLKHP..NLFGRSEKLDVILD...............KG.I.NDSNVVVAY.RRPR.P..EWL.....
ZmOEP80b                          .ISLKHP..NLFGRSEKLDVILD...............KG.I.NDSNFVIAY.RRPR.A..EWL.....
OsOEP80b                          .ISLKHP..SLFGRSEKLDVILD...............KG.I.NESNAVIAF.RRPR.P..EWL.....
HvOEP80b                          .ISLKHP..SLFGRSEKLDVVLD...............KG.V.NDSNVVVAF.RRPR.P..EWL.....
AcOEP80b                          .LCIKHP..NLFGGSEKLDVSWD...............KG.L.YDSNVAIAY.RRPR.P..EWL.....
NmiOEP80                          .FAYSHR..NAFGRNQKLNISLE...............RG.Q.FDSMFKINY.TDP......WLEGD..
CsOEP80                           .ASFSQR..NLFGLNQKLAATVE...............IG.Q.VDSLFRVNH.TDP......WVKGD..
MpuOEP80                          .ADFFRR..NLFGRCQTLTARLEVSP......SVTGGGRA.E.VD..LKVTH.TDP......WV.GD..
OlOEP80                           .FSAFHT..NLFGQGKMLNLGVEATPRKGGRGLTVV..RP.Q.VN....LQY.QDP......WVGFG..
AtrOEP80a                         .FSYSHR..NVFGRNQKLNISLE...............KG.Q.SGSIFRINY.TDP......WIQGD..
AtrOEP80b                         .LDIKHP..DLFGRRDKLDVLWD..............HNG.L.SHSNLLISYLSKPT.S..QWLA....
Gloeobacter                       .LSLQES..NLGGNNQKIGLDVQ...............GG.G.QILLFNLSF.TDP......WIAGD..
Synechocystis6803                 .ISYQER..NLGGNNQTIGVEAQ...............VG.Q.RELLFDVSF.TDP......WIGGD..
Cyanothece8802                    .VSYQEQ..NLGGNAQTIGGEVQ...............VG.E.RELLFDINF.TDP......WIAGD..
Cyanothece51142                   .ISYQEQ..NLGGNAQTLGGEVQ...............VG.E.RELLLDVSF.TDP......WIAGD..
Oscillatoria                      .ISYQEQ..NLGGNNQRLGAELQ...............IG.E.RATLFDVSF.TDP......WIAGD..
Microcoleus                       .LSYQQQ..NLRGRNQKLGAEVQ...............IG.E.RQTLFDLSF.TDP......WIAGD..
Microcystis                       .VSYQQR..NFGGNNQTLGAEVQ...............LG.E.REFLFDLSF.SDP......WIAKD..
Nostoc73102C                      .VSYQQQ..NLNGRNQKLGAEVQ...............VG.E.RELLFDVRY.TDP......WIAGD..
Anabaena_variabilis               .VSYQQQ..NLNGRNQKLGAEVQ...............LG.E.RELLFDLRF.TDP......WIGGD..
Trichodesmium101                  .VSYQQQ..NIGGNNQKLGGEFQ...............VG.E.RLVLADVSF.TDP......WIGGD..
Thermosynechococcus               .VAFQQN..NLFGRNWKLSAEAQ...............GGTE.GEFLFDLSF.TDP......WIKGD..
Synechococcus8102                 .VQLQDS..NFFGRAWNIGLNVT...............YG.Q.YGGLANLNF.TDP......WIKGD..
Synechococcus8109                 .VQLQET..NLLGRAWNLGTNIT...............YG.Q.YGGLANLNF.SDP......WIYGD..
Nostoc7107                        .VSYQQQ..NLNGRNQKLGAEVQ...............VG.T.RELLFDLRY.TDP......WIAGD..
Gloeocapsa                        .LSYQEQ..NLGGNNQKVGGELQ...............VG.Q.REVLFDLRF.TDP......WIAGD..
Nostoc7120a                       .VSYKDA..NISGLNDQLATTVQ...............VS.T.KDVQFNSRF.TSP......YRLGQ..
Nostoc7120b                       .LNYQDQ..NIGGKNDTLLANVG...............LS.R.TDLQFDTKF.ISP......YRDTN..
Nostoc7120c                       .VSYQQQ..NLNGRNQKLGAEVQ...............LG.E.RELLFDLRF.TDP......WIGGD..
Prochlorococcus_marinus_1375      .AGLQEK..NLLGRSWSSDLNFT...............YG.E.YGALVTFSL.ADP......WIKGN..
Prochlorococcus_marinus_1986      .MGLSET..NALGRAWSTNINLN...............FG.E.YSTTYNFSL.SDP......WIKGD..
Prochlorococcus_marinus_9313      .IQLQDS..NLLGRAWNMALNIT...............YG.Q.YGGLGSISF.TDP......WIKGD..
Nostoc73102d                      .VSYQQQ..NLNGRNQKLGTEIQ...............VG.E.RELLFDLRY.TDP......WIAGD..
Nostoc73102b                      .ISYQDQ..NVGGVNDTLGVNVG...............VS.S.RDLQFNSKF.ISP......YRATN..
Nostoc73102a                      .VGYKDE..NVSGLNDKLDTILQ...............FS.T.KDVQFDGQF.TSR......YRSEE..
Synechococcus_sp._JA-3-3Ab        .LNVSEE..NLGGNNQSASFNVQ...............VG.T.NETLFDLNF.TDP......RIATL..
Synechococcus_sp._JA-2-3B’a       .LNVSEQ..NLGGNNQSASFNVQ...............VG.T.NETLFDLNF.TDP......RIATL..
alToc75c                          .VSFEHR..NIKGLNRSLIGSVTTSNF...........LNPEQDDLSFKLEY.VHP......YLDGVNN
alToc75a                          .VTFEHR..NLQGLNRSLMGSVTTSNF...........LNPQ.DDLSFKLEY.VHP......YLDGVYN
alToc75b                          .CTLGRQ..NFEGLNCSLMSAVTTSNI...........FDPKLDDLLSKLEY.VR.......CLDGDKN
atToc75a                          .VTFEHR..NLQGLNRSLMGSVTTSNF...........LNPQ.DDLSFKLEY.VHP......YLDGVYN
psToc75                           .ITFEHR..NLQGLNRSLTGSVTTSNF...........LNPQ.DDLAFKMEY.AHP......YLDGVDN
acToc75                           .VTIEHR..NLQGLNRSLVGSITTSNF...........IDPQ.DDLSFKFEY.VHP......YLDGVYD
atToc75b                          .CNLGRP..NSEGSNQSLMGSVTIRNI...........FNPKLDDLLSKIEY.VR.......FLEAVKK
atToc75c                          .ISFEHP..NIKGLNRSLIGSIATSNF...........LNPE.DDLSFKLEY.VHP......YLDGVSN
vcToc75                           .VEVTRE..NLFGNSESATLSLSASDW...........RNPS.ADLGFTFSY.SEP......FY.....
smToc75                           .VTFEHR..NIKGLNRTLICTVTSNNL...........FNPQ.DDLGFKMEY.VHP......YLDGVTD
ppToc75a                          .VTFEHR..NLQGLNRSILGVVTSNNL...........FNPQ.DDLGFKAEY.VHP......HVDGVDD
ppToc75b                          .VTFEHR..NIQGLNRSILGVVTSNNL...........FNPQ.DDLGFKAEY.VHP......YVDGIDD
ppToc75c                          .VTFEHR..NLQGLNRSILGVVTSNNL...........FNPQ.DDLGFKAEY.VHP......CVDGVNN
zmToc75                           .VSFEHR..NIYGLNRSIVGSVTSSNL...........LNPQ.DDLSFKLEY.VHP......YLDGVDD
bdToc75a                          .VSFEHR..NIYGLNRSIVGSVTSSNL...........LNPQ.DDLSFKLEY.VHP......YLDGVED
osToc75a                          .VSFEHR..NIYGLNRSIVGSVTSSNL...........LNPQ.DDLSFKLEY.VHP......YLDGVDD
osToc75b                          .VSFEHR..NICGLKRSLIGSVTSSNL...........LNPE.DDLSFKLEY.AHP......YLDGIDN
crToc75                           .VEVSHE..NLFGNSESATLSLSASDW...........RNPS.ADLGFSVAY.SEP......FY.....
bdToc75b                          .VSFEHR..NICGLNRSLTGSVASSNL...........LNPQ.DDLSFKLEY.THP......YLDGVKN
GsToc75                           .LSFQDN..NLFGRNQFLQFELQ......KR..........QSQHSLLSLEW.EDPRVG..QWF.....
CmToc75                           .LTLEDA..NVAGRGIRFQADIR...............SKPDRSDDAAHIVL.QNPRLG..........
mpToc75                           .VTFEHR..NLYGLNRSILGCVTSSNL...........FNPQ.DDLGFKAEY.VHP......YVDGVDD
pgToc75                           .VTFEHR..NLKGLNRSILGAVTSNNL...........FNPQ.DDLGFKMEY.VHP......YLDGVRD
ptaToc75                          .VTFEHR..NLKGLNRSILGAVTSNNL...........FNPQ.DDLGFKMEY.VHP......YLDGVRD
pmToc75                           .VTFEHR..NLKGLNRSILGAVTSNNL...........FNPQ.DDLGFKMEY.VHP......YVDGVRD
atrToc75                          .VSFEHR..NIKGLNRSLLGSVTSNNL...........LNPQ.DDLSFKFEY.VHP......YVDGVYN
MtToc75                           .ITFEHR..NLQGLNRSLTGSVTTSNF...........LNPQ.DDLAFKMEY.AHP......YLDGVND
GmToc75                           .VSFEHR..NLQGLNRSINGSITTSNF...........LNPQ.DDLAFKLEY.VHP......YLDGVYY
ptToc75                           .VSFEHR..NIKGLNRSILGSITTSNF...........FSAQ.DDLSFKLEY.VHP......YLDGVYN
slToc75                           .VSFEHR..NLYGLNRSILGSVTTSNF...........LNPQ.DDLAFKLEY.VHP......YLDGVYN
hvToc75                           .VSFEHR..NIYGLNRSIVGSVTSSNL...........LNPQ.DDLSFKLEY.VHP......YLDGVDD
sbToc75                           .VSFEHR..NIYGLNRSIVGSVTSSNL...........LNPQ.DDLSFKLEY.VHP......YLDGVDD
NmiToc75                          .VFFEHR..NIAGENRSIYGSVSTSNL...........FNPQ.DDLGFKIEY.LHP......YVYGDDD
mpuToc75                          .VFFEHR..NLDGWGRQLYGSVSTANF...........LQPQ.DDLGFKLEY.HHP......YVLGDGD
csToc75                           .LTFEHR..NIARRGRQATASITAQNF...........LAPA.DDLGYKVEY.RHP......YFFNSKD
olToc75                           .VFFEHR..NLDSWGRQLFGSISTANF...........LEPQ.EDLGFKLEY.NHP......YCKGDAD
EcBamA                            QAGVQQD..NWLGTGYAVGINGT...............KN.D.YQTYAELSV.TNP......YFTVDGV
EcTamA                            KATWKKPWMNSYGHSLTTSTSIS...............APEQTLDFSYKMPLLKNPL...EQYY.....
NmOMP85                           SAGVSQD..NLFGTGKSAALRAS...............RS.K.TTLNGSLSF.TDP......YFTAD..
BpFhaC                            NASVTAN..DLLGLNDTLGLYIGNRYYRD.........AGHD.AERNYDLMY.SVPL........GRTR
ScSam50                           .........N.FGNDNDAEAYLQ....................FEKLIDKKYLKLPTRVNLEILRGT..
NcTob55                           YTNAVLR..NIFGGAETLSVNAA...............AGTR.TRSAYNAVF.STP.......VNGN..
```

```
                                              470        480                 490
                                                 .          .                   .
AtOEP80a                          .DKRTSRSI..MVQNSRTPGNLVH.GNQ............PDNSS.........................
AlOEP80a                          .DKRTSRSI..MVQNSRTPGNLVH.GNQ............PDNSS.........................
GmOEP80a                          .DKRTSRTI..MIQNSRTPGTIVH.GNA............DGNGS.........................
MtOEP80a                          .DKRTSGTI..MVQNSRTPGTIVH.GNL............DGNSS.........................
PsOEP80                           .DKRTSRTI..MIQNSRTPGTIVH.GNQ............DGNSN.........................
SlOEP80a                          .DKRTSRSI..MIQNSRTPGTLVH..NH............PG.GS.........................
HvOEP80a                          .NKRTSRTV..MVQNSRTPGTLIHGGEH............PDHSP.........................
SbOEP80a                          .NKRTSRTV..MVQNSRTPGTLVHGGDH............PDHGP.........................
PtOEP80a                          .DKRTSRTI..MVQNSRTPGNLVH.GNQ............PVNNS.........................
AcOEP80a                          .DKRTSRSI..MVQNSRTPGTVVH.GNQ............PDNSN.........................
BdOEP80a                          .NKRTSRTV..MVQNSRTPGTLIHGGDH............PDHGA.........................
OsOEP80a                          .NKRTSRTI..MVQNSRTPGTLIHGGDH............PDHGP.........................
ZmOEP80a                          .NKRTSRTV..MVQNSRTPGTLVHGGDH............PDHGP.........................
PpOEP80                           .DKRTSRSI..NIQNSRAPGTTVH.GVRRDALTSG.....PLHGG.........................
SmOEP80a                          .DKRTSRAI..NIQNSRSPASMTH.GVRRDVSSSG.....PSHGG.........................
SmOEP80b                          .DKRTSRAI..NIQNSRSPASMTH.GVRRDVSSSG.....PSHGG.........................
CrOEP80                           .PHRTSRTV..QVMNNRTSGNSIH.GKA............ESDGDDG.......................
VcOEP80                           .PHRTSRTI..HLMNNRTSGNTIH.GRA............EADGDDG..GSGAAV...............
MpOEP80                           .DKRTSRSI..NIQNSRSPGTIVH.GIRRDAPSSV.....PVHGG.........................
PgOEP80                           .DKRTSRSM..IVQNSRSPGTLVH.GTS............PGSNNSP..DSRTP................
PtaOEP80                          .DKRTSRSI..TVQNSRSSGTLVH.GMS............PGSNNSP..DSRTP................
PmOEP80                           .DKRTSRSI..IVQNSRTPGTLVH.GMS............PGSNNSS..DSRTQ................
AtOEP80b                          ....PQQCF..FIQHSLSPEIGVH.GTPVDN.......................................
AlOEP80b                          ....PQQCF..FIQHSLSPEIGVH.GTPVDN.......................................
AtOEP80c                          .................LGDLDLN.G..VRN.......................................
GmOEP80b                          ....AQQSF..VLQHSLSPEIGVH.GIPVNN.......................................
PtOEP80b                          ....CQKCF..VIQHSFSPEIGVH.GTPIDN.......................................
MtOEP80b                          ....AHQSF..VVQHSLSPEIGIH.GIPVNN.......................................
psOEP80tr                         ....AHQSF..TVQHSFSPEIRTH.GIPMNN.......................................
SlOEP80b                          ....AQQSF..VVQHSISPEIGVH.GLPDDN.......................................
BdOEP80b                          ....SQQSF..VIQHSMTPEIAVH.GFPADN.......................................
SbOEP80b                          ....SQQSF..VIQHSMTPEVAVH.GFPADN.......................................
ZmOEP80b                          ....SQQSF..VFQHSMTPEVAVH.GFPADN.......................................
OsOEP80b                          ....SQQSF..VIQHTMTPEIAVH.GFPADN.......................................
HvOEP80b                          ....SQQSF..VIQHSMTPEVAVH.GFPADN.......................................
AcOEP80b                          ....SQQSF..VIQHSVSPEIGVH.GVPVDN.......................................
NmiOEP80                          .DKRTSRAI..NIQNSRSPGASIH.GVRREAQGSSD..RTPWHGG.........................
CsOEP80                           .PYRTSRTI..SLQNTRTSGNPVH.GRA............PDDCARP..GSEEE................
MpuOEP80                          .ANRTSRRT..YLDTETNTLEHIH.GKT...........EPALGDDD..GDGAG................
OlOEP80                           .PTRTSRAM..SVRSESSSMRSTH.GVN..........STNVDGDQP..D....................
AtrOEP80a                         .DKRTSRSI..WVQNSRTPGMLVH.GDH............SRHGG.........................
AtrOEP80b                         ....AQQSF..FLQHSVAPEITIH.GVPIDN.......................................
Gloeobacter                       .PNRTSLST..NIFNRQAFSYVFN.GGGVGVLNPRTGFVETP............................
Synechocystis6803                 .PFRTSYTA..NLFRRRTISLVFD.GADSS........IRTFNGF.........................
Cyanothece8802                    .PFRTSYTV..NGFRRRTISLVFD.GDDSS........IRTLNEF.........................
Cyanothece51142                   .PYRTAYTV..NGFRRRTISLVFD.GDDSS........IRTLNGF.........................
Oscillatoria                      .PYRTSYTV..NAFRRRSISVIFD.GGDNE........VRLENG..........................
Microcoleus                       .PYRTSYTI..NAFRRRSISVIFD.GGERE........VRLPND..........................
Microcystis                       .NFRTSYTV..NVFRRQSISLVYD.GENSS........IRTLNDN.........................
Nostoc73102C                      .PYRTSYTT..NLFRRSSISLIFD.GKDED........IRTFDPNNP..S....................
Anabaena_variabilis               .PYRTSYTA..NIFRRRSISLIFD.GKDED........IRTFDPGNP..N....................
Trichodesmium101                  .DHRLSYTV..NAFRRRTISVIFD.SDDDDQ.....RDVDLPNG..........................
Thermosynechococcus               .PYRTSYTV..SAFNRLTVPYTFS.NGPID........VRLANG..........................
Synechococcus8102                 .SHRTSFRG..SLFLSQQVPQVFQ.SEDSGN.......IRTIDGYE....DNGNKYAYDV.......GRK
Synechococcus8109                 .KHRTSFRT..SLFLSQQVPQVFQ.SEENGN.......IRTAEEYA....NNGSNFAYEI.......GNV
Nostoc7107                        .PYRTSYTA..NIFRRSSISLVYE.GTDEN........VETFR..NP..G....................
Gloeocapsa                        .PFRTSYTV..NAFRRRSISLVFD.SDDEQ........FEVIN..ND..G....................
Nostoc7120a                       .PERLGYSV..TAFRDRQISRTFD............DEIKLANG..........................
Nostoc7120b                       .SDRLGYTV..NAFRRREISETFD............DEIKLANG..........................
Nostoc7120c                       .PYRTSYTA..NIFRRRSISLIFD.GKDED........IRTFDPGNP..N....................
Prochlorococcus_marinus_1375      .KYKKSFRT..SLFLSRDVPQEFR.SSEGGN.......IAGVSDYYQVPGSSETSKVYDID.........
Prochlorococcus_marinus_1986      .KYKTSFRT..NVFLSRDYPQEFK.SENHGR.......IYAVDDTT........................
Prochlorococcus_marinus_9313      .AHRTSFRT..SIFLSREVPQVFQ.SQNNGN.......IRTLKDYY....DGGSSYAYDIDENDNPAGRK
Nostoc73102d                      .PYRTSYTA..NIFRRSSISLIFD.GKNNN........IKTFE...Q..G....................
Nostoc73102b                      .PDRLGYTV..NGFRNRELSETFD............DKIKLANG..........................
Nostoc73102a                      .PNRLGYSI..RGFRNRNTSGTFN............EDIRLANG..........................
Synechococcus_sp._JA-3-3Ab        .EIPTSYNV..NIANQQSSSFVFN.EG...........LTLPNG..........................
Synechococcus_sp._JA-2-3B'a       .DIPTSYNV..NISNQQSSSFVFN.EG...........FTLPNG..........................
alToc75c                          .PRNRTFKT..SIFNSSKLSPVFTGGPGFEE.......................................
alToc75a                          .PRNRTFKT..SCFNSRKLSPVFTGGPGVEE.......................................
alToc75b                          .PRNRTFKT..SFFNSRKLSPVFTGGPGFEE.......................................
atToc75a                          .PRNRTFKT..SCFNSRKLSPVFTGGPGVEE.......................................
psToc75                           .PRNRTLRV..SCFNSRKLSPVFTGGPGVDE.......................................
acToc75                           .PRNRTFRA..SCFNSRKLSPVFTGGPGVDE.......................................
atToc75b                          .PRNRTFKT..SFFNSRKLSPVFTGGPGYED.......................................
atToc75c                          .PRNRTFKT..SIFNSSKLSSVLTGGPGFEE.......................................
vcToc75                           .KPHTTRNL..QLFNTRKTSTIFTPG.GEAE.......................................
smToc75                           .PRERTLKA..SCFNSRKVSAVFTGGPGLED.......................................
ppToc75a                          .PRNRTFRA..SCFNSRKLSAVFTGGPGVED.......................................
ppToc75b                          .PRNRTFRA..SCFNSRKLSAVFTGGPGMED.......................................
ppToc75c                          .PRNRTFRA..SCFNSRKLSAVFTGGPGVED.......................................
zmToc75                           RSKNRTFKT..SCFNTRKLSPVFVAGPNMDE.......................................
bdToc75a                          RNKNRTFKT..SCFNTRKLSPVFVAGPNMDE.......................................
osToc75a                          RNKNRTFKT..SCFNTRKLSPVFVAGPNMDE.......................................
osToc75b                          LSRNRTFKI..SCFNSRKLSPIFVAGPNMYE.......................................
crToc75                           .KPHTTRNA..QLFNTRKTSTIFTPG.GESE.......................................
bdToc75b                          HGKNRTFKT..SCFNTKKLSPVF...............................................
GsToc75                           .....GYRF..KVY........................................................
CmToc75                           .SKAGGYQV..RLFESRSTAVGSRRGVEVTLMAPQ...................................
mpToc75                           .PRNRTFKA..SCFNSRKLSAVFTGGNGQEE.......................................
pgToc75                           .PRNRTLRA..SCFNSRKLSPVFTGGPGIEE.......................................
ptaToc75                          .PRNRTFRA..SCFNSRKLSPVFTGGPGIEE.......................................
pmToc75                           .PKNRTFRA..SCFNSRKLSPVFTGGPGLEE.......................................
atrToc75                          .PRNRTFRA..SVFNSRKLSPVFTGGPGMEE.......................................
MtToc75                           .LRNRTLRV..SCFNSRKLSPVFTGGPGVDE.......................................
GmToc75                           .SRNRTLRV..SCFNSRKLSPVFTGGPGVDE.......................................
ptToc75                           .PRNRTLRG..SCFNSRKLSPVFTGGPGVDE.......................................
slToc75                           .PRNRTLRT..SCFNSRKLSPVFTGGPGVDE.......................................
hvToc75                           RNKNRTFKT..SCFNTRKLSPVFVAGPNMDE.......................................
sbToc75                           RSKNRTFKT..SCFNTRKLSPVFVAGPNMDE.......................................
NmiToc75                          .PARTTLRI..SGFNSRKLSAVFTGGPSLEE.......................................
mpuToc75                          .PDKTGFKT..CVFNSRKLSPVFTGGPLMDE.......................................
csToc75                           .PKRTALAV..SIFNARKLSGVFTPGEILAD.......................................
olToc75                           .SDKTSFKA..AAFNSRKLSPVFTGGPLIQD.......................................
EcBamA                            .SLGGRLFY..NDFQADDADLS................................................
EcTamA                            .LVQGGFKR.TDLNDTESDSTTLV..............................................
NmOMP85                           .GVSLGYDVYGKAFDPRKASTSIK..............................................
BpFhaC                            LDLQTGYSTYRNLLKTRYGQYQSA.GNSRSF.......................................
ScSam50                           .KIHSSFLF..NSYSSLSPQSILN..............LKVFSQF.........................
NcTob55                           ......................................................................
```

```
                                                                      500
AtOEP80a                      ..................................LTIGRVTAGVEYS.RPF............RP.....
AlOEP80a                      ..................................LTIGRVTAGIEYS.RPF............RP.....
GmOEP80a                      ..................................LTIGRITGGIEFS.RPI............RP.....
MtOEP80a                      ..................................LTIGRITGGVELS.RPI............RP.....
PsOEP80                       ..................................LTIGRITGGIELG.RPI............RP.....
SlOEP80a                      ..................................LTIGRVTAGIEYS.RPF............RP.....
HvOEP80a                      ..................................ITIGRITAGIEYS.RPF............RP.....
SbOEP80a                      ..................................ITIGRVTAGLEYS.RPF............RP.....
PtOEP80a                      ..................................LTIGRVAAGIEFS.RPL............RP.....
AcOEP80a                      ..................................VTIGRVTAGIEFS.RPF............RP.....
BdOEP80a                      ..................................IIIGRVTAGIEYS.RPF............RP.....
OsOEP80a                      ..................................ITIGRVTAGIEYS.RPF............RP.....
ZmOEP80a                      ..................................ITIGRVTAGLEYS.RPF............RP.....
PpOEP80                       ..................................VTIGRLTAGVDYS.RPL............RP.....
SmOEP80a                      ..................................VTVSRLMASVDYS.RPL............RP.....
SmOEP80b                      ..................................VTVSRLVASVDYS.RPL............RP.....
CrOEP80                       ..........................GGAGG..GVLLGRLQGGVEWQ.RPL............SS.....
VcOEP80                       ...................GGGGVGAGSGGG..GVLLGRLQGGVEWQ.RPL............SE.....
MpOEP80                       ..................................VTIGRLMAGVDYS.RPL............RP.....
PgOEP80                       ..........................VSSDV..AATIGRQMASVDYS.RPL............RP.....
PtaOEP80                      ..........................VSSDG..IVTIGRQIASVDYS.RPL............RP.....
PmOEP80                       ..........................VSSDG..VVTIGRQMASVDYS.RPL............RP.....
AtOEP80b                      ..........................FSRSGSGGVNLSKLALGLDLS.EPA............SS.....
AlOEP80b                      ..........................FSRSGSGGVNLSKLAVGLDLS.EPA............SS.....
AtOEP80c                      ..........................SASSYSGGVNLSKLAVGLDLS.EPA............SS.....
GmOEP80b                      ..........................FSRSGSGGINLSRLSVGMDLK.EPA............SS.....
PtOEP80b                      ..........................FSRSGSGDVNLSRLSVGLDRN.EPA............SS.....
MtOEP80b                      ..........................FSRSGSGGVNLSRLSVGMDLK.EPA............SS.....
psOEP80tr                     ..........................FSRSGSGGVNLSRLSVGMDLK.EPV............SS.....
SlOEP80b                      ..........................FSRSGSGGVNLSRLSAGLDLS.EPA............SS.....
BdOEP80b                      ..........................FTRSGSRGVNLSRLSLGVELN.EPT............TS.....
SbOEP80b                      ..........................FTRSGSRGINLSRLSFGLELN.EPA............TS.....
ZmOEP80b                      ..........................FTRSGSRGINLSRLSFGLELN.EPA............TS.....
OsOEP80b                      ..........................FTRSGSRGINLSRLSLGVELN.EPS............TS.....
HvOEP80b                      ..........................FTRSGSRGVNLSRLSLGVEIN.EPT............TS.....
AcOEP80b                      ..........................FSRSGSGGVNLSRLSAGINLN.EPA............SS.....
NmiOEP80                      ..................................ITIGRLVAGVDYS.RPL............RP.....
CsOEP80                       ..........................TSPEG..SVVVARLMGGVEYG.RPL............AT.....
MpuOEP80                      .....................GAADLASDDGPKGVFARRNVAGVEYR.RPL............AA.....
OlOEP80                       ..........................ITSPGLSEMSLQRLQTSVEHS.RPL............LN.....
AtrOEP80a                     ..................................VTIARETAGIEYG.RPF............RP.....
AtrOEP80b                     ..........................FSHSVSGGVKLSRFLVGLQLH.EPP............SAA..S.
Gloeobacter                   ...................................RENRLGLGVIFG.RPL............NN.....
Synechocystis6803             ................................DSPRVVRTGLGLTFF.RPIA.....DDVFA.PP.....
Cyanothece8802                ................................DSPRVVRTGGSVTFS.RPIA.....DDVFT.IP.....
Cyanothece51142               ................................DSPRVIRTGGGISFA.RPLA.....EDPFT.KP.....
Oscillatoria                  ................................DRPRILRTGGGLSFT.RPLE.....RNPFA.DS.....
Microcoleus                   ................................DRPRVVRTGAGINFT.RPLS.....RNPFA.DA.....
Microcystis                   ................................DSPRIVRTGGGITFV.RPLA.....PDVFT.KP.....
Nostoc73102C                  ...........................NTDAQDRPRILRLGGGVTFT.RPLS.....SNPYK.TS.....
Anabaena_variabilis           ...........................DTNEQDRPRVTRLGGGVTFT.RPLS.....ANPFE.RA.....
Trichodesmium101              ................................DNPRVIRTGGGVSFT.RPFI.....PNPFV.DP.....
Thermosynechococcus           ................................DFPRINRLGTALFFT.RPFT.....KDRDQVRT.....
Synechococcus8102             YKFSD.YEKVPGSVNKAEKEYPNRSWFDYEG.DSFALRKTGGSIAFT.RPLNG....GDPFK.DT.....
Synechococcus8109             YNFTA.GVAAPDDVNLAQAAYPNRSWFDYEG.DTVVLRKTGGSFSFS.RPLNG....GDPYK.DA.....
Nostoc7107                    ...........................DTDG.DRPRVLRLGGGVTFT.RPLS.....NNPYE.RS.....
Gloeocapsa                    ...........................ELLG.DRPRVLRLGGGVTFT.RPLS.....QNPLA.RS.....
Nostoc7120a                   ................................NRTREGRFGGSVAVL.RSFD..................
Nostoc7120b                   ................................DKVREGKVGTGISLQ.RPID..................
Nostoc7120c                   ...........................DTNGQDRPRVTRLGGGVTFT.RPLS.....ANPFE.RA.....
Prochlorococcus_marinus_1375  YAHSGINAAAFSSVPAAKSSDSNTSWFDYEG.DSVLLKRSGAKFSFS.RPLNG....GDPFK.KS.....
Prochlorococcus_marinus_1986  ...................TESNDTF......SSIVLEKTGGGFSFS.RPLNG....GDPFK.ET.....
Prochlorococcus_marinus_9313  F..........DSVSDAENADSNASWFDYEG.NSVALQRIGGNIIFA.RPLNG....GDPYK.KA.....
Nostoc73102d                  ...........................KTDG.DRPRVLRLGGGVTFT.RPLS.....NNSYK.NS.....
Nostoc73102b                  ................................DKVREGQIGGSVSLQ.RPID..................
Nostoc73102a                  ................................DKVREGRFGGSVALL.RAFD..................
Synechococcus_sp._JA-3-3Ab    ................................DPVRINRLGGGVTFS.RPI............GG.....
Synechococcus_sp._JA-2-3B'a   ................................DPVRINRLGGGVTFS.RPI............GG.....
alToc75c                      ..............................AVAPILVDRAGVKATIT.EDFT..................
alToc75a                      ...............................VPPIWVDRAGVKANIT.ENFT..................
alToc75b                      ..............................VVPPMFVARDCLKATIT.ENLT..................
atToc75a                      ...............................VPPIWVDRAGVKANIT.ENFT..................
psToc75                       ...............................VPSIWVDRAGVKANIT.ENFS..................
acToc75                       ...............................VPPIWVDRAGVKANVS.ENLT..................
atToc75b                      ..............................LVPPMFVGRDCLKATIT.ENLT..................
atToc75c                      ..............................AVAPILMDRA..............................
vcToc75                       ...............................VPPVFVDRFGLKGWTS.EITG..................
smToc75                       ...............................VPGIWVDRAGFKAGIT.ENFT..................
ppToc75a                      ...............................VPSVWVDRAGFKANLT.ECFT..................
ppToc75b                      ...............................VPSVWVDRAGFKANLT.ECFT..................
ppToc75c                      ...............................VPSVWVDRAGFKANLT.ECFT..................
zmToc75                       ...............................APPVWIDRVGFKANIT.ESFT..................
bdToc75a                      ...............................APPVWIDRVGFKANIT.ESFT..................
osToc75a                      ...............................APPVWVDRVGFKANIT.ESFT..................
osToc75b                      ...............................APPIWVDRIGFKANIT.ESFT..................
crToc75                       ...............................VPPVFVDRFGLKGWTS.QITG..................
bdToc75b                      .................................................NLT..................
GsToc75                       ................................................RDLDE.................
CmToc75                       ..LW...........................SAPTDEARRSSRTWL..RPAAW..........RSVMERT
mpToc75                       ...............................VPAIWVDRAGFKVNLT.ENFT..................
pgToc75                       ...............................VPAVWVDRAGFKANIT.ENLT..................
ptaToc75                      ...............................VPAVWVDRAGFKANIT.ENLT..................
pmToc75                       ...............................VPAVWVDRAGFKANIT.ENLT..................
atrToc75                      ...............................VPPIWVDRSGFKANIM.ENFT..................
MtToc75                       ...............................VPSIWVDRAGVKTNIT.ENFS..................
GmToc75                       ...............................VPPIWVDRTGVKANIT.ENFT..................
ptToc75                       ...............................VPPIWVDRAGMKANIT.ENFT..................
slToc75                       ...............................VPPIWVDRAGLKANIT.ENFT..................
hvToc75                       ...............................APPVWVDRVGFKANIT.ESFT..................
sbToc75                       ...............................APPVWIDRVGFKANIT.ESFT..................
NmiToc75                      ...............................VPAVWVDRTGAKLTVT.EHYT..................
mpuToc75                      ...............................VPAVWVDRAGAKATVT.QNFT..................
csToc75                       ...............................VPPVFIDRAGAKVGLI.EQHS..................
olToc75                       ...............................VPGIWVDRAGIKATVC.QNFT..................
EcBamA                        .................................DYTNKSYGTDVTLG.FPIN.................E
EcTamA                        ...............................................................AS.....
NmOMP85                       .................................QYKTTTAGAGIRMS.VPVT..................
BpFhaC                        ..........................................GLKAT.RLLY...........RD....T
ScSam50                       ..........................YNWNTNKGLDIGQRGARLSLRYEPLFLHKLLHNPHSNESP..TL
NcTob55                       ................................PDIRLALEALRSSTH.KPWAS....HDEHL.TGG..NL
```

```
                                   510          520                    530
AtOEP80a                           ..KWNGTAG....LIFQ...H.........AG....ARDEQGN.......PIIKDFYSS..........
AlOEP80a                           ..KWSGTAG....LIFQ...H.........AG....ARDEQGN.......PIIKDFYSS..........
GmOEP80a                           ..KWSGTAG....LVFQ...H.........AG....VRDEKGI.......PIIKDCYSS..........
MtOEP80a                           ..KWSGTAG....LIFQ...R.........AG....VCDNNGV.......PIIRDRYNS..........
PsOEP80                            ..KWSGTAG....LIFQ...R.........AG....GLDNNGV.......PIIRDRFNS..........
SlOEP80a                           ..KWNGTAG....IIFQ...R.........AG....ARDDKGN.......PIIRDYYSS..........
HvOEP80a                           ..KWSGTLG....LIFQ...H.........AG....ARDDKGN.......PMIRDFYNS..........
SbOEP80a                           ..KWSGTLG....LIFQ...H.........AG....ARDDKGS.......PVIRDFYNS..........
PtOEP80a                           ..KWSGTVG....LIFQ...H.........AG....ARNEKGD.......PKIKDHYNS..........
AcOEP80a                           ..KWSGTAG....LIYQ...R.........AG....ARDERGG.......PLIKDYYNS..........
BdOEP80a                           ..KWSGTLG....LIFQ...H.........AG....ARDDKGN.......PVIRDSYNS..........
OsOEP80a                           ..KWSGTLG....LIFQ...H.........AG....ARDDKGN.......PIIRDFYNS..........
ZmOEP80a                           ..KWSGTLG....LIFQ...H.........AG....ARDDKGN.......PAIRDFYNS..........
PpOEP80                            ..KWSGTAG....INFQ...R.........AG....ARDEHGS.......VKVVDMYGG..........
SmOEP80a                           ..KWSGTAG....LSFQ...R.........AG....ARDEHGQ.......AKAIDVYGG..........
SmOEP80b                           ..KWSGTAG....LSFQ...R.........AG....ARDEHGQ.......AKAIDVYGG..........
CrOEP80                            ..GWSGTLA....ASCQ...R.........TR....CMDERGR.......PITHDMYHA..........
VcOEP80                            ..CWSGSLS....TSCH...R.........TR....CMDERGQ.......PITHDMYHA..........
MpOEP80                            ..KWSGTAG....INFQ...R.........AG....ARDDHGQ.......PKVVDFYGG..........
PgOEP80                            ..KWSGTGG....ISFQ...R.........AGP..LQRDEHGQ.......IKIADMYGA..........
PtaOEP80                           ..KWSGTGG....ISFQ...R.........AGP..LQRDENGQ.......IKIADMYGA..........
PmOEP80                            ..KWSGTGG....ISFQ...R.........VGP..LQRDQNGQ.......IKIADMYGA..........
AtOEP80b                           ..KWSSTTS....IKFE...H.........VR....PINDDGR.......AITRDLDGF..........
AlOEP80b                           ..KWSSTTS....VKFE...H.........VR....PINDDGR.......AITRDLDGF..........
AtOEP80c                           ..KWSSTTS....VKFEVPGHYLLQTLYMCVR....LTMTDGRY..QRSGRISYNMQN...........
GmOEP80b                           ..KWSSTTS....IKFE...H.........VR....PLNDDGR.......AICRDYDGF..........
PtOEP80b                           ..DWSSSTS....IKFE...H.........VQ....LVNDDGR.......SITRDIDGF..........
MtOEP80b                           ..KWSSTTS....IKFE...H.........VR....PLNDDGR.......NISMDRDGF..........
psOEP80tr                          ..KWSSTSS....IKFE...H.........VR....PFNDDGR.......SISRDCDGF..........
SlOEP80b                           ..NWSSKTS....IKFE...H.........IR....PVSDEGR.......SINRDTHGF..........
BdOEP80b                           ..NWTSGTS....VKFE...H.........IR....PVNNDGR.......SIARDHDGF..........
SbOEP80b                           ..NWTSGTS....VKFE...H.........IR....PINNQGH.......SIARDHDGF..........
ZmOEP80b                           ..NWTSGTS....VKFE...N.........IR....PVNNQGH.......SIARDHDGF..........
OsOEP80b                           ..NWTSGTS....VKFE...H.........IR....PVNNEGR.......SIARDHDGF..........
HvOEP80b                           ..NWTSGTS....VKFE...H.........IR....PVNNDGR.......SISRDHDGF..........
AcOEP80b                           ..NWSSTTS....IKFE...H.........VR....PVNDDGR.......SISRDVDGF..........
NmiOEP80                           ..KWSGTAG....ISFQ...R.........AG....VRDDHGQ.......PKLVDIFGA..........
CsOEP80                            ..GWTGTLG....VNWQ...R.........AK....CLDEHGS.......CITQDAYGS..........
MpuOEP80                           ..SWTGTFA....ATYQ...R.........AS....THDESKR.......ECLHDAYGS..........
OlOEP80                            ..GWNGMFS....VDFK...R.........TG....VLDNDGN.......HSLFDAYGA..........
AtrOEP80a                          ..KWNGTAG....VIFQ...H.........AG....ARDDEGK.......PIIKDAYGS..........
AtrOEP80b                          TCNWTSKTS....IRFE...H.........VR....PINDYGR.......SITRDLHGF..........
Gloeobacter                        ..EWRASAG....ARFE...T.........VQ....LRDFLGR.......RYTTDNLGN..........
Synechocystis6803                  ..DWRLSAG....FGYQ...N.........VR....IENAAGA.......LSPFSAPLN......GFNSQ
Cyanothece8802                     ..DWRTSLG....LSYQ...R.........VQ....VENAEGD.......IAPLSAPLN......GFPSQ
Cyanothece51142                    ..DWRLSAG....INYQ...R.........VQ....IENADGD.......IAPLSAPIN......GFPEQ
Oscillatoria                       ..EWVASVG....FQYQ...R.........VS....IRDRNGN.......VRARDEDDN..........
Microcoleus                        ..EWTASVG....LQYQ...R.........VS....ITDRKGR.......RESEDELGN..........
Microcystis                        ..KWVVSLG....FNYQ...Q.........VQ....VQNANGD.......ISPLSAPLN......GFGSQ
Nostoc73102C                       ..VWTASAG....LQYQ...R.........VS....ARDADGN.......LRKTGAVFND....NGVPLD
Anabaena_variabilis                ..EWTASAG....LQYQ...R.........VS....TRDADGN.......LRKEGAVFDD....NGNRTS
Trichodesmium101                   ..DWTASLG....LKYE...R.........VE....IQDRDGE.......VEPRDELGN..........
Thermosynechococcus                ..AWTGSLG....LQYQ...R.........VT....SLDGGFT.......RFNTDALGNC.........
Synechococcus8102                  ..PWRVLAG....LSIS...E.........VR....PINFAAE.......TRVYGVSTNNFK..DGRVKN
Synechococcus8109                  ..KWNVVAG....MSFA...E.........VR....PINFAGD.......SRSYAVSSKKRSEGNGEVGK
Nostoc7107                         ..EWTASAG....LQYQ...R.........VS....TRDADGN.......LRRFGTVYDD....N.....
Gloeocapsa                         ..EWTASAG....LEYQ...R.........IS....IRDSDGD.......LRPQGQVGE.....DGEPTD
Nostoc7120a                        ..EWDAALG....LNYT...R.........IS....LRDKDYN.......VVRADGLGN..........
Nostoc7120b                        ..GWNASLG....FNYS...R.........TS....IRDRQGN.......ITPTDAQGN..........
Nostoc7120c                        ..EWTASAG....LQYQ...R.........VS....TRDADGN.......LRKDGAVFDD....NGNRTS
Prochlorococcus_marinus_1375       ..PWAVLWG....MDFQ...K.........IR....PIDYSSK.......DRPYGAVSANYT..NNSASN
Prochlorococcus_marinus_1986       ..KWRVLAG....MNFK...Q.........VK....MIDSDGN.......LKPYGDKTPTTE......G
Prochlorococcus_marinus_9313       ..PWNVLAG....LNIQ...K.........VR....PINFSGD.......SRPYGVANDDIE..DGRVPD
Nostoc73102d                       ..EWTASAG....LQYQ...R.........VS....TRDANGN.......IRKEGTVFK.....NGVPTE
Nostoc73102b                       ..NWNTSLG....LNYT...R.........TS....IRDRQGN.......ITPTDAQGN..........
Nostoc73102a                       ..EWDTSVA....LNYT...R.........IS....LRDREYN.......VVQVDRLGS..........
Synechococcus_sp._JA-3-3Ab         ..NWRASLG....TRVQ...F.........VE....ARNSNGE.......QERFDVLGN..........
Synechococcus_sp._JA-2-3B'a        ..NWRASLG....TRIQ...F.........VE....ARNSNGE.......QQRFDVLGN..........
alToc75c                           .RQSKLTYG....VVLE...E.........IT....TRDENGKISSNGQVLLPNGGINV.....NG....
alToc75a                           .RQSKFTYG....LVME...E.........IT....TRDESSHIAANGQRLLPSGGISA.....DG....
alToc75b                           .RQREFTYG....VMLE...E.........II....AQDENRKVSVNGLSFSPSGGISV.....NG....
atToc75a                           .RQSKFTYG....LVME...E.........IT....TRDESSHIAANGQRLLPSGGISA.....DG....
psToc75                            .RQSKFTYG....LVME...E.........II....TRDESNHICSNGQRVLPNGAISA.....DG....
acToc75                            .RQSKFTYG....LVME...K.........IT....TRDESSHIAQNGQRQLPSGGISA.....DG....
atToc75b                           .RQRELTYG....VMFE...E.........II....TRDENRRISENGLLLSPDGGISI.....NG....
atToc75c                           ...................................................AINV.....NG....
vcToc75                            .QDNKVEHA....LMLQ...L.........VS....ALDENGQVVAKGTK.VQRGYYAD.....NG....
smToc75                            .RQSKLTYG....FVME...E.........VT....TRDENSAICTNGARVLPSGALSM.....DG....
ppToc75a                           .RQSKFTYG....LVLE...E.........VT....TRDETSAICVHGARTLPSGALTS.....DG....
ppToc75b                           .RQSKFTYG....LVVE...E.........VT....TRDETSSICVHGPRTLPSGALSN.....SG....
ppToc75c                           .RQSKFTYG....LVLE...E.........VT....TRDETSSICVHGARTLPSGALTS.....DG....
zmToc75                            .RQSKFTYG....LVVE...E.........IT....TRDETNTICTHGSRAMPSGGLSM.....DG....
bdToc75a                           .RQSKFTYG....LVVE...E.........IT....TRDETNSICTHGSRAMPSGGLSM.....DG....
osToc75a                           .RQSKFTYG....LVVE...E.........IT....TRDETNSICTHGSRAMPSGGLSM.....DG....
osToc75b                           .KQSKFTYG....LIVE...E.........IT....TRDENNNICTHGSRQLPSGALSM.....IG....
crToc75                            .QDNKVEHA....LMLQ...L.........VS....TLDENGQVVAKGTK.VQRGYYAD.....NG....
bdToc75b                           .KQSKLTYG....LVVE...E.........IT....THDKSNDVCTHGLRTTATGALGM.....DG....
GsToc75                            .KHLSSRSG....LTFKVFSR........LVKHL..FVDLSST...........................
CmToc75                            LQLWNIQTG....VLCE...Q.........VRMFPSGMNPGQT....ASSSLPASADGA.....SARRNR
mpToc75                            .RQSKLTYG....MVVE...E.........VT....TRDETSAICTSGARTLPSGALSM.....EG....
pgToc75                            .RQSKFTYG....VVME...E.........IT....TRDETSTVCTNGARALPSGALSM.....DG....
ptaToc75                           .RQSKFTYG....VVME...E.........IT....TRDETSTVCTNGARALPSGALSM.....DG....
pmToc75                            .RQSKFTYG....MVME...E.........IT....TRDETSTVCTNGARALPSGALSM.....DG....
atrToc75                           .RQSKFTYG....LVLE...E.........IT....TRDETSSICTNGARALPSGGLSM.....DG....
MtToc75                            .RQSKFTYG....LVME...E.........II....TRDETNHICSNGQRVLPNGGISA.....DG....
GmToc75                            .RQSKFTYG....LVME...E.........IT....TRDESSHICANGQRVLPSGGISA.....DG....
ptToc75                            .RQSKFTYG....IVME...E.........IT....TRDESSHISSNGQRVLPSGGISA.....DG....
slToc75                            .RQSKFTYG....LVME...E.........IT....TRDESSHISARGQRVLPSGGISA.....DG....
hvToc75                            .RQSKFTYG....LVVE...E.........IT....TRDETNSICTHGSRAMPSGGLSM.....DG....
sbToc75                            .RQSKFTYG....LVVE...E.........IT....TRDETNSICTHGSRAMPSGGLSM.....DG....
NmiToc75                           .RQSRVTYG....VVME...E.........VT....TRDESSSICTHGSRALPSGAMTM.....DG....
mpuToc75                           .RQSKATAS....VVVE...E.........VT....TRDESGAVVGQGAKQGAGGALSV.....DG....
csToc75                            .RNSKASLS....VVME...Q.........VT....ARDDSGQICSHATRQLPTGMLAA.....DG....
olToc75                            .KQSKCSLG....MVLE...K.........VT....TRDDSGAVCASGAKQLPNGSLSL.....DG....
EcBamA                             YNSLRAGLG....YVHN...S.........LS......NMQPQVAM....WRYLYSMGE...........
EcTamA                             .RYWDLSSG..........WQRA.......IN.LRWSLDHFTQ...........................
NmOMP85                            .EYDRVNFG....LVAE...H.L......TVN....TYNKAPK.....HYADFIKKYGKT....DG....
BpFhaC                             RSQFSVYGG....LKLR...Q...NKNY..LA..GTRLDVSSK...........................
ScSam50                            FHEWFLETC..........WR.........ST....KICSQGT....................SA....
NcTob55                            RLAWSTDNGDDHALTYSGVWR...Q.....LT....GLSASAS...........................
```

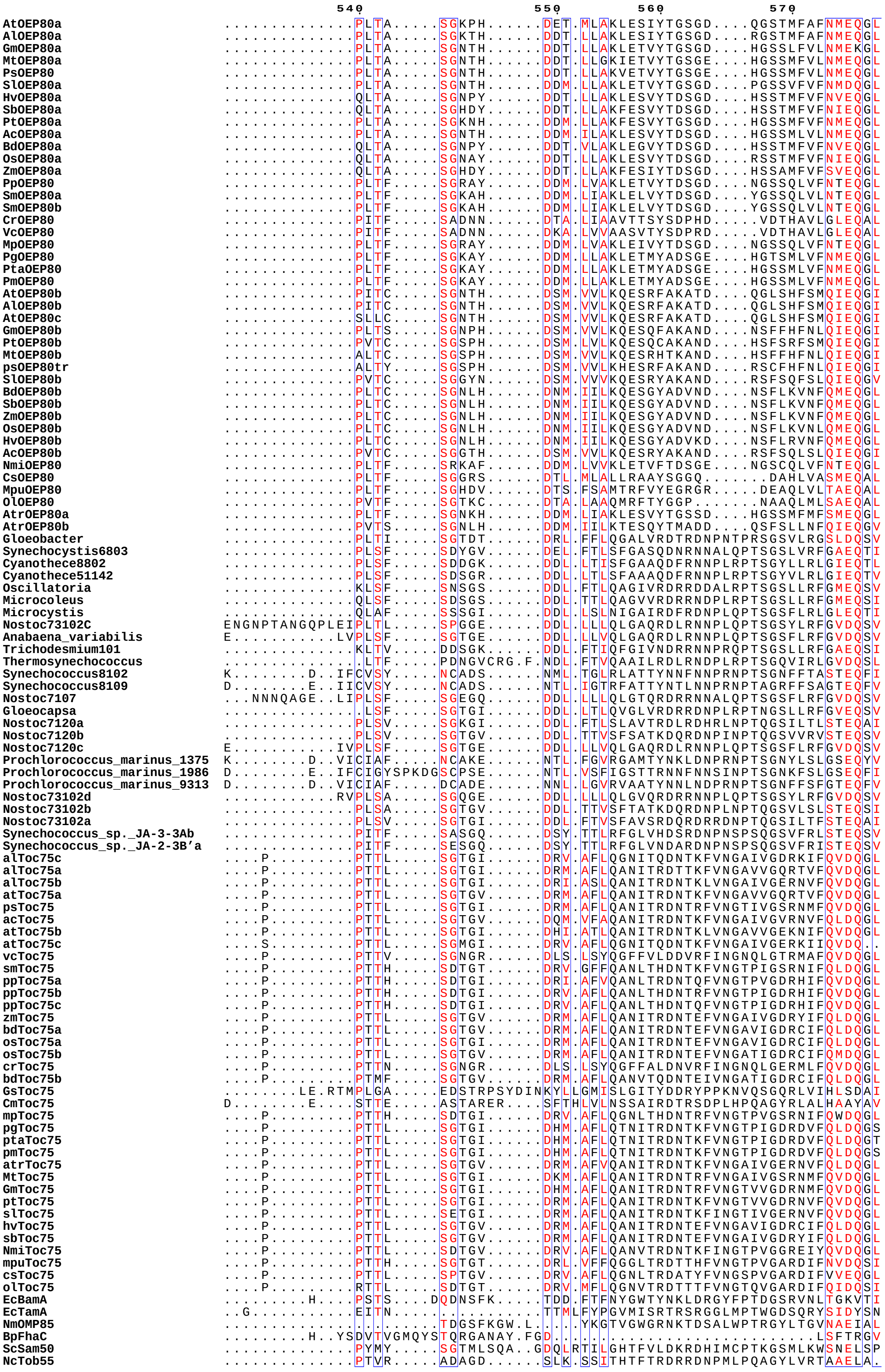

```
                            580                           590                 600
AtOEP80a                      PVLPEWL....................CFNRVTGRA.........RKGIH......I.............
AlOEP80a                      PVLPEWL....................CFNRVTGRA.........RKGIH......I.............
GmOEP80a                      PLLPEWL....................SFTRVNARA.........RKGVE......I.............
MtOEP80a                      PLLPDWL....................SFTRVNARA.........RKGVE......I.............
PsOEP80                       PVLPDWL....................SFTRVNARG.........RKGVE......I.............
SlOEP80a                      PVWSEWL....................VFNRVNARA.........RKGLV......L.............
HvOEP80a                      PILPEWL....................SFNRVTARL.........RQSYE......I.............
SbOEP80a                      PVLPEWL....................SFNRVTARL.........RQGYE......I.............
PtOEP80a                      PLWPEWL....................FFNRVNTRA.........RKGVE......I.............
AcOEP80a                      PVMPEWL....................CFNRVNGRA.........RKGME......L.............
BdOEP80a                      PILPEWL....................SFNRVTARL.........RQGYE......I.............
OsOEP80a                      PILPEWL....................SFNRVTARL.........RQGYE......I.............
ZmOEP80a                      PVLPEWL....................SFNRVTTRL.........RQGYE......I.............
PpOEP80                       PIAADWL....................FFNRINVRA.........RQGLR......L.............
SmOEP80a                      PIAPDWL....................FFNRVNIRA.........RQGVR......I.............
SmOEP80b                      PIAPDWL....................FFNRVNIRA.........RQGVR......I.............
CrOEP80                       PLRREWL....................HYTRLKLRL.........DKALR......L.............
VcOEP80                       PLRREWL....................HFTRIKARL.........DKAVR......L.............
MpOEP80                       PIAADWL....................FFNRINVRA.........RQGLR......I.............
PgOEP80                       PILPEWL....................FFNRINVRA.........RHEMR......I.............
PtaOEP80                      PVLPEWL....................FFNRLNIRA.........RHEMR......I.............
PmOEP80                       PILPEWL....................FFNRVNVRA.........RHGMR......I.............
AtOEP80b                      PVVSKWL....................IFNRFKFVA.........SKGVR......F.............
AlOEP80b                      PVVSKWL....................IFNRFKFVA.........SKGVR......F.............
AtOEP80c                      PVVSNWL....................IFNRFKFVA.........SKGVR......F.............
GmOEP80b                      PVLSKWI....................IFNRFKFVA.........SKGIK......L.............
PtOEP80b                      PILSKWL....................IFNKFKFTA.........TKGVK......L.............
MtOEP80b                      PVLSKWI....................IFNRFKFVA.........TKGIK......L.............
psOEP80tr                     PVLSKMI....................IFNRFKFAA.........SKGIK......L.............
SlOEP80b                      PILSKWL....................IFNRFKFVA.........SRGLK......L.............
BdOEP80b                      PLVPKSL....................TFNRVKCAV.........SKGIK......L.............
SbOEP80b                      PLVPKSL....................TFNRVKCAV.........SKGIK......L.............
ZmOEP80b                      PLVPKSL....................TFNRVKCAV.........SKGIK......L.............
OsOEP80b                      PLVPKSL....................TFNRVKCAV.........SKGMK......L.............
HvOEP80b                      PLVPKSL....................TFNRVKCTV.........SKGIK......L.............
AcOEP80b                      PVLSKWL....................VFNRFKFFA.........SKGVK......L.............
NmiOEP80                      PIAPDWL....................FFNRINVRA.........RQGLR......M.............
CsOEP80                       PLQSDWL....................NFNRFCLRA.........ERGMQ......I.............
MpuOEP80                      PIRPDML....................SFNRVLVRA.........QRNFH......V.............
OlOEP80                       PVQPQWL....................NYTRMVAKA.........KRSFN......LLSVGSL.......
AtrOEP80a                     PVLPEWL....................CFNRVNARV.........RKGFE......V.............
AtrOEP80b                     PLLSKWI....................AFNRFKFGA.........RKGFK......V.............
Gloeobacter                   GVLGD.V....................FFTRPTVSY.........SQFIP......MDIL.PWG......
Synechocystis6803             PVGTGNI....................MMTRLRGSY.........SYYIP......VNWLDLTGFGLVES
Cyanothece8802                PVGSGSI....................FFTRLRGSY.........SHYIP......VDFIDLG...FIDE
Cyanothece51142               PVGSGSI....................AFTRLRGNY.........SYYIP......VDFIDLG...FIEE
Oscillatoria                  PVGSGAI....................LFNRIRGSY.........SYYLP......VRYTRFA.......
Microcoleus                   PVGSGSI....................LLNRLRASY.........SFYLP......VKYTNFT.......
Microcystis                   PVGSGSI....................FGTRVRGSY.........SYFIP......VRFINSFN..LFET
Nostoc73102C                  PIGLGNI....................LLTRLRGSY.........SQYLP......IKLINLT.......
Anabaena_variabilis           PVGSGNI....................FLTRFRGSY.........SQYLP......VKFTGFS.......
Trichodesmium101              PVGSGEI....................GLNRLRGNF.........SYFIP......VSFINFT.......
Thermosynechococcus           PIGAGSI....................LMNRVRGSY.........SFYIP......VKFLRI........
Synechococcus8102             GVNEDSP....................TFNRLRGSY.........TQFFP......VNWLKLHKGCRPKA
Synechococcus8109             GINSNSP....................TFNRLRASY.........TQFFP......VDWLKLHKGCRQKP
Nostoc7107                    PVGLGNI....................LLTRLRGSY.........SQYIP......VKLLNFS.......
Gloeocapsa                    PIGSGSI....................FLNRLRGSY.........SQYLP......VNFTNFA.......
Nostoc7120a                   PLGLGNI....................FSNRFQGNY.........IQYLP......VTWIGNQNLT....
Nostoc7120b                   PIGQGNI....................SMNRLKADY.........SQYVP......VNIFNS........
Nostoc7120c                   PVGSGNI....................FLTRFRGSY.........SQYLP......VKFTGFS.......
Prochlorococcus_marinus_1375  SIGENSP....................TFNRAKASY.........SYFIP......IKWLKLHKGCRPKS
Prochlorococcus_marinus_1986  SMGNNSP....................TFNRVRATY.........AFFIP......TKLINLTKGCKSSE
Prochlorococcus_marinus_9313  SVGEHSP....................TFNRLRTSY.........THFIP......VNWLKLAKGCRPKP
Nostoc73102d                  PVGLGNI....................FLTRLRGNY.........SQYFP......VNFISLT.......
Nostoc73102b                  PIGQGNI....................SLNRLKANY.........SQYLP......VQLFNT........
Nostoc73102a                  PIGLGNI....................SSNRLRGDY.........IQYFP......VNWIGNGRPT....
Synechococcus_sp._JA-3-3Ab    RLLENGL....................NANRLETSY.........SQFIP......VKLFETRS......
Synechococcus_sp._JA-2-3B’a   RLFQNGL....................NANRLEAGY.........SQFIP......VNLFETRS......
alToc75c                      GIGSNSP....................LFNRHQLTL.........TKLIP......LRQVEEGPGK....
alToc75a                      GIGSKFP....................FFNRHQLTM.........TRFIQ......LREVEEGAGK....
alToc75b                      GIGSNFP....................LFNRHQLTL.........TRFIQ......LKQVEEGSGN....
atToc75a                      GIGSKFP....................FFNRHQLTM.........TKFIQ......LREVEQGAGK....
psToc75                       GVGSNFP....................FFNRHQLTV.........TKFLQ......LMSVEEGAGK....
acToc75                       GIGSRFP....................LFNRHQLTF.........TRFVQ......LKQVEEGAGK....
atToc75b                      GIGNNFP....................LFNRHQLSL.........TSFIQ......LKQVEEGSDK....
atToc75c                      ......................................................................
vcToc75                       NPSIRLPGG....RTIG...LSG...GIYNRATASY.........TKFMEAPFLPKLTMEQLWKER....
smToc75                       GVGSRFP....................FFNRHQLTL.........TRFIQ......LRP.EDRHGE....
ppToc75a                      GIGSKYP....................FFNRHQLTV.........TKFIQ......LRPEDKLDTE....
ppToc75b                      GIGSKYP....................FFNRHQLAV.........TRFIQ......LLSEDKVNTE....
ppToc75c                      GIGSKNP....................IFNRHQLTV.........TKFIQ......LQPEDKLDPE....
zmToc75                       GIGSKNP....................FFNRHQLTL.........TKFIN......LNKQEKGAGK....
bdToc75a                      GIGSKNP....................LFNRHQLTL.........TKFIN......LNNQEKGVGK....
osToc75a                      GIGSKNP....................FFNRHQLTL.........TKFVN......LNKQEKGAGK....
osToc75b                      GIGSKNP....................FFNRHQLTV.........TKFIN......LNKQEKGSRK....
crToc75                       NPSISLPGG....RKLG...LSG...GIYNRATASY.........TKFLEAPFLPKLTTEQLWKER....
bdToc75b                      GIGSKNP....................FFNRHQLTM.........TKFMN......LNKQENGAGK....
GsToc75                       PWNESFS....................SFIKGDIQI.........AKYLK......L.............
CmToc75                       PVTTSNP....................EYSRFGFTGSV.......YKKIP.PIRGLLRRRRRKQTEQPET
mpToc75                       GIGSKFP....................FFNRHQITF.........TRFLQ......LQPEDTKDQL....
pgToc75                       GIGSKFP....................IFNRHQLTL.........TRFIQ......LKKVEEGPEK....
ptaToc75                      GIGSKFP....................IFNRHQLTL.........TRFIQ......LKEVEEGPEN....
pmToc75                       GIGSKFP....................IFNRHQLTM.........TRFIQ......LKPVEEGPEK....
atrToc75                      GIGTNFP....................FFNRHQLSM.........TRFIQ......LKSVQEGAGK....
MtToc75                       GVGSNFP....................FFNRHQLTL.........TQFLQ......LKSVEEGAGK....
GmToc75                       GIGSQFP....................FFNRHQLTL.........TRFIQ......LMAVEEGAGK....
ptToc75                       GIGSKFP....................FFNRHQLTL.........TRFIQ......LKEVEEGAGK....
slToc75                       GVGTKFP....................FFNRHQLTM.........TQFIQ......LKQVEEGAGK....
hvToc75                       GIGSKSP....................LFNRHQLTL.........TKFIN......LNNQEKGVGK....
sbToc75                       GIGSKNP....................FFNRHQLTL.........TKFIN......LNKQEKGAGK....
NmiToc75                      GIGSRFP....................FFNRHQMTI.........TRFIQ......LREVELNSND....
mpuToc75                      GLGSGFP....................IYNRFTAGC.........TRFIQ......LGK.ESAKTS....
csToc75                       GVGSGKP....................FFNRHTVSM.........TRFLK......LRDPPVSSTS....
olToc75                       PFWPKRP....................IFNRSKVEL.........TRFVQ......LLP.ENKKNS....
EcBamA                        P...............G.......SDNEYYKVTLDT.........ATYVP......I.............
EcTamA                        TAWGSDV....................DFSVFQAQNVWIRTLYDRHRFVT...RGTLGWIETGDFD....
NmOMP85                       P...............G.......SKLQYYSATHNQ.........TWFFP......L.............
BpFhaC                        GVNNGKY...AAYDERG...PQG....NVSRFNGSLA.......WTRYMA......L.............
ScSam50                       GKHLKTQ..L.................ELNSVKSWMN........DDFIT......F.............
NcTob55                       ................GWGPLKGDVSFAKSEVELSA.........AQALP...LPGV.............
```

```
                                                           610              620
AtOEP80a                      ..........GPARFLF.............SLSGGHVVG........KFSPH.................E
AlOEP80a                      ..........GPARFLF.............SLSGGHVVG........NFSPH.................E
GmOEP80a                      ..........GPARLHL.............SISGGHVVG........NFSPY.................E
MtOEP80a                      ..........GPTRLNL.............SLSGGHVVG........NFSPY.................E
PsOEP80                       ..........GPARFNL.............SLSGGHVVG........NFSPY.................E
SlOEP80a                      ..........GPMRLLL.............SFSGGHVVG........NFPPH.................E
HvOEP80a                      ..........GPARLLL.............SASGGHVEG........NFSPH.................E
SbOEP80a                      ..........GPARLLL.............SASGGHVEG........NFPPH.................E
PtOEP80a                      ..........GPALCLL.............SLSGGHVMG........NFSPH.................E
AcOEP80a                      ..........GPARVLV.............SLSGGHVVG........SFSPH.................E
BdOEP80a                      ..........GPARLLL.............SASGGHVEG........NFSPH.................E
OsOEP80a                      ..........GPARLLL.............SASGGHVEG........NFSPH.................E
ZmOEP80a                      ..........GPARLLL.............SASGGHVEG........NFPPH.................E
PpOEP80                       ..........GPVRCIA.............SFSGGTVIG........DLAPH.................E
SmOEP80a                      ..........GQVRCITRQATFVFVFP...NFSAGNVAG........DLAPH.................E
SmOEP80b                      ..........GQVRCITRQATFVFVFPKLVSFSAGNVAG........DLAPH.................E
CrOEP80                       ..........GPYLGL............FLRGRAGAVVG........DLPPY.................E
VcOEP80                       ..........GPYLGL............YIRGRAGAVLG........DLPPY.................E
MpOEP80                       ..........GPMRCIA.............SFSGGSVIG........DLAPH.................E
PgOEP80                       ..........GLAHCIA.............SFSGGKVIG........DLAPY.................E
PtaOEP80                      ..........GLAHCIA.............SFSGGKVIG........DLAPH.................E
PmOEP80                       ..........GLVHFNA.............SVSGGKVIG........DLAPH.................E
AtOEP80b                      ..........GPAFLLA.............SLTGGSIVG........DMAPY.................Q
AlOEP80b                      ..........GPAFLLA.............SLTGGSIVG........DMAPY.................Q
AtOEP80c                      ..........GPAFPLA.............SLTGGSIVG........DMTPY.................Q
GmOEP80b                      ..........GPAFLLT.............RLTGGSIVG........DMAPY.................Q
PtOEP80b                      ..........GPAFLLA.............SLTGGSIVG........DMAPY.................Q
MtOEP80b                      ..........GPTLLLT.............RLTGGSIVG........DMAPY.................Q
psOEP80tr                     ..........GPTLLLT.............RLTGGSIVG........DMAPY.................Q
SlOEP80b                      ..........GPAFLLS.............SLTGGSIVG........DIAPY.................Q
BdOEP80b                      ..........GPTFLVT.............SLTGGSIVG........DMAPY.................Q
SbOEP80b                      ..........GPTFLVT.............SLTGGSIVG........DMAPY.................Q
ZmOEP80b                      ..........GPTFLVT.............SLTGGSIVG........DMAPY.................Q
OsOEP80b                      ..........GPTFLVT.............SLTGGSIVG........DMAPY.................Q
HvOEP80b                      ..........GPTFLVT.............SLTGGSIVG........DMAPY.................Q
AcOEP80b                      ..........GPSFFLA.............SLTGGSIVG........DMAPY.................Q
NmiOEP80                      ..........GPMRFIA.............SGSGGTVVG........DLAPH.................E
CsOEP80                       ............YKGLR..........AHLSAKGGVILG........DLPPY.................E
MpuOEP80                      ..........AGTRFFV.............AAKGGNVIG........DLPPY.................D
OlOEP80                       .........PGPVQLVL.............SSKGGSVVG........DLPPY.................E
AtrOEP80a                     ..........GPARLIM.............SLSGGHVMG........NFSPH.................E
AtrOEP80b                     ..........GPAFVFT.............GVTGGSIVG........DMAPY.................Q
Gloeobacter                   .........QGKQVLAF.............NSSVGTVFG........ALPPY.................E
Synechocystis6803             .........TQPQTVAF.............NVQAGTVLG........DLPPY.................E
Cyanothece8802                DQ.......PRPQALAF.............NVQAGTVLE........DLPPY.................E
Cyanothece51142               DQ.......PKPQALAF.............NVQAGTVLE........DLPPY.................E
Oscillatoria                  .........TGPQTLAF.............NIQAGTILG........DLPPY.................E
Microcoleus                   .........AGPQTLAF.............SFRGGTVFG........DLPPY.................E
Microcystis                   DIF......KGQQSLAF.............NVQAGTVLG........DLPPY.................D
Nostoc73102C                  .........KGAQTLAF.............NLQAGTILG........DLPPY.................E
Anabaena_variabilis           .........KGPETIAF.............NIQGGTVLG........DLPPY.................E
Trichodesmium101              .........DGPQALAF.............NIQAGHIIG........DLPPY.................E
Thermosynechococcus           .........EGPQTFAF.............NIQAGNIFG........DLPPY.................E
Synechococcus8102             GEA.....ADCPQAIGL.............QVKAGTIIG........DLPPY.................E
Synechococcus8109             GEQ.....ADCPQAIGV.............QIKGGVLMG........EAPSY.................E
Nostoc7107                    .........KGSQTLAF.............NIQGGTILG........DLPPY.................E
Gloeocapsa                    .........EGPETLAF.............NIQAGTVLG........DLPPY.................E
Nostoc7120a                   .........DNPEMVAV.............NLQLGTIFG........EFPPA.................D
Nostoc7120b                   .........QTPQVFAL.............NVQAGTVLG........NLPPY.................E
Nostoc7120c                   .........KGPETIAF.............NIQGGTVLG........DLPPY.................E
Prochlorococcus_marinus_1375  GEK.....ASCPQTLAF.............QLKAGTVVG........DLPPY.................E
Prochlorococcus_marinus_1986  VVN.....SDCPQTIGF.............EFKAGTIIG........DLPPY.................E
Prochlorococcus_marinus_9313  GEQ.....ENCPQALAF.............QVKAGTVLG........DLPPY.................E
Nostoc73102d                  .........KGLQTLAF.............NLQGGTILG........DLPPY.................E
Nostoc73102b                  .........KQPQVFAV.............NLQAGTVIG........NLPPY.................E
Nostoc73102a                  .........NNPEMLAI.............NLQIGTTIG........DFPPA.................D
Synechococcus_sp._JA-3-3Ab    .........ERPQVLAF.............DVRAGTTIG........DLAPY.................D
Synechococcus_sp._JA-2-3B’a   .........ERPQVLAF.............DVRAGTTIG........DLAPY.................D
alToc75c                      .........PQPPVLVL.............HGHYAGCVG........DLPGY.................D
alToc75a                      .........SPPPVLVL.............HGHYGGCVG........DLPSY.................D
alToc75b                      .........PRPPVLVL.............HGHYGGCIG........DLPNY.................D
atToc75a                      .........SPPPVLVL.............HGHYGGCVG........DLPSY.................D
psToc75                       .........SPPPVLVL.............HGHYGGCVG........DLPSY.................D
acToc75                       .........PPPPVLVL.............HGHYGGCVG........DLPSY.................D
atToc75b                      .........PQPPVLVL.............HGRYGGCIG........DLPSY.................D
atToc75c                      ...................................CLG........DLPGY.................D
vcToc75                       .........KAPYTVVL.............HAKAGNALG........ELGAY.................D
smToc75                       .........QPPPVLVI.............HGHYGGCIG........DLPSY.................D
ppToc75a                      .........KPPPVAVV.............HGRYGGCVG........DLASY.................D
ppToc75b                      .........RPPPVGVL.............HARYGGCLG........DLASY.................D
ppToc75c                      .........KPPLVGVI.............HGRYGGCLG........DLASY.................D
zmToc75                       .........PLPAVLVL.............HSHYAGCVG........DLPSY.................D
bdToc75a                      .........PLPAVLVL.............HGHFAGCVG........DLPSY.................D
osToc75a                      .........PLPAVLVL.............HGHYAGCVG........DLPSY.................D
osToc75b                      .........PPPAVLAL.............HGRYAGCVG........DLPSY.................D
crToc75                       .........KAPNTVVL.............HAKAGNALG........DVAAY.................D
bdToc75b                      .........PLPVVLVA.............HGRYAGCVG........DLPSY.................E
GsToc75                       ..............................PLGSTLVYGVSGKSASESLPEL.................E
CmToc75                       SHQNQEDDEEQPARACV.............ALLAEARVS......SASLPES.................E
mpToc75                       .........KPPPVLVL.............HGRYGGCLG........DLATY.................D
pgToc75                       .........PPPPVLVL.............HGRYGGCVG........DLPSY.................D
ptaToc75                      .........PPPPVLVL.............HGRYGGCVG........DLPSY.................D
pmToc75                       .........PPPPVLVL.............HGHYGGCVG........DLPSY.................D
atrToc75                      .........PPPPVLVL.............HGHYGGCVG........DLPSY.................D
MtToc75                       .........PPPPVLVL.............HGHYGGCVG........DLPSY.................D
GmToc75                       .........PPPPVLVL.............HGHYGGCVG........DLPSY.................D
ptToc75                       .........PPPPVLVL.............NGHYGGCVG........DLPSY.................D
slToc75                       .........APPPVLVL.............HGHYGGCVG........DLPSY.................D
hvToc75                       .........PLPAVLVL.............HGHYAGCVG........DLPSY.................D
sbToc75                       .........PLPAVLVL.............HGHYAGCVG........DLPSY.................D
NmiToc75                      ..........LPPIAVL.............HARYGGCVG........DLASY.................D
mpuToc75                      .........RPPPVLVA.............HARGGAVVG........NCAAY.................D
csToc75                       ..........PPPILVL.............HARAGNAIG........DMASY.................D
olToc75                       .........LPPPVVVL.............HTRAGHILG........NCAAY.................D
EcBamA                        .........DDDHKWVVLGRT.........RWGYGDGLG......GKEMPFY.................E
EcTamA                        ...............................................KVPPD.................L
NmOMP85                       .........SKTFTLML.............GGEVGIAGG...YGRTKEIPFF.................E
BpFhaC                        .....................A...GQ...PIQWASQLGF.QYSRQQLLNSY..................
ScSam50                       ............................STTIKTGYLKN...LSSQQSLPVH...............ICD
NcTob55                       ..............................SVGAGFRAG........LLYPLPMGYSLSSTSVAPSRIND
```

iv.

```
                                   630       640
AtOEP80a                           AFVIGGTNSVRGYEEGAVG.....................................SGRSYVV.......
AlOEP80a                           AFVIGGTNSIRGYEEGAVG.....................................SGRSYVV.......
GmOEP80a                           AFAIGGTNSVRGYEEGSVG.....................................SGRSYIV.......
MtOEP80a                           AFAIGGTNSVRGYEEGGVG.....................................SGRSYVV.......
PsOEP80                            AFAVGGTNSVRGYEEGGVG.....................................SGRSYVV.......
SlOEP80a                           AFVLGGTNSVRGYEEGTVG.....................................SGRSYAV.......
HvOEP80a                           AFAIGGTNSVRGYEEGAVG.....................................SGRSYAV.......
SbOEP80a                           AFAIGGTNSVRGYEEGAVG.....................................SGRSYAV.......
PtOEP80a                           AFAIGGTNSVRGYEEGAVG.....................................SGRSYAV.......
AcOEP80a                           AFAIGGTNSIRGYEEGAVG.....................................SGRSYVV.......
BdOEP80a                           AFAIGGTNSVRGYEEGAVG.....................................SGRSYAV.......
OsOEP80a                           AFAIGGTNSVRGYEEGAVG.....................................SGRSYAV.......
ZmOEP80a                           AFAIGGTNSVRGYEEGAVG.....................................SGRSYAV.......
PpOEP80                            AFPIGGTNSVRGYDEGSVG.....................................SGRSCVV.......
SmOEP80a                           AFPIGGTNSVRGYDEGAVG.....................................SGRSYAV.......
SmOEP80b                           AFPIGGTNSVRGYDEGAVG.....................................SGRSYAV.......
CrOEP80                            AFPIGGTNSVRGYAEGGVG.....................................SGRHSVE.......
VcOEP80                            AFPIGGTNSVRGYAEGCVG.....................................SGRHSIE.......
MpOEP80                            AFPIGGTNSVRGYDEGAVG.....................................SGRSCIV.......
PgOEP80                            AFPIGGTSSVRGYEEGAVG.....................................SGRSCAV.......
PtaOEP80                           AFAIGGTNSVRGYEEGAVG.....................................SGRSCAV.......
PmOEP80                            AFAIGGTNSVRGYEEGAVG.....................................SGRSCVV.......
AtOEP80b                           AFAIGGLGSVRGYGEGAVG.....................................SGRSCLV.......
AlOEP80b                           AFAIGGLGSVRGYGEGAVG.....................................SGRSCLV.......
AtOEP80c                           AFAIGGLGSVRGYGEVAVG.....................................SGRSCLV.......
GmOEP80b                           AFSIGGLGSVRGYGEGAVG.....................................SGRSCLV.......
PtOEP80b                           AFAIGGLGSVRGYGEGAVG.....................................SGRSCLV.......
MtOEP80b                           AFSIGGLGSVRGYGEGAVG.....................................SGRSCLV.......
psOEP80tr                          AFSIGGLGSVRGYGEGAVG.....................................SGRSCLV.......
SlOEP80b                           AFAIGGLGSVRGYGEGAVG.....................................CGRSCLV.......
BdOEP80b                           AFAIGGLGSVRGYGEGAVG.....................................SGRLCLI.......
SbOEP80b                           AFAIGGLGSVRGYGEGAVG.....................................SGRLCLI.......
ZmOEP80b                           AFAIGGLGSVRGYGEGAVG.....................................SGRLCLI.......
OsOEP80b                           AFAIGGLGSVRGYGEGAVG.....................................AGRLCLI.......
HvOEP80b                           AFAIGGLGSVRGYGEGAVG.....................................SGRLCLI.......
AcOEP80b                           AFAIGGYGSVRGYGEGAVG.....................................SGRSCLI.......
NmiOEP80                           AFPIGGTNSVRGYDEGAVG.....................................SGRSYMV.......
CsOEP80                            AFPIGGTNSVRGYSEGGVG.....................................SGRNYVT.......
MpuOEP80                           AFSIGGANSVRGYPEGGVG.....................................TGRKFIV.......
OlOEP80                            AFAIGGTSSVRGYNDGAIG.....................................TGRKYVV.......
AtrOEP80a                          AFAIGGTNSVRGYEEGAVG.....................................SGRSYAV.......
AtrOEP80b                          AFSIGGRSSVRGYGEGAVG.....................................TGRACLV.......
Gloeobacter                        AFTLGGGNSVRGFFEGNLG.....................................TGRSYMI.......
Synechocystis6803                  AFILGGSNSVRGYQEGELG.....................................NGRSFFQ.......
Cyanothece8802                     AFVIGGSNSVRGYPEGEVG.....................................AGRSYFQ.......
Cyanothece51142                    AFVVGGSNSVRGYAEGEVG.....................................SGRSYFQ.......
Oscillatoria                       AFALGGANSVRGYDEGDVG.....................................AGRSFIQ.......
Microcoleus                        AFALGGANSVRGYAEGDLG.....................................AGRSFLE.......
Microcystis                        AFIVGGSNSVRGYAEGEVG.....................................SGRSYFQ.......
Nostoc73102C                       AFTLGGSNSVRGYEEGALG.....................................SGRSYVQ.......
Anabaena_variabilis                AFTLGGSNSVRGYEEGALG.....................................SGRSFVQ.......
Trichodesmium101                   AFALGGTNTVRGYDEGSVA.....................................AGRTFVL.......
Thermosynechococcus                SFTIGGANSVRGWEEGAIG.....................................SGRAFVQ.......
Synechococcus8102                  AFCIGGSNSIRGWYDCDLA.....................................VARTFGE.......
Synechococcus8109                  AFCMGGSNSIRGWYDCDLA.....................................VSKAFSE.......
Nostoc7107                         AFTLGGSNSVRGYEEGTLA.....................................SSRSYVQ.......
Gloeocapsa                         AFSLGGVSSVRGFNEGDLG.....................................TGRSFVQ.......
Nostoc7120a                        AFNLGGLNSVRGYGSGKLA.....................................SGRSYGL.......
Nostoc7120b                        TFNLGGSNSVRGYDAGNVG.....................................SGRSYVL.......
Nostoc7120c                        AFTLGGSNSVRGYEEGALG.....................................SGRSFVQ.......
Prochlorococcus_marinus_1375       AFCLGGSRSIRGWSSCDLA.....................................VSRAFGE.......
Prochlorococcus_marinus_1986       AFCMGGSSSVRGWGSCDLS.....................................VSKSFAE.......
Prochlorococcus_marinus_9313       AFCLGGSNSVRGWSDCDLS.....................................VGRSFVE.......
Nostoc73102d                       AFTLGGSNSVRGYQEGGLG.....................................SGRSYIQ.......
Nostoc73102b                       SFNLGGSNSVRGYDSGDVG.....................................SGRSYVL.......
Nostoc73102a                       AFNIGGIDSVRGYGYGKVA.....................................SGRSYGL.......
Synechococcus_sp._JA-3-3Ab         AFPIGGGSSVRGFFEGGVG.....................................SGRSFAT.......
Synechococcus_sp._JA-2-3B'a        AFAIGGGNSVRGFFEGGIG.....................................SGRSFAT.......
alToc75c                           AFALGGPNSVRGYTKGELG.....................................AARNIVE.......
alToc75a                           AFVLGGPYSVRGYNMGELG.....................................AARNIAE.......
alToc75b                           AFALGGPNSVRGYSMGELG.....................................AAKHILE.......
atToc75a                           AFVLGGPYSVRGYNMGELG.....................................AARNIAE.......
psToc75                            AFTLGGPYSVRGYNMGEIG.....................................AARNILE.......
acToc75                            AFTLGGPYSVRGYNMGELG.....................................ACRNLVE.......
atToc75b                           VFALGGPNSVRGYSMGELG.....................................AAKNILE.......
atToc75c                           AFSLGGPNYVR.YTKGELG.....................................AARNIVE.......
vcToc75                            YFSLGGPYSVRGYSHGEIG.....................................AAKRFLE.......
smToc75                            AFTLGGPYSVRGYNLGELA.....................................ACRNILE.......
ppToc75a                           AFTLGGPYSVRGYNMGELG.....................................AARNFLE.......
ppToc75b                           AFTLGGPYSVRGYNMGELG.....................................AARNLLE.......
ppToc75c                           AFTLGGPYSVRGYTMGELG.....................................AARNFLE.......
zmToc75                            AFALGGPYSVRGFSNGELG.....................................ASRNILE.......
bdToc75a                           AFTLGGPYSVRGYGMGELG.....................................ASRNVLE.......
osToc75a                           AFTLGGPYSVRGYGMGELG.....................................ASRNVLE.......
osToc75b                           AFALGGPHSVRGYGMGELG.....................................ASRNLLE.......
crToc75                            YFSLGGPYSVRGYSHGEIG.....................................AARRFLE.......
bdToc75b                           TFPLGGPHSVRGYGMGELG.....................................ASRNLLEASIYITK
GsToc75                            KHRLGGMGSLRGYSTGALG.....................................TAVSSLQ.......
CmToc75                            RYPFGGLATVRGMREGSLG.....................................QVGSFAR.......
mpToc75                            AYTLGGPYSVRGYNIGELA.....................................ACRQFLE.......
pgToc75                            AFTLGGPYSVRGYNMGELG.....................................ACRNILE.......
ptaToc75                           AFTLGGPYSVRGYNMGELG.....................................ACRNILE.......
pmToc75                            AFTLGGPYSVRGYNMGELG.....................................ACRNILE.......
atrToc75                           AFTLGGPYSVRGYNMGELG.....................................ACRNILE.......
MtToc75                            AFTLGGPYSVRGYNMGEIG.....................................AARNILE.......
GmToc75                            AFTLGGPYSVRGYNMGEIG.....................................AARNILE.......
ptToc75                            AFTLGGPYSVRGYNMGELG.....................................AARNILE.......
slToc75                            AFTLGGPYSVRGYNMGEIG.....................................AARNIVE.......
hvToc75                            AFTLGGPYSVRGYGMGELG.....................................ASRNVLE.......
sbToc75                            AFALGGPYSVRGFSNGELG.....................................ASRNILE.......
NmiToc75                           VFTLGGPYSVRGYNVGELA.....................................ACRRFLE.......
mpuToc75                           AFTLGGPHSCRGYNVGELG.....................................ASRNFLE.......
csToc75                            YFTLGGPFSVRGYNVGELA.....................................ACRSFLE.......
olToc75                            AFALGGPHSCRGYTVGELG.....................................AARSLLE.......
EcBamA                             NFYAGGSSTVRGFQSNTIGPKAVYFPHQASNYDPDYDYECATQDGAKDLCKSDDAVGGNAMAV.......
EcTamA                             RFFAGGDRSIRGYKYKSIAPK................Y....ANG......DL...KGASKLI.......
NmOMP85                            NFYGGGLGSVRGYESGTLGPK.VY.....DEYGEKISY..................GGNKKAN.......
BpFhaC                             QITVGDEYTVRGYNLRTSQ.....................................SGDSGVY.......
ScSam50                            KFQSGGPSDIRGFQTFGLGPRDLY.............................DAVGGDAFVS.......
NcTob55                            RFQLGGPTDVRGFSMGGLGPH......................DGA.......DSVGGDVFAA.......
```

AtOEP80a	....
AlOEP80a	....
GmOEP80a	....
MtOEP80a	....
PsOEP80	....
SlOEP80a	....
HvOEP80a	....
SbOEP80a	....
PtOEP80a	....
AcOEP80a	....
BdOEP80a	....
OsOEP80a	....
ZmOEP80a	....
PpOEP80	....
SmOEP80a	....
SmOEP80b	....
CrOEP80	....
VcOEP80	....
MpOEP80	....
PgOEP80	....
PtaOEP80	....
PmOEP80	....
AtOEP80b	....
AlOEP80b	....
AtOEP80c	....
GmOEP80b	....
PtOEP80b	....
MtOEP80b	....
psOEP80tr	....
SlOEP80b	....
BdOEP80b	....
SbOEP80b	....
ZmOEP80b	....
OsOEP80b	....
HvOEP80b	....
AcOEP80b	....
NmiOEP80	....
CsOEP80	....
MpuOEP80	....
OlOEP80	....
AtrOEP80a	....
AtrOEP80b	....
Gloeobacter	....
Synechocystis6803	....
Cyanothece8802	....
Cyanothece51142	....
Oscillatoria	....
Microcoleus	....
Microcystis	....
Nostoc73102C	....
Anabaena_variabilis	....
Trichodesmium101	....
Thermosynechococcus	....
Synechococcus8102	....
Synechococcus8109	....
Nostoc7107	....
Gloeocapsa	....
Nostoc7120a	....
Nostoc7120b	....
Nostoc7120c	....
Prochlorococcus_marinus_1375	....
Prochlorococcus_marinus_1986	....
Prochlorococcus_marinus_9313	....
Nostoc73102d	....
Nostoc73102b	....
Nostoc73102a	....
Synechococcus_sp._JA-3-3Ab	....
Synechococcus_sp._JA-2-3B'a	....
alToc75c	....
alToc75a	....
alToc75b	....
atToc75a	....
psToc75	....
acToc75	....
atToc75b	....
atToc75c	....
vcToc75	....
smToc75	....
ppToc75a	....
ppToc75b	....
ppToc75c	....
zmToc75	....
bdToc75a	....
osToc75a	....
osToc75b	....
crToc75	....
bdToc75b	RIYKVLPIRHFSRQCHFEHVWLLPWQKMSTKQQFCCVPGQAKKDKFTGYFQAAHFVRHGLQVSNQTDPSS
GsToc75	....
CmToc75	....
mpToc75	....
pgToc75	....
ptaToc75	....
pmToc75	....
atrToc75	....
MtToc75	....
GmToc75	....
ptToc75	....
slToc75	....
hvToc75	....
sbToc75	....
NmiToc75	....
mpuToc75	....
csToc75	....
olToc75	....
EcBamA	....
EcTamA	....T.............
NmOMP85	....
BpFhaC	....
ScSam50	....YG...........
NcTob55	....

```
                                            V.                                   V.
                                  650                                660      670
AtOEP80a                          .......GSGELSFPV............RGPVEGVIFTDYGT............................
AlOEP80a                          .......GSGEMSFPV............RGPVEGVIFTDYGT............................
GmOEP80a                          .......GSGEISFPM............YGPVEGVIFSDYGT............................
MtOEP80a                          .......GSGEISFPM............MKPVECVIFSDYGT............................
PsOEP80                           .......GSGEISFPV............KRPVEAVIFSDYGT............................
SlOEP80a                          .......GCGEISFPL............MGPLEGAVFADYGT............................
HvOEP80a                          .......GSGEVSCRL............FGPLEGVVFGDYGS............................
SbOEP80a                          .......GSGEVSCRL............FGPLEGVIFGDYGS............................
PtOEP80a                          .......GSGEISFPV............LGPVEGVFFADYGT............................
AcOEP80a                          .......GSGEISFPM............FGPVEGAIFADYGS............................
BdOEP80a                          .......GSGEVSCRM............FGPLEGVVFGDYGS............................
OsOEP80a                          .......GSGEVSCRM............FGPLEGVVFGDYGS............................
ZmOEP80a                          .......GSGEVSCRM............FGPLEGVVFGDYGS............................
PpOEP80                           .......ASGEISIPL............AGPVEGAVFADYGS............................
SmOEP80a                          .......ASGEISIPL............WGPLEGAVFADYGT............................
SmOEP80b                          .......ASGEISIPL............WGPLEGAVFADYGT............................
CrOEP80                           .......AGAELRAPLP...........VQPLVATAFFDFGS............................
VcOEP80                           .......GTAELRAPLP...........LKQLCATAFVDVGS............................
MpOEP80                           .......ASGEISLPL............VGPVEGAVFADYGG............................
PgOEP80                           .......STVEISVPM............FGPVEGAIFGDYGT............................
PtaOEP80                          .......SSVEISVPM............FGPVEGAIFGDYGT............................
PmOEP80                           .......STVEISVPM............FGPLEGAIFGDYGT............................
AtOEP80b                          .......ANTELALPL............NKMTEGTIFLDCGT............................
AlOEP80b                          .......ANTELAVPL............NKMTEGTIFLDCGT............................
AtOEP80c                          .......ANTELA...............NKMTEGTIFLDCGT............................
GmOEP80b                          .......ANSELTLPL............NKMLEGAIFLDCGT............................
PtOEP80b                          .......GNSELTFPL............SKMLEGAFFVDCGT............................
MtOEP80b                          .......ANSELTLPL............TKMLEGAIFMDCGT............................
psOEP80tr                         .......ANSELTLPL............NKVLEGAVFMDCGT............................
SlOEP80b                          .......ANNELTFPL............SQVLDGAVFLDCGS............................
BdOEP80b                          .......ANCEYTVPL............AKHLEGSIFMDCGS............................
SbOEP80b                          .......ANCEYTVPL............VKHLEGSIFMDCGS............................
ZmOEP80b                          .......ANCEYTIPL............VKHLEGSIFMDCGS............................
OsOEP80b                          .......ANCEYTVPL............AKHLEGSIFMDCGS............................
HvOEP80b                          .......GNCEYTVPL............AKNLEGSLFMDRGS............................
AcOEP80b                          .......GNSELTLPL............SKMLEAAVFLDCGT............................
NmiOEP80                          .......GSMEVSVPL............FGPVEGALFSDYGT............................
CsOEP80                           .......GTAGLHWPI............FGPVDGALFFDYGT............................
MpuOEP80                          .......GSAELSVPLA...........TDTLNGYAFFDVGS............................
OlOEP80                           .......GSAEMRVPV............TPTITGAAFFDYGS............................
AtrOEP80a                         .......GSSEVSVR.......................................................
AtrOEP80b                         .......LNSELSVPL............TKMLEGTIFLDCGT............................
Gloeobacter                       .......NSVEFRAPV............FDPVGAALFVDYGT............................
Synechocystis6803                 .......ATAEYRFPI............IAAVGGALFVDYGS............................
Cyanothece8802                    .......ASAEYRFPV............IPAVGAALFFDYGT............................
Cyanothece51142                   .......ATAEYRFPI............IPAVGAAVFFDYGT............................
Oscillatoria                      .......ATAEYRFPI............ISFVGAALFIDAAT............................
Microcoleus                       .......ASVEYRFPI............ISFIGGALFLDAGT............................
Microcystis                       .......ATAEYRFPI............VAIIGGALFVDFGT............................
Nostoc73102C                      .......ASVEYRFPV............FSVVSGALFFDLGS............................
Anabaena_variabilis               .......ASVEYRFPV............FSVVSGALFFDVGS............................
Trichodesmium101                  .......GTVEYRFPV............FKFLGGALFVDAAT............................
Thermosynechococcus               .......GTVEYRFPI............FNIIGGALFVDGAS............................
Synechococcus8102                 .......VTLEYRFPI............ISVFSGELFVDAGT............................
Synechococcus8109                 .......LTLEYRFPL............ISIISGEVFMDAGT............................
Nostoc7107                        .......ATLEYRFPV............FSIVSGAAFVDFGS............................
Gloeocapsa                        .......ATAEYRFPI............FSVVGGALFVDVAS............................
Nostoc7120a                       .......ASVEYRFPI............VESIGGVVFTDFAS............................
Nostoc7120b                       .......ASAEYRFPI............VPIVGGVLFADFAS............................
Nostoc7120c                       .......ASVEYRFPV............FSVVSGALFFDVGS............................
Prochlorococcus_marinus_1375      .......ASAEYRVPI............WRMISGNVFIDGGT............................
Prochlorococcus_marinus_1986      .......ATAEYRFPV............WRMISGALFADFGS............................
Prochlorococcus_marinus_9313      .......ATIEYRFPI............WNIVSGEVFVDGGT............................
Nostoc73102d                      .......ASTEYRFPV............FSVVSGALFFDIGS............................
Nostoc73102b                      .......ASAEYRFPV............LPIVGGVLFADFAS............................
Nostoc73102a                      .......ASVEYRFPI............FQSIGGVVFTDLAS............................
Synechococcus_sp._JA-3-3Ab        .......ATAELRFPL............FDPVGAVVFADYGT............................
Synechococcus_sp._JA-2-3B'a       .......ATAELRFPI............FDPVGAVVFADYGT............................
alToc75c                          .......VGAEVRVPI.............KNTQVYAFAEHGN............................
alToc75a                          .......VGAEIRIPV.............KNTHVYAFVEHGN............................
alToc75b                          .......LSAEIRIPV.............KNTHVYTFAEHGN............................
atToc75a                          .......VGAEIRIPV.............KNTHVYAFVEHGN............................
psToc75                           .......LAAEIRIPI.............KGTHVYAFAEHGT............................
acToc75                           .......LAAELRIPV.............KNTHVYAFAEHGN............................
atToc75b                          .......LGAEIRIPV.............KNTHVYAFAEHGN............................
atToc75c                          .....LKVGAELRVNV.............KNTQGYAFAEHGN............................
vcToc75                           .......LATEVRVPL......KNF...KLPGTAYGFVEYGS............................
smToc75                           .......TAAELRVPV.............KNTHCYGFVEYGT............................
ppToc75a                          .......MGLELRVPL.............KKTHTYGFVEYGT............................
ppToc75b                          .......LAMELRVPV.............KTTHAYGFLEYGT............................
ppToc75c                          .......MALELRVPV.............KKTHAYCFAEYGT............................
zmToc75                           .......LATELRVPI.............RNTHVYAFAEHGT............................
bdToc75a                          .......VASEVRIPV.............KNTYVYGFVEHGT............................
osToc75a                          .......VASELRIPV.............RNTYVYGFVEHGT............................
osToc75b                          .......VATELSVPIT.VK........NRHTQVYAFAEHGT............................
crToc75                           .......LATEVRVPL......KNY...GLPGTAYGFVEYAT............................
bdToc75b                          ATQSVVQVAAEVRIPVPLMK........KQQTQVYAFAKHGT............................
GsToc75                           .......QTWELRFPI............VDRISGVFFYDCLD............................
CmToc75                           .......TCAEFRYPL............DKRLVFVAFTDWGSEIQPPSHLVRFPKLFRRGAHGHRLGTVV
mpToc75                           .......LATELRIPI.............NKTHAYGFLEYGS............................
pgToc75                           .......LAGEIRLPV.............KNTHTYAFVEYGT............................
ptaToc75                          .......LAGEIRLPV.............KNTHTYAFVEYGT............................
pmToc75                           .......LAGEIRLPV.............KNTHTYAFVEYGT............................
atrToc75                          .......LAAELRIPI.............RNTHVYAFAEHGN............................
MtToc75                           .......LAAEVRIPI.............KGTHVYAFAEHGT............................
GmToc75                           .......LAAELRIPV.............KGTHVYAFTEHGN............................
ptToc75                           .......LGAEVRIPV.............RNTHVYAFAEHGN............................
slToc75                           .......LAAELRIPV.............RNTHVYAFAEHGN............................
hvToc75                           .......VAGEVRVPV.............KNTYVYGFAEHGT............................
sbToc75                           .......VATELRIPV.............RNTHVYAFAEHGT............................
NmiToc75                          .......LAAEIRYPV.............MGKHVYSFYEYGT............................
mpuToc75                          .......YAAEIRVPVP...........VLNTHAFAFWERCT............................
csToc75                           .......GAVELRVPV.............LGKHVYGFYEFGT............................
olToc75                           .......TAVEIRLPVP...........VLKTHGFLFWEQCS............................
EcBamA                            .......ASLEFITPTPFISDKY.....ANSVRTSFFWDMGTVWD.TNW.....................
EcTamA                            .......GSLEYQYNV............TGKWWGAVFVDSGEAVS.........................
NmOMP85                           .......VSAELLFPMPGAKD.......ARTVRLSLFADAGSVWDGKTY.....................
BpFhaC                            .......LSNTLTVPV......QFSLLGKQASVAPFV..GA............................
ScSam50                           .......LSVFSRLPWKKVE........KSNFRLHWFFNGGKLVN..................H......
NcTob55                           .......GSVNMLLPLPRAGP.......TSPLRFQLFANGGRLVALQGKKT.........AEGSV...SL
```

T7

```
                                   680                                          690       700
AtOEP80a                      DMGSGSTVP...............................GDPAGA....RLKPGSG.YGYGLGVRVD..
AlOEP80a                      DLGSGSTVP...............................GDPAGA....RLKPGSG.YGYGLGVRVD..
GmOEP80a                      DLGSGPTVP...............................GDPAGA....RKKPGSG.YGYGFGIRVE..
MtOEP80a                      DLGSGSTVP...............................GDPAGA....RNKPGSG.YGYGLGIRVD..
PsOEP80                       DLGSGSTVL...............................GDPAGA....RNKPGSG.YGYGLGIRVD..
SlOEP80a                      DLGSGPSVP...............................GDPAGA....RLKPGSG.YGCGVGIRVE..
HvOEP80a                      DLGSGPTVP...............................GDPAGA....RGKPGSG.YGYGVGIRVD..
SbOEP80a                      DLGSGPKVP...............................GDPAGA....RGKPGSG.YGYGVGVRVD..
PtOEP80a                      DLGSGPSVP...............................GDPAGA....RLKPGSG.YGYGFGIRVD..
AcOEP80a                      DLGSGPMVP...............................GDPAGA....RLKPGSG.YGYGFGIRVD..
BdOEP80a                      DLGSGPKVP...............................GDPAGA....RGKPGSG.YGYGVGIRVD..
OsOEP80a                      DLSSGPKVP...............................GDPAGA....RGKPGSG.YGYGVGVRVD..
ZmOEP80a                      DLGSGPKVP...............................GDPAGA....RGKPGSG.YGYGVGVRVD..
PpOEP80                       DLGSGSTVP...............................GDPAGV....RGKPGNG.YGYGAGLRVD..
SmOEP80a                      DLGSGHTVP...............................GDPASV....RGKPGSG.YGYGAGIRVD..
SmOEP80b                      DLGSGHTVP...............................GDPASV....RGKPGSG.YGYGAGVRVD..
CrOEP80                       DLGSGQAVI...............................GDPAGV....RGKPGSG.YGFGGGVRMD..
VcOEP80                       DLGSGPAVI...............................GDPAGV....RGKPGSG.YGFGGGVRAD..
MpOEP80                       DLRSGKTVP...............................GDPAGQ....RFKPGSG.YGYGAGLRVD..
PgOEP80                       DFGSGQTVP...............................GDPAGA....RNKPGNG.YGYGAGIRVD..
PtaOEP80                      DFGSGQTVP...............................GDPAGA....RNKPGNG.YGYGAGIRVD..
PmOEP80                       DFGSGQTVP...............................GDPAGA....RNKPGNG.YGYGAGIRVD..
AtOEP80b                      DLGSSRLVP...............................GNPSMR....QGKPGFG.YGFGYGLRFK..
AlOEP80b                      DLGSSRLVP...............................GNPSMR....QGKPGYG.YGFGYGLRFK..
AtOEP80c                      DLGSSRLVPVSS..LYLLRTRTIKKLLRHENDKAVAELVSGNPSLR....QGKPGFG.YGFGYGLRFK..
GmOEP80b                      DLRSGHLVP...............................GNPALR....QSKPGSG.IGLGYGLRFK..
PtOEP80b                      DLGSGRLVP...............................GNPAMR....QGKPGSG.VGLGYGLRFK..
MtOEP80b                      DLRTGYLVP...............................GNPALR....HGKPGSG.VGLGYGLRFK..
psOEP80tr                     DLRSGYFVP...............................GNPALR....QGKPGSG.VGMGCGVRFK..
SlOEP80b                      DLGSGRHVP...............................GNPALR....LGKPGNG.IGFGYGLRFK..
BdOEP80b                      DLGSARHVP...............................GNPALR....QGKPGFG.AGFGYGVHFN..
SbOEP80b                      DLGSARHVP...............................GNPALR....QGKPGFG.IGFGYGLHFN..
ZmOEP80b                      DLGSARHVP...............................GNPALR....QGKPGFG.IGFGYGLHFN..
OsOEP80b                      DLGSACHVP...............................GNPALR....QGKPGFG.VGFGYGIHFN..
HvOEP80b                      DLGSARHVP...............................GNPALR....QGKPGFG.VGFGYGVHFN..
AcOEP80b                      DLGSAHHVP...............................GNPALR....QGKPGTG.IGLGYGLRLN..
NmiOEP80                      DLGSGATVP...............................GDPAGA....RGKPGKG.YGYGAGIRID..
CsOEP80                       DLDSGQTVL...............................GDPAGA....RGKPGSG.YGFGAGIRVD..
MpuOEP80                      DLKSGASVL...............................GDPGGT....RGKPGQG.YGYGLGVSAA..
OlOEP80                       DLHSGGSVL...............................GDPAGT....RGKPGSG.YSYGVAGMFE..
AtrOEP80a                     ................................................DPAGA....RGKLGSG.YGYGVGIRID..
AtrOEP80b                     DLGSGRHVP...............................GNPALR....HGKPGSG.IGLGYSLRCQ..
Gloeobacter                   TLGSQAAVL...............................GAPAIV....RGKIGAG.LGFGIGLRVQ..
Synechocystis6803             NLGSQGAVP...............................GFPAIV....RGLPGSG.VGYGLGVRIQ..
Cyanothece8802                TIKSQRSVR...............................GFPGTV....RGLPSDG.YGYGLGIRIQ..
Cyanothece51142               TIKSQRSVR...............................GIPGVV....RGLPSDG.YGYGIGVRIQ..
Oscillatoria                  DLGTGGSVP...............................GDPAGV....RGKPGSG.IGAGVGVRIQ..
Microcoleus                   DLGTGEDVP...............................GDPAGI....RGKPGSG.YGYGLGVRIQ..
Microcystis                   TIGSQGDVP...............................GQPGIV....RDLPGTG.LGYGLGVRVQ..
Nostoc73102C                  DLGTSTRA..................................AEV....LSKNGSG.YGYGLGVRVQ..
Anabaena_variabilis           DLGTSTRT..................................AEV....LNKSGSG.YGYGLGVRVQ..
Trichodesmium101              VFDSQRSVI...............................GNPGGV....REKPGDG.IGYGGGLRVN..
Thermosynechococcus           LLGTQSSVP...............................GQPGIV....RGKPGEG.LGYGAGLRVN..
Synechococcus8102             DFDTQKNVP...............................GKPGLL....LDKDGSG.VSVGTGVIVG..
Synechococcus8109             DFGTQKDVP...............................GKPGLL....LDKDGSG.VSVGTGVIVT..
Nostoc7107                    DLGTNTRA..................................AEV....LNKNGSG.YGYGIGVRVQ..
Gloeocapsa                    DLGTGENVP...............................GNPAGQ....LDKPGSG.FGYGIGLRVQ..
Nostoc7120a                   DFGSGKTVI...............................GEPGVV....RNKPGSG.FGYGLGVRLN..
Nostoc7120b                   DLGSGDTVL...............................GNPAGV....RGKPGSG.FGYGAGIRVD..
Nostoc7120c                   DLGTSTRT..................................AEV....LNKSGSG.YGYGLGVRVQ..
Prochlorococcus_marinus_1375  DLNTQKNVP...............................GNPGKL....LGKQGSG.FSIGSGLSFN..
Prochlorococcus_marinus_1986  DLGSQDDVP...............................GKPGKL....LEKSGSG.YSLGGGVGIK..
Prochlorococcus_marinus_9313  DLGSQENVP...............................GKPGKL....LDKPGSG.FSLGSGLIVT..
Nostoc73102d                  DLGSSTRS..................................AEV....LNKNGTG.YGYGLGVRVQ..
Nostoc73102b                  DLGSGDTVL...............................GDPAGV....RDKPGYG.FGYGAGVRLN..
Nostoc73102a                  DFGSSETVL...............................GEPGVL....RDKPGSG.FGYGLGLRLN..
Synechococcus_sp._JA-3-3Ab    DLGSGAAVI...............................GNPAAV....RNKPGSG.LGLGAGIRIQ..
Synechococcus_sp._JA-2-3B'a   DLGSGAAVA...............................GNPAAV....RNKPGSG.LGLGVGIRIQ..
alToc75c                      DLGSSKDVK...............................GNPTTA....FRRKGHG.SSYGVGVK....
alToc75a                      DLGSSKDVK...............................GNPTAV....YRRTGQG.SSYGAGVK....
alToc75b                      DLGSSKDVK...............................GNPTAV....YWKMGHG.SSYGLGVK....
atToc75a                      DLGSSKDVK...............................GNPTAV....YRRTGQG.SSYGAGVK....
psToc75                       DLGSSKDVK...............................GNPTVV....YRRMGQG.SSYGAGMK....
acToc75                       DLGSSKDVK...............................GNPTEF....YRRMGYG.SSYGLGVK....
atToc75b                      DLGSSKDVK...............................GNPTGL....YRKMGHG.SSYGLGVK....
atToc75c                      DLGSSKDMK...............................GNPTAA....FRREGHG.SSYGVGMK....
vcToc75                       DLGSGRELM...............................GNPTEY....YRKPGRG.MSYGVGIKA...
smToc75                       DLGSSKDVR...............................GNPTEF....FRRVGHG.GSYGVGVK....
ppToc75a                      DLGSSKDVR...............................GNPTEF....FKRAGKG.AAYGVGVK....
ppToc75b                      DLGSSKDVR...............................GNPTEF....FRRAGKG.ASYGLGVK....
ppToc75c                      DLGSSKDVR...............................GNPTEF....FRRAGKG.ASYGVGVK....
zmToc75                       DLGSSKDVK...............................GNPTEF....FRRVGHG.SSYGVGVK....
bdToc75a                      DLGSSKDVK...............................GNPTEF....FRRVGHG.SSYGVGVK....
osToc75a                      DLGSSKDVK...............................GNPTEF....FRRVGHG.SSYGLGVK....
osToc75b                      DLGSSKDVE...............................GNPTEF....FRRVGHG.SSYGVGVK....
crToc75                       DLGSGRELN...............................GNPTEY....YRKPGRG.MSYGLGLKA...
bdToc75b                      DLGSSKDVK...............................GNPTEF....FQRAGHG.SSYSVGVK....
GsToc75                       ETGS..........FW........................KHPKRL..........G.WSKGVGLRIAE.
CmToc75                       DSASAESTSSGV............................DAPASAGSIQRSSTASGVISVGLGLRV...
mpToc75                       DLGSSKDVR...............................GNPTEF....FRRAGGG.ASYGVGVK....
pgToc75                       DLGSSKDVK...............................GNPTEF....FRRVGHG.ASYGLGVK....
ptaToc75                      DLGSSKDVK...............................GNPTEF....FRRVGHG.ASYGLGVK....
pmToc75                       DLGSSKDVK...............................GNPTEF....FRRVGHG.ASYGLGVK....
atrToc75                      DLGSSKEVK...............................GNPTEF....FRRAGSG.SSYGAGVK....
MtToc75                       DLGSSKDVK...............................GNPTEV....YRRMGQG.SSYGAGMK....
GmToc75                       DLGSSKGVK...............................GNPTEV....YRRMGHG.SSYGLGVK....
ptToc75                       DLGTSKDVK...............................GNPTEV....YRRMGHG.SSYGVGVK....
slToc75                       DLGSSKDVK...............................GNPTEV....YRRMGHG.SSYGVGVK....
hvToc75                       DLGSSKDVK...............................GNPTEF....FRRVGHG.SSYGVGVK....
sbToc75                       DLGSSKDVK...............................GNPTEF....FRRVGHG.SSYGVGVK....
NmiToc75                      DLNTSKDVR...............................GNPTEF....FRRAGSG.ASWGFGVK....
mpuToc75                      DLGSSKDVK...............................GNPTEY....YRRAGSG.SSMGAGIK....
csToc75                       DLGSSKLVK...............................GNPTEY....YRRAGSG.ASLGGGVK....
olToc75                       DLSSSSALS...............................GNPTEF....YRRAGKG.STHGVGIK....
EcBamA                        DSSQYS..................................GYPD......YSDPSN..IRMSAGIALQWM
EcTamA                        DIRRSDFKT..................................................GTGVGVRWE..
NmOMP85                       DDNSSSATGGRVQNIY........................GAGNTH....KSTFTNE.LRYSAGGAVTWL
BpFhaC                        DVGALKS.................................NHPDAR....TIRMA....GLAAGVRFD..
ScSam50                       DNTSLGNCI...............................GQLSKEH..S........TSTGIGLVLR..
NcTob55                       DSGAVASGMKSA......................VAELANGLPS..............IAAGFGLVYA..
```

vi.

```
                              710       720                 730
AtOEP80a                      SPLGPLRLEYAFND..QHAG................RFHFGVGLRN........................
AlOEP80a                      SPLGPLRLEYAFND..QHAG................RFHFGVGLRN........................
GmOEP80a                      SPLGPLRLEYAFND..KQDK................RFHFGVGHRN........................
MtOEP80a                      SPLGPLRLEYAFND..KKEK................RFHFGVGYRN........................
PsOEP80                       SPLGPLRLEYAFND..KKEK................RFHFGVGYRN........................
SlOEP80a                      SPLGPLRLEYAFND..QRTG................RFHFGVGLRN........................
HvOEP80a                      SPLGPLRLEYAFND..KQAR................RFHFGVGHRN........................
SbOEP80a                      SPLGPLRLEYAFND..KQAS................RFHFGVGYRN........................
PtOEP80a                      SPLGPLRLEYAFND..RHTK................RFHFGVGHRN........................
AcOEP80a                      SPLGPLRLEYAFND..RHAR................RFHFGVGNRN........................
BdOEP80a                      SPLGPLRLEYAFND..KQAR................RFHFGVGHRN........................
OsOEP80a                      SPLGPLRLEYAFND..KQAR................RFHFGVGYRN........................
ZmOEP80a                      SPLGPLRLEYAFND..RQAR................RFHFGVGYRN........................
PpOEP80                       SPLGPLRLEYAFND..CKAR................RFHFGIGYRN........................
SmOEP80a                      SPLGPLRLEYALND..HRAK................RFHFGIGYRN........................
SmOEP80b                      SPLGPLRLEYALND..HRAK................RFHFGIGYRN........................
CrOEP80                       SPVGPVRLEYAWNA..RGEG................RFHVGLGYG.........................
VcOEP80                       SPVGPVRLEYAWNA..RKEG................RFHVGLGHRSI...HHLL................
MpOEP80                       SPLGPLRLEYAFND..QHAR................RFHFGIGYRN........................
PgOEP80                       SPLGPLRLEYALNN..QGLR................RFHFGVGYHN........................
PtaOEP80                      SPLGPLRLEYALNN..QGLR................RFHFGVGYHN........................
PmOEP80                       SPLGPLRLEYARNN..QGLR................RFHFGVGYHN........................
AtOEP80b                      SPLGHLQVDYAINA..FNQK................TLYFGVTNLA........................
AlOEP80b                      SPLGHLQVDYAMNA..FNQK................TLYFGVTNLA........................
AtOEP80c                      SPLGHLQVDYAMNA..FNQK................TLYFGITNLA........................
GmOEP80b                      SQFGHFQVDYAVNA..YQQR................TLYFGLSNLA........................
PtOEP80b                      SPAGHFQVDYAINS..FQQK................TVYFGISNLI........................
MtOEP80b                      SQFGHFQVDYAINA..FKQR................TLYFGLSNLA........................
psOEP80tr                     SQFGHFQLDYAVNA..FKQG................TFYFGLSNLA........................
SlOEP80b                      SQLGHFQVDYAVNA..FQQR................TVYFGFSNLP........................
BdOEP80b                      TDLGQIRVDYAMNA..FSRK................TIYFGINSSA........................
SbOEP80b                      TNLGQIRVDYAMNA..FNRK................TIYFGINSSG........................
ZmOEP80b                      TDLGQIRVDYAMNA..FNRK................TIYFGINSSG........................
OsOEP80b                      TDLGQIRVDYAMNA..FSRK................TIYFGINSGG........................
HvOEP80b                      TDIGQIRVDYAMNA..FSCK................TFYFSINSGG........................
AcOEP80b                      SRMGQLQVDYAINA..YQRK................TLYFNVSNVI........................
NmiOEP80                      SPLGPLRLEYAFND..KYMR................RFHFGIGYRN........................
CsOEP80                       SPVGPLRLEYAFND..KFVR................RFHFGIGSHG........................
MpuOEP80                      SPVGPLRLEYAFSD..KGVG................RMHCGIGRNF........................
OlOEP80                       TGGLPIRFDYAKND..QGNA................RFHFSLARDF........................
AtrOEP80a                     SPLGSLRLEYAFND..RHAK................RFHFGVGLRN........................
AtrOEP80b                     SRLGQIQMNYAINA..YQQK................SFSFGISNAA........................
Gloeobacter                   SPLGPLRIDFAANT..AGLPS...............QVHFGLGEKF........................
Synechocystis6803             SPVGPIRIDLGFTG..EGES................RINFGIGEKF........................
Cyanothece8802                SPVGPIRVDYGINN..EGDS................RLHFGIGERF........................
Cyanothece51142               SPVGPIRVDYGIND..EGDS................RIHFGIGERF........................
Oscillatoria                  SPLGPIRIDYGFSV..DGGS................RLHFGIGERF........................
Microcoleus                   SPLGPIRIDYGIND..TGDN................QIHFGIGERF........................
Microcystis                   SPVGQIRVDFGFNV..DGGS................RLHFGIGERF........................
Nostoc73102C                  SPLGPIRIDYGIND..DGDS................RINFGIGERF........................
Anabaena_variabilis           SPLGPIRIDYGIND..DGDS................RINFGIGERF........................
Trichodesmium101              SPLGPIRIDYAIND..EGDT................RFHFGIGERF........................
Thermosynechococcus           TPLGNIRIDFGWNN..QGGS................AFSFGIGERF........................
Synechococcus8102             TPVGPLRLEVASKD.FTDDW................RFNLGVGWKF........................
Synechococcus8109             TPVGPIRLEVATKD.FSSDY................RFNLGVGWKF........................
Nostoc7107                    SPLGPIRIDYGIND..DGDS................RINFGIGERF........................
Gloeocapsa                    SPLGPLRIDYGFNS..EGEN................RLHFGIGERF........................
Nostoc7120a                   SPFGLFRGDLGVSD..QGEV................RFEITTGQRF........................
Nostoc7120b                   SPLGLIRADYGIND..QGES................RVHLGIGQRF........................
Nostoc7120c                   SPLGPIRIDYGIND..DGDS................RINFGIGERF........................
Prochlorococcus_marinus_1375  TPVGPLRIEAASKD.LEGDW................RYNVGFGWKF........................
Prochlorococcus_marinus_1986  TPIGPLRLDVASKD.LSGDW................RYNLGVGWKF........................
Prochlorococcus_marinus_9313  TPVGPLRLEVATQD.FTDEW................RFNLGVGWKF........................
Nostoc73102d                  SPLGPIRIDYGING..DGDN................RINFGIGERF........................
Nostoc73102b                  SPLGLIRADYGIND..QGES................KVHLGIGQRF........................
Nostoc73102a                  SPFGLIRGDLGISD..QGEV................RFEVTTGQRF........................
Synechococcus_sp._JA-3-3Ab    SPLGPLRIDYAIGQGEAGRP................QLHFGIGEKF........................
Synechococcus_sp._JA-2-3B'a   SPLGPLRIDYAVGQGGAGQG................QLHFGIGEKF........................
alToc75c                      ..LGQIRAEYAVDH.NCGTG................ALSLQFGERY........................
alToc75a                      ..LGLVRAEYAIDH.NNGTG................ALFFRFGERY........................
alToc75b                      ..LGKVRAEYTVRH.NRGTG................ALFLRFGERY........................
atToc75a                      ..LGLVRAEYAVDH.NNGTG................ALFFRFGERY........................
psToc75                       ..LGLVRAEYAVDH.NSGTG................AVFFRFGERF........................
acToc75                       ..LGLVRAEYAVDH.NSGTG................AIFLRFGERF........................
atToc75b                      ..LGMVRAEYTVRH.NRGTG................ALFLRFGERY........................
atToc75c                      ..LG..................................................................
vcToc75                       ..LGACRFEYARDC.NAGSG................TLLVNFGERF........................
smToc75                       ..LGLVRAEYARDC.NAGSG................ALYVRFGERF........................
ppToc75a                      ..LGSIRAEYARDC.NAGSG................ALFVRFGERF........................
ppToc75b                      ..LGTIRAEYARDC.NAGTG................AIFFRFGERF........................
ppToc75c                      ..LGSIRTEYARDC.NTDTG................AIFVRFGERF........................
zmToc75                       ..LGLVRGEYIVDH.NAGTG................TVFFRFGERF........................
bdToc75a                      ..LGLVRGEYIVDH.NAGAG................TIFFRFGERF........................
osToc75a                      ..LGLVRGEYIVDH.NAGTG................TVFFRFGERF........................
osToc75b                      ..LGAVRAEYAVDH.NAGTG................AFFLRFGERF........................
crToc75                       ..LGACRFEYARDC.NAGTG................TFLVNFGERF........................
bdToc75b                      ..LGPIRAEYAVDH.NAGTG................ALFLRFGERF........................
GsToc75                       ....MIRLDYAWKQ..DGSG................MMHVEMINSFFILYDHTMDDDAIQVERELAIRKR
CmToc75                       ..LGALRMECGWIL.DRAPGGSGGGFPNWRLSRDG.YWHIGLVDVSY.......................
mpToc75                       ..LGSIRAEYAQDC.NAGSG................SLFVRFGERF........................
pgToc75                       ..LGLMRAEYAVDH.NAGTG................SVLVRFGERF........................
ptaToc75                      ..LGLMRAEYAVDH.NAGTG................SVLVRFGERF........................
pmToc75                       ..LGLMRAEYAVDH.NARTG................SVLVRFGERF........................
atrToc75                      ..LGLVRAEYAVDH.NSGTG................AIFFRFGERF........................
MtToc75                       ..LGLVRAEYAVDH.NSGTG................AIFFRFGERF........................
GmToc75                       ..LGLVRAEYAVDH.NSGTG................ALFFRFGERF........................
ptToc75                       ..LGLVRAEYAVDH.NTGTG................SVFFRFGERY........................
slToc75                       ..LGLVRAEYAVDH.NSGTG................AVFFRFGERF........................
hvToc75                       ..LGLVRGEYIVDH.NTGAG................TVFFRFGERF........................
sbToc75                       ..LGLVRGEYIVDH.NAGTG................TIFFRFGERF........................
NmiToc75                      ..LGAVRAEYARDC.NAGTG................ALFVRFGERF........................
mpuToc75                      ..LGPVRAEYATDG.NQANG................TAFVRFGERF........................
csToc75                       ..LGAVRLEYATDA.NTGQG................NIFVRFGERF........................
olToc75                       ..LGPVRAEWCIDN.NSGTA................STYVRFGERF........................
EcBamA                        SPLGPLVFSYAQPF.KKYDGDKAE............QFQFNIGKTW........................
EcTamA                        SPVGPIKLDFAVPV..............ADKDEHGLQFYIGLGPEL........................
NmOMP85                       SPLGPMKFRYAYPL.KKKPEDEIQ............RFQFQLGTTF........................
BpFhaC                        LPYARMSFTYSKPV.GAQPGGAPR.APV........WLYINAGLSF........................
ScSam50                       HPMARFELNFTLPI.TAHENDLIRK...........GFQFGLGLAFL.......................
NcTob55                       HPVARFELNFSLPL.VVRRGEEARK...........GLQVGVGINFL.......................
```

```
AtOEP80a                          ......................................................................
AlOEP80a                          ......................................................................
GmOEP80a                          ......................................................................
MtOEP80a                          ......................................................................
PsOEP80                           ......................................................................
SlOEP80a                          ......................................................................
HvOEP80a                          ......................................................................
SbOEP80a                          ......................................................................
PtOEP80a                          ......................................................................
AcOEP80a                          ......................................................................
BdOEP80a                          ......................................................................
OsOEP80a                          ......................................................................
ZmOEP80a                          ......................................................................
PpOEP80                           ......................................................................
SmOEP80a                          ......................................................................
SmOEP80b                          ......................................................................
CrOEP80                           ......................................................................
VcOEP80                           ...........FNAPAYSHD.....................QEPTSKRCRF..............ALPPH
MpOEP80                           ......................................................................
PgOEP80                           ......................................................................
PtaOEP80                          ......................................................................
PmOEP80                           ......................................................................
AtOEP80b                          ......................................................................
AlOEP80b                          ......................................................................
AtOEP80c                          ......................................................................
GmOEP80b                          ......................................................................
PtOEP80b                          ......................................................................
MtOEP80b                          ......................................................................
psOEP80tr                         ......................................................................
SlOEP80b                          ......................................................................
BdOEP80b                          ......................................................................
SbOEP80b                          ......................................................................
ZmOEP80b                          ......................................................................
OsOEP80b                          ......................................................................
HvOEP80b                          ......................................................................
AcOEP80b                          ......................................................................
NmiOEP80                          ......................................................................
CsOEP80                           ......................................................................
MpuOEP80                          ......................................................................
OlOEP80                           ......................................................................
AtrOEP80a                         ......................................................................
AtrOEP80b                         ......................................................................
Gloeobacter                       ......................................................................
Synechocystis6803                 ......................................................................
Cyanothece8802                    ......................................................................
Cyanothece51142                   ......................................................................
Oscillatoria                      ......................................................................
Microcoleus                       ......................................................................
Microcystis                       ......................................................................
Nostoc73102C                      ......................................................................
Anabaena_variabilis               ......................................................................
Trichodesmium101                  ......................................................................
Thermosynechococcus               ......................................................................
Synechococcus8102                 ......................................................................
Synechococcus8109                 ......................................................................
Nostoc7107                        ......................................................................
Gloeocapsa                        ......................................................................
Nostoc7120a                       ......................................................................
Nostoc7120b                       ......................................................................
Nostoc7120c                       ......................................................................
Prochlorococcus_marinus_1375      ......................................................................
Prochlorococcus_marinus_1986      ......................................................................
Prochlorococcus_marinus_9313      ......................................................................
Nostoc73102d                      ......................................................................
Nostoc73102b                      ......................................................................
Nostoc73102a                      ......................................................................
Synechococcus_sp._JA-3-3Ab        ......................................................................
Synechococcus_sp._JA-2-3B'a       ......................................................................
alToc75c                          ......................................................................
alToc75a                          ......................................................................
alToc75b                          ......................................................................
atToc75a                          ......................................................................
psToc75                           ......................................................................
acToc75                           ......................................................................
atToc75b                          ......................................................................
atToc75c                          ......................................................................
vcToc75                           ......................................................................
smToc75                           ......................................................................
ppToc75a                          ......................................................................
ppToc75b                          ......................................................................
ppToc75c                          ......................................................................
zmToc75                           ......................................................................
bdToc75a                          ......................................................................
osToc75a                          ......................................................................
osToc75b                          ......................................................................
crToc75                           ......................................................................
bdToc75b                          ......................................................................
GsToc75                           LGKIFKRKREDFNTEEEFNDYLEAFEDVVYQLSEGTNSDETQESMEKLKKYIKDTGRQDILENVQILDTE
CmToc75                           ......................................................................
mpToc75                           ......................................................................
pgToc75                           ......................................................................
ptaToc75                          ......................................................................
pmToc75                           ......................................................................
atrToc75                          ......................................................................
MtToc75                           ......................................................................
GmToc75                           ......................................................................
ptToc75                           ......................................................................
slToc75                           ......................................................................
hvToc75                           ......................................................................
sbToc75                           ......................................................................
NmiToc75                          ......................................................................
mpuToc75                          ......................................................................
csToc75                           ......................................................................
olToc75                           ......................................................................
EcBamA                            ......................................................................
EcTamA                            ......................................................................
NmOMP85                           ......................................................................
BpFhaC                            ......................................................................
ScSam50                           ......................................................................
NcTob55                           ......................................................................
```

**AtOEP80a**	....
**AlOEP80a**	....
**GmOEP80a**	....
**MtOEP80a**	....
**PsOEP80**	....
**SlOEP80a**	....
**HvOEP80a**	....
**SbOEP80a**	....
**PtOEP80a**	....
**AcOEP80a**	....
**BdOEP80a**	....
**OsOEP80a**	....
**ZmOEP80a**	....
**PpOEP80**	....
**SmOEP80a**	....
**SmOEP80b**	....
**CrOEP80**	....
**VcOEP80**	EHERL........SRCHMASKQAASKCQAT
**MpOEP80**	....
**PgOEP80**	....
**PtaOEP80**	....
**PmOEP80**	....
**AtOEP80b**	........................SS...T
**AlOEP80b**	........................SS...T
**AtOEP80c**	........................SS...T
**GmOEP80b**	........................S.....
**PtOEP80b**	....
**MtOEP80b**	........................S.....
**psOEP80tr**	........................S.....
**SlOEP80b**	........................S.....
**BdOEP80b**	.......................RS.....
**SbOEP80b**	.......................GS.....
**ZmOEP80b**	.......................GS.....
**OsOEP80b**	.......................GS.....
**HvOEP80b**	......................AGS.....
**AcOEP80b**	........................S.....
**NmiOEP80**	....
**CsOEP80**	....
**MpuOEP80**	....
**OlOEP80**	.N............................
**AtrOEP80a**	....
**AtrOEP80b**	........................F.....
**Gloeobacter**	....
**Synechocystis6803**	....
**Cyanothece8802**	....
**Cyanothece51142**	....
**Oscillatoria**	....
**Microcoleus**	....
**Microcystis**	....
**Nostoc73102C**	....
**Anabaena_variabilis**	....
**Trichodesmium101**	....
**Thermosynechococcus**	....
**Synechococcus8102**	....
**Synechococcus8109**	....
**Nostoc7107**	....
**Gloeocapsa**	....
**Nostoc7120a**	....
**Nostoc7120b**	....
**Nostoc7120c**	....
**Prochlorococcus_marinus_1375**	....
**Prochlorococcus_marinus_1986**	....
**Prochlorococcus_marinus_9313**	....
**Nostoc73102d**	....
**Nostoc73102b**	....
**Nostoc73102a**	....
**Synechococcus_sp._JA-3-3Ab**	....
**Synechococcus_sp._JA-2-3B'a**	....
**alToc75c**	....
**alToc75a**	....
**alToc75b**	....
**atToc75a**	....
**psToc75**	....
**acToc75**	....
**atToc75b**	....
**atToc75c**	....
**vcToc75**	....
**smToc75**	....
**ppToc75a**	....
**ppToc75b**	....
**ppToc75c**	....
**zmToc75**	....
**bdToc75a**	....
**osToc75a**	....
**osToc75b**	....
**crToc75**	....
**bdToc75b**	....
**GsToc75**	NNEQLPSFIFLDPAR...PAKPMPSF..RI
**CmToc75**	....
**mpToc75**	....
**pgToc75**	....
**ptaToc75**	....
**pmToc75**	....
**atrToc75**	....
**MtToc75**	....
**GmToc75**	....
**ptToc75**	....
**slToc75**	....
**hvToc75**	....
**sbToc75**	....
**NmiToc75**	....
**mpuToc75**	....
**csToc75**	....
**olToc75**	....
**EcBamA**	....
**EcTamA**	....
**NmOMP85**	....
**BpFhaC**	....
**ScSam50**	....
**NcTob55**	....
