# Supplementary material for: Evolution and targeting of Omp85 homologs in the chloroplast outer envelope membrane
Source: Front Plant Sci. 2014 Oct 13;5:535. doi: 10.3389/fpls.2014.00535 (PMC4195282; doi:10.3389/fpls.2014.00535)
Supplement: Figure S2 — Energy requirement for import of truncated chloroplast Omp85 homologs in vitro. [file DataSheet2.PDF]

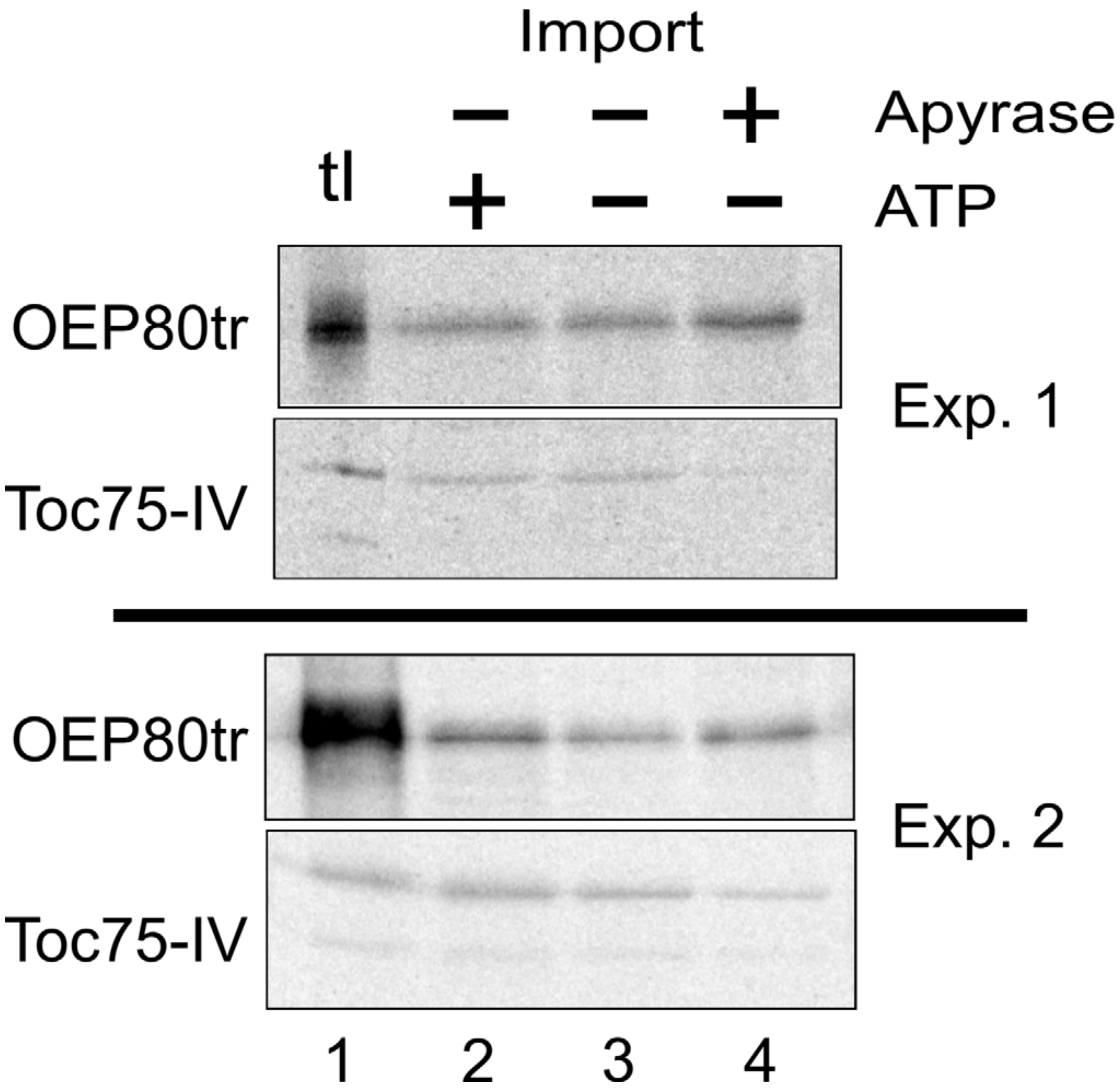

Figure S2. Energy requirement for import of truncated chloroplast Omp85 homologs *in vitro*.

Translation products including the radiolabeled proteins indicated at left (tl) were incubated without (-) or with (+) apyrase at room temperature for 15 min. The resultant samples were incubated with isolated chloroplasts with (+) or without (-) 3 mM MgATP for 30 min at room temperature in the dark. Chloroplasts were re-isolated and examined directly without fractionation as described in Fig. 4A. Results of two independent experiments (Exp.1 and Exp. 2) are shown.
